# Selective recognition and extraction of iodide from pure water by a tripodal selenoimidazol(ium)-based chalcogen bonding receptor

**DOI:** 10.1016/j.isci.2024.108917

**Published:** 2024-01-15

**Authors:** Abu S.M. Islam, Sourav Pramanik, Sahidul Mondal, Rajib Ghosh, Pradyut Ghosh

**Affiliations:** 1 School of Chemical Sciences, Indian Association for the Cultivation of Science, Kolkata 700032, India

**Keywords:** Natural sciences, Chemistry, Inorganic chemistry

## Abstract

A selenium-based tripodal chalcogen bond (ChB) donor TPI-3Se is demonstrated for the recognition and extraction of I^−^ from 100% water medium. NMR and ITC studies with the halides reveal that the ChB donor selectively binds with the large, weakly hydrated I^−^. Interestingly, I^−^ crystallizes out selectively in the presence of other halides supporting the superiority of the selective recognition of I^−^. The X-ray structure of the ChB-iodide complex manifests both the *μ*_*1*_ and *μ*_*2*_ coordinated interactions, which is rare in the C–Se···I chalcogen bonding. Furthermore, to validate the selective I^−^ binding potency of TPI-3Se in pure water, comparisons are made with its hydrogen and halogen bond donor analogs. The computational analysis also provides the mode of I^−^ recognition by TPI-3Se. Importantly, this receptor is capable of extracting I^−^ from pure water through selenium sigma-hole and I^−^ interaction with a high degree of efficiency (∼70%).

## Highlights

• ChB receptor selectively recognizes and extracts the I^−^ from 100% water medium
• In solid state, C-Se···I ChB interactions exhibit both the *µ_1_* and *µ_2_* coordination
• DFT studies are also used to corroborate the experimental observations

## Graphical abstract

**Figure:**
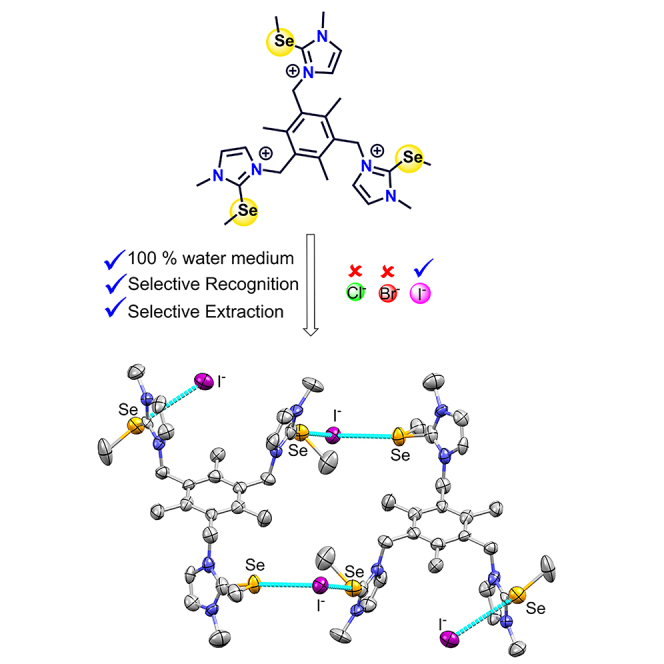

## Introduction

Majority of the reported abiotic anion receptors are found to be operative in organic solvents and most of the receptors have been designed by employing non-covalent interactions, such as hydrogen bonding (HB),^1^^,^^2^ halogen bonding (XB),^3^^,^^4^ and anion−π interactions.^5^ Due to the large hydration energies of anions, the extraction of anions in pure water is one of the key challenges in supramolecular chemistry. Iodide (I^−^) is among one of the most important anions that regulate the neurological activities and thyroid gland functions of the human body.^6^ It also behaves as a hazardous pollutant, mostly as a radioisotope which mainly comes from the nuclear fission of fissile nuclei U-235 and a large number of medical institutes.^7^^,^^8^^,^^9^ The produced radioactive iodide wastes, ^129^I and ^131^I have high radioactivity, long half-lives, and toxicity, which can potentially affect human health such as the liver, kidney, and may also culminate to thyroid cancer.^10^^,^^11^^,^^12^ High solubility of I^−^ salts in water causes severe contamination of radioactive iodine in seawater, groundwater, and soil that possess a high risk to public health and the environment.^10^^,^^11^^,^^12^^,^^13^ Thus, some constructive approaches for recognizing iodide are steadily thriving.^14^^,^^15^^,^^16^^,^^17^^,^^18^^,^^19^ Despite this, it is essential to develop a remediation process to effectively remove I^−^ from contaminated environments. Various conventional methods for ion capture and environmental remediation include chemical precipitation, solid-liquid as well as liquid-liquid extraction, ion exchange resins, and adsorption onto activated carbon.^20^^,^^21^ But among these methods chemical precipitation is considered one of the most effective and mature method for anion extraction due to its unique advantages in terms of selectivity, simplicity, cost-effectiveness, and scalability. In particular, for wastewater treatment in industry, this effective tool has been widely used. However, it may have some limitations, especially in dealing with complex sample matrices and the potential for contamination. Notwithstanding, anion extraction is a critical step, so the choice of extraction methods should depend on the specific requirements of the application and careful consideration of the advantages and limitations.

In recent years, compared to conventional HB and XB, two sigma-hole-based chalcogen bond (ChB) interactions have gained popularity in anion recognition chemistry.^22^^,^^23^^,^^24^^,^^25^^,^^26^^,^^27^^,^^28^ Concretely, the σ-hole interactions are mainly composed by the halogen (HX), chalcogen (ChB), pnictogen (PnB),^29^^,^^30^ and tetrel (TrB) bonding^31^ associated with the groups 17, 16, 15, and 14 elements, respectively. Such, highly directional non-covalent interactions indeed occur between the electropositive region (σ-hole) of the atoms described previously and an electron-rich atom (Lewis base). The potency of σ-hole can also be regulated by increasing the polarizability and decreasing the electronegativity of these atoms. Sustained research related to these interactions is attracting growing prominence and has been contextualized in different fields, including supramolecular chemistry,^22^ materials science,^32^^,^^33^ and biochemistry,^34^ among others.

In analogy with the HX, the sister ChB originated from the (n→σ∗) charge transfer interaction between the σ-hole containing elements (S, Se, and Te) and an electron-rich atom or group of atoms.^35^^,^^36^^,^^37^^,^^38^ The presence of two highly directional σ-holes (opposite to two covalent bonds) in the ChB donor represents the strong ChB interaction compared to HB and in contrast to XB, ChB donor atoms exhibit a greater electropositivity, as well as possess contrasting steric and geometric diversity for host-guest interactions.^38^^,^^39^ Thus, the ChB non-covalent interaction provides high binding opportunities as well as fine-tuning options to enable more precise three-dimensional spatial regulation for anion binding. Additionally, electrostatic attraction, charge transfer, polarization, and dispersion have been recognized as potential factors that contribute to the attractive character of ChB interactions.^40^ Furthermore, compared to the HB, in an analogous manner to the XB interaction, the ChB also procures higher hydrophobicity and less sensitivity to the solvent and pH of the systems.^25^^,^^36^^,^^38^^,^^41^^,^^42^ Hence, these unique idiosyncrasies of ChBs have extensively been exploited in catalysis,^43^^,^^44^^,^^45^^,^^46^ pharmaceutics,^47^ crystal engineering,^48^^,^^49^^,^^50^ anion transport,^24^^,^^51^^,^^52^ and self-assembly processes.^53^^,^^54^ In fact, in the last few years, with the success in developments of XB area, the interest in utilizing ChB intermolecularly in solution has gradually gained prominence. Recent studies have demonstrated that selenium (Se)-based ChB container assemblies are stable in the water medium, where selenium (Se) renders a capsule lining that complements the guest C−H bonds.^55^ In addition, to defining the bulk material property and the nano-scale application, ChB plays an important role.^56^ As a consequence of the application of selenium-based ChB, Matile, and Huber groups have extended the non-covalent interaction toward the intermolecular Lewis acids in organic synthesis and organocatalysis.^56^ Furthermore, ChBs involving selenium have also served in the important biochemical reaction of ebselen^57^ and even abet in biological host-guest recognition.^58^ Hence, selenium-based ChB receptors have a wide range of practical implications and potential applications in diverse fields, from environmental science and medicinal to materials science and chemical research. Their relevance lies in their ability to selectively bind to specific molecules or ions, enabling the development of more targeted and efficient processes and technologies.

However, in the context of, anion recognition in water through ChB interactions is an undeveloped area of host-guest chemistry.^22^^,^^23^^,^^24^^,^^25^^,^^26^^,^^27^^,^^28^^,^^59^ Beer et al. have studied seleno- and tellurotriazole chalcogen-based systems for halide recognition in organic and organoaqueous media.^60^^,^^61^ Taylor et al. have also reported ChB-based hosts for halide recognition in organic solvents.^62^^,^^63^ Caballero et al. also described a chalcogen-based receptor for recognizing chloride and bromide in THF.^64^ However, to the best of our knowledge, to date there is only one tellurotriazole-based chalcogen bonding receptor known for the recognition of I^−^ in pure water.^25^ Furthermore, utilizing the tripodal-based system for anion recognition, previously we have reported an iodo-imidazolium (XB) and protic-imidazolium (HB) receptors for Br^−^ (bromide) recognition over Cl^−^ (chloride) in CH_3_CN.^65^ In addition, Beer et al. also developed tris-triazolium (XB/HB) receptors for the selective recognition of Cl^−^ in DMSO solution.^66^ Herein we first time report a selenoimmidazol(ium)-based tri-cationic ChB donor motif (TPI-3Se) for the selective recognition along with the extraction of the sodium salt of I^−^ in 100% water medium. Further, we have also modified the receptor to its HB (TPI-3H) and XB (TPI-3I) congener in order to investigate the selective I^−^ binding potency and demonstrate that TPI-3Se receptor is superior for I^−^. It is noteworthy that, such imidazole containing tripodal host for I^−^ extraction from water is unprecedented. Moreover, structural evidence of ChB between Se and I^−^ exhibits both the *μ*_*1*_ and *μ*_*2*_ coordination modes simultaneously, which is indeed rarely observed. The mode of I^−^ binding with TPI-3Se is also investigated theoretically by using DFT calculations with molecular electrostatic potential surface and NBO analysis. Consequently, this work constitutes a rare example of selenium-based ChB-mediated anion extraction as well as offers a great scope toward developing novel ChB-based congeners with increasing the number of interaction site for the application of wastewater polishing and in host-guest chemistry.

## Results and discussion

### Receptors synthesis

Synthesis of the target tripodal-based chalcogen host (TPI-3Se) is depicted in Scheme 1. Selenation is achieved with potassium carbonate and elemental selenium.^67^ The desired cationic ChB receptor TPI-3Se is obtained with high yields upon alkylation with methyl triflate and characterized by different spectroscopic methods such as ^1^H, ^13^C, ^19^F, ^77^Se-NMR, and HRMS (see the supplemental information) (Scheme S1 and Figures S1–S11). In this work, further analogous HB (TPI-3H) and XB (TPI-3I) receptors are also prepared according to the reported method^65^ followed by anion exchange with aqueous AgOtf (Figures S12–S17).

**Scheme 1:**
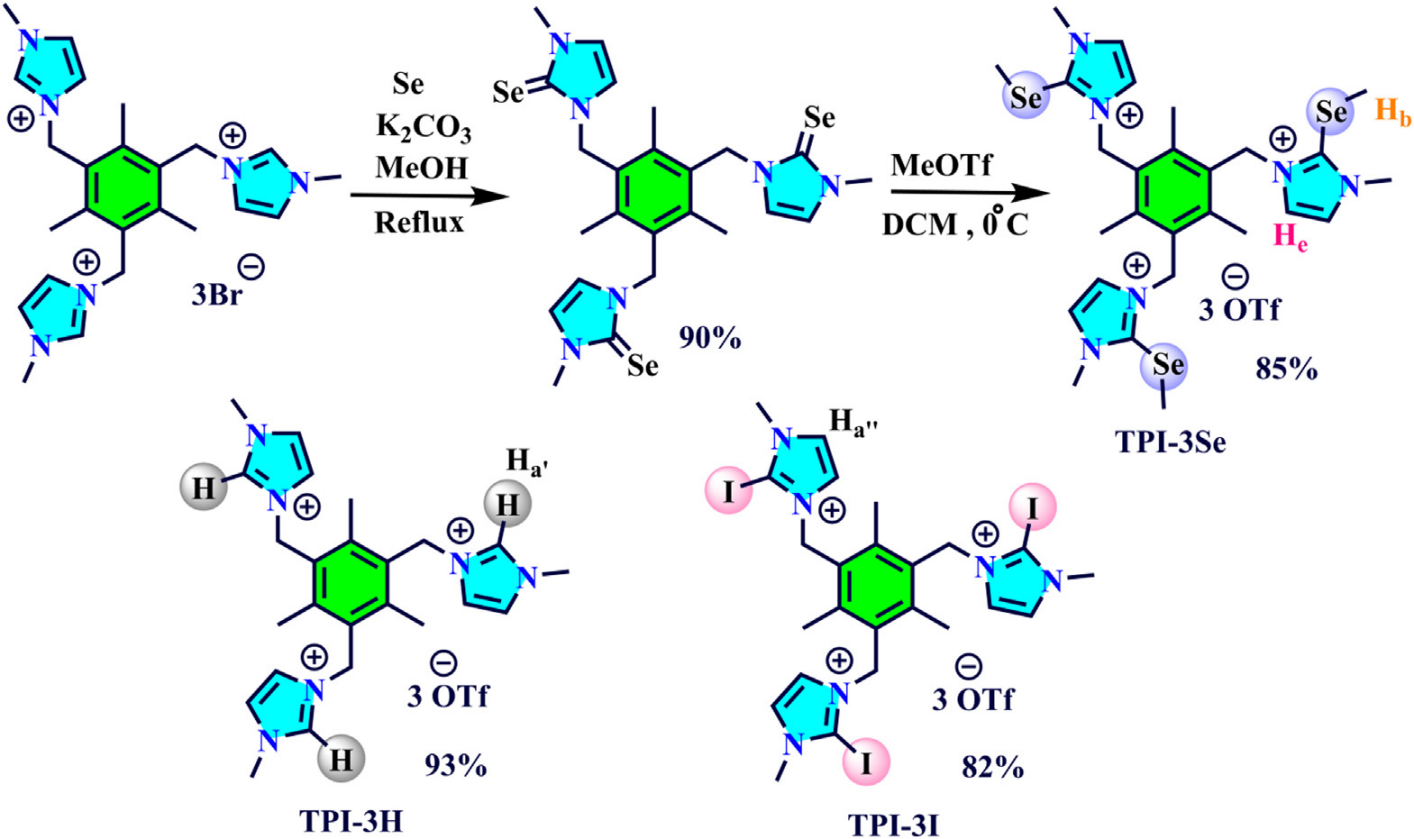
Synthesis route of ChB anion receptor TPI-3Se and structures of analogous TPI-3H and TPI-3I

### Solution phase halides binding by NMR studies

The anion-binding affinities of halides have been assessed by ^1^H, ^13^C, and ^77^Se-NMR titration experiments with TPI-3Se in D_2_O. The gradual addition of sodium salt of halides, the resulting downfield chemical shifts corresponding to the peak Se-CH_3_ (H_b_) and one imidazole proton (H_e_) are observed in ^1^H-NMR studies. Relatively higher downfield shift Δδ = 0.029 ppm of H_b_ proton is observed upon the addition of ∼8 equivalents of I^−^ (Figure S18) compared to Cl^−^ (0.011 ppm) and Br^−^ (0.010 ppm). In the case of H_e_ proton 0.056, 0.017, and 0.022 ppm Δδ are observed for I^−^, Br^−^, and Cl^−^, respectively (Figures S18–S20). This study indicates that the H_b_ and H_e_ protons are engaged in substantial hydrogen bonding interactions with I^−^ as compared to Cl^−^ and Br^−^. A well-fitted 1:1 and 1:2 binding isotherm in a Bindfit analysis (Figures S21–S26) provides the association constants of halides with the TPI-3Se (Tables 1 and S1). The high association constant for I^−^ reveals that the I^−^ binds to the tris-imidazolium cleft selectively through Se-CH_3_ units of TPI-3Se ChB donor.

**Table 1:** Association constants of halides (K_I_/M^−1^) for TPI-3Se, TPI-3I, and TPI-3H

Anion	*K*_*I*_/M^−1^ (D_2_O) TPI-3Se	*K*_*I*_/M^−1^ (1:1 D_2_O:CD_3_CN) TPI-3Se	*K*_*I*_/M^−1^ (1:1 D_2_O:CD_3_CN) TPI-3I	*K*_*I*_/M^−1^ (1:1 D_2_O:CD_3_CN) TPI-3H
Cl^−^	13 ± 4%	33 ± 4%	40 ± 6%	191 ± 5%
Br^−^	87 ± 9%	73 ± 9%	348 ± 16%	416 ± 8%
I^−^	214 ± 7%	357 ± 7%	39 ± 6%	51 ± 2%

In contrast with the halides, the addition of other hydrophobic anions (∼8 equivalents) such as ReO_4_^−^, ClO_4_^−^, and PF_6_^−^ have shown insignificant protons shift in ^1^H-NMR titration, which implies that the receptor TPI-3Se forms the strongest complex with I^−^ only (Figures S27–S29). Again, for additional evidence to support the proposed selective I^−^ binding in water, we have performed the ^1^H-NMR titrations in D_2_O:CD_3_CN (1:1) binary solvent (Figures S30–S35). The results clearly exhibit the same selectivity trend as observed in pure water (Table 1).

Moreover, to check the superiority of the recognition of I^−^ through TPI-3Se over its HB (TPI-3H) and XB (TPI-3I) congeners, analogous qualitative ^1^H-NMR titrations are also performed. In the case of HB, after the addition of ∼8 equivalents of halide ions in D_2_O:CD_3_CN (1:1) solvent, a prominent downfield perturbation is observed in the acidic proton (H_a’_) of the azolium ring, while for XB, imidazole protons shows significant chemical shifts (Figure S36–S47). Therefore, by monitoring the proton chemical shifts of both the receptors with halide anions concentration, 1:1 Bindfit analysis elicits that the binding constant of Br^−^ is higher compared to Cl^−^ and I^−^ (Figures S39–S41 and S45–S47, and Table 1). This notable result suggests that the ChB receptor TPI-3Se is the most potent receptor for selective I^−^ recognition in pure water compared to its XB and HB analogs.

It is noteworthy that, during ^13^C-NMR titration of TPI-3Se (72.84 mM) with I^−^ (87.40 mM) in 0.45 mL D_2_O, immediate precipitation of the host-guest adduct is noticed. However, such precipitation is not at all observed even upon the addition of 10 equivalents (excess) of Cl^−^ or Br^−^ to the TPI-3Se. Similar, observation is also perceived during ^77^Se-NMR titration in D_2_O. Such accidental finding of selective precipitation of I^−^ with TPI-3Se in water leads us to develop an easier pathway for the extraction of I^−^ through precipitation (*vide infra*). Hence, ^13^C and ^77^Se-NMR investigations are carried out in 1:1 D_2_O:CD_3_CN binary solvents. In the ^13^C-NMR titration of TPI-3Se (53.72 mM) with the addition of I^−^ (357.49 mM), a large downfield chemical shift Δδ = 0.54 ppm of Se-CH_3_ carbon is observed (Figure S48). In cases of Br^−^ and Cl^−^, very low chemical shifts Δδ = 0.13 and −0.02 ppm respectively are accounted upon the addition of ∼8 equivalents of respective halides (Figures S48–S50). This depicts that the Se-CH_3_ subunits of TPI-3Se exert significant influence on the I^−^ binding. Such interaction with I^−^ is further established by ^77^Se-NMR experiments, which show a large downfield chemical shift (Δδ = 1.14 ppm) in the case of I^−^ compared to Cl^−^ (Δδ = 0.15 ppm) and Br^−^ (Δδ = 0.29 ppm) upon addition of ∼8 equivalents of halides (Figures S51–S53). Alongside, in the presence of iodide-interfering pseudohalide CN^−^ (∼8 equivalents) no obvious shift in the Se peak is observed (Figure S54). Therefore, in the solution phase, these observations are consistent with the involvement of the ChB interaction between the σ-hole of the selenium and I^−^. Furthermore, a comparison of the halide binding selectivity between the ChB receptor TPI-3Se and previously reported tripodal-based halogen and hydrogen systems reveals the fundamental differences in sensitivity to anion basicity.^65^^,^^66^^,^^68^ The halide binding propensity for TPI-3Se in water medium is found to be in the order Cl^−^ < Br^−^ < I^−^, which could be due to the intrinsic preference of selenium’s σ-holes toward the softer, more lipophilic, and easily desolvated higher homolog of halides.^39^^,^^60^^,^^61^^,^^69^

### Thermodynamic contributions to halide ions binding

Having demonstrated the importance of the ChB donor for selective halide recognition, isothermal titration calorimetric (ITC) studies are carried out to offer further insights into the thermodynamic enthalpic and entropic contributions behind the halides binding with TPI-3Se. Titration of sodium halides (I^−^, Br^−^, and Cl^−^) with TPI-3Se in H_2_O:CH_3_CN (1:1) illustrates smooth and clear exothermic heat changes which are well fitted to a sequential 1:3 receptor-anion binding mode (Figures S55–S57). Notable to mention that, in the case of TPI-3H and TPI-3I in the same binary solvent led to the precipitation problem for all halides; therefore, we are unable to quantify the ITC studies. For TPI-3Se, inspection of Table 2 divulges that the binding of halides is a high enthalpy (ΔH) driven process. Moreover, the calculated 1:3 cumulative association constants (β_3_) for I^−^, Br^−^, and Cl^−^ become 22.1 x 10^11^, 8.96 x 10^11^, and 4.16 x 10^11^ M^−3^, respectively, suggesting that the ascertains of higher binding affinity toward I^−^. The larger association constant for I^−^ is primarily driven by a higher enthalpic contribution for the exothermic binding phenomenon, which can be attributed to selenium’s σ-holes’ inherent affinity for softer I^−^.

**Table 2:** Thermodynamic parameters of halides binding determine by fitting 1 : 3 sequential site model from ITC studies

Anion	β_3_ [M^−3^]	K_1_ [M^−1^]	K_2_ [M^−1^]	K_3_ [M^−1^]	ΔH [kJ/mol]	TΔS [kJ/mol]
Cl^−^	4.16 (±0.01) x10^11^	8.10 (±0.78) x 10^4^	7.19 (±0.32) x 10^4^	7.15 (±0.38) x 10^1^	−66.4 (2.7)	+0.38
Br^−^	8.96(±0.16) x10^11^	1.4 (±0.13) x 10^5^	9.93 (±0.49) x 10^4^	6.45 (±0.26) x 10^1^	−70.5 (2.3)	−1.87
I^−^	22.12(±0.66) x10^11^	5.01 (±0.3) x 10^5^	9.81 (±0.34) x 10^4^	4.5 (±0.94) x 10^1^	−84.5 (1.4)	−13.6

### Solid state structural study of TPI-3Se···l interactions

**Figure 1:**
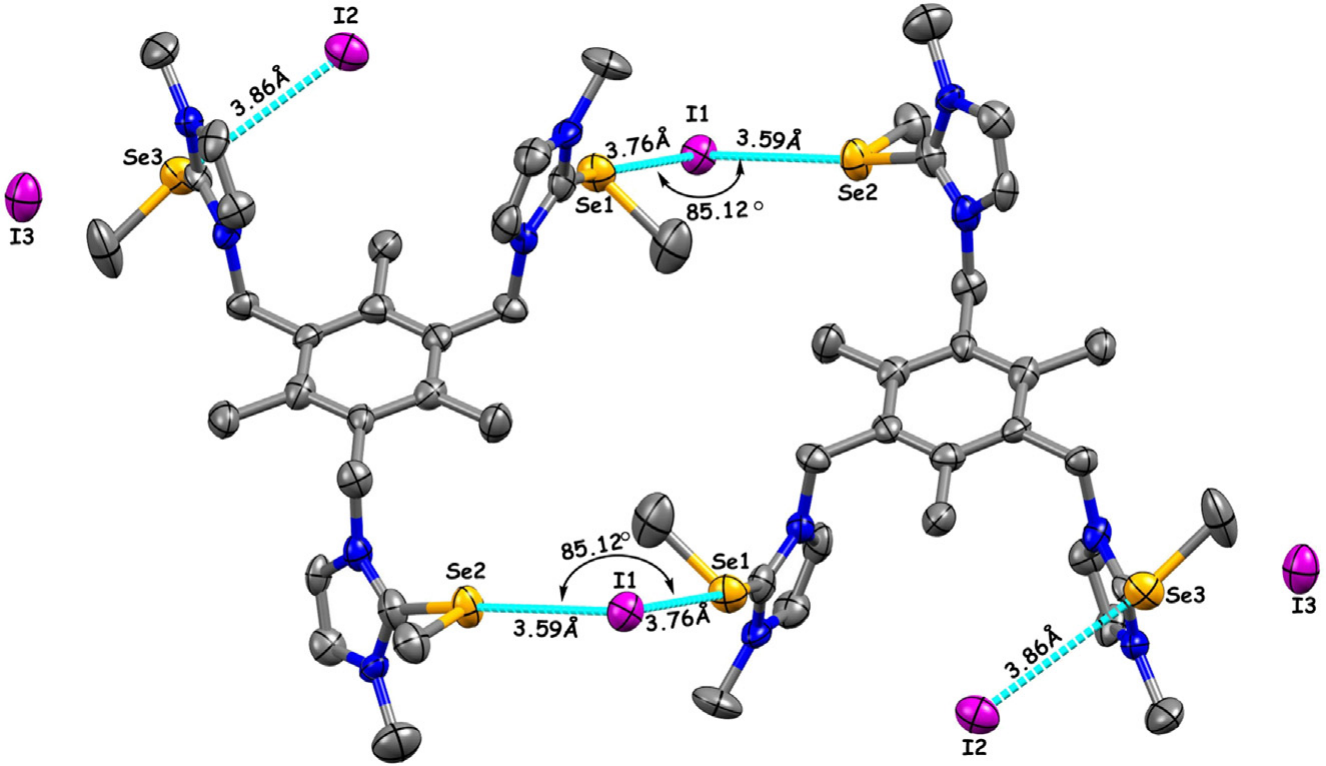
X-ray crystallographic *μ*_*1*_ and *μ*_*2*_ coordinated structures of TPI-3Se–I ChB interaction represented with cyan dash line. Purple = Iodine, blue = Nitrogen, yellow = Selenium. All H atoms are removed for clarity.

Further insight into the host-guest complexation through ChB interactions between I^−^ and tri-cationic host is demonstrated by solid-state structural studies. Monoclinic crystal with *P*21/n space group of the iodide complex TPI-3Se–I (Table S2 and Figure S58) is grown by slow diffusion of diethyl ether and methanol binary solvent. The structure reveals that the three σ-hole donor Se atoms are participating in intermolecular C–Se···I ChB interactions with three iodide anions. One iodide (I2) is coordinated with the σ-hole located on the prolongation of the C_Me_–Se bond (Se3–I2) with *μ*_*1*_ coordination, and another two iodides (I1) are coordinated by the two Se (Se1 and Se2) from the neighboring molecules, providing a *μ*_*2*_ coordination, whereas I3 occluded in the crystal structure as a counter anion for the overall charge neutrality (Figure 1). In the case of *μ*_*2*_ coordination, the ChB interactions happen through the σ-holes present along with the C_Imd_–Se bond. Therefore, in the unit cell, I2 is fully contributed to the one host molecule, but I1 is equally distributed to the adjacent two host molecules through two *μ*_*2*_ coordination, which unambiguously indicates the 1:2 host-guest stoichiometry in the solid state. The ChB distances between Se···I^−^ are found to be 3.59 to 3.86 Å (Table S3) that are shorter than 4.10 Å, the sum of van der Waals radius of Se (1.90 Å) and ionic radius I^−^ (2.20 Å).^70^^,^^71^^,^^72^ The ChB angles (formed with I, Se, and C) are found to be 169.18 to 171.69⁰, i.e., almost linear (Figure S58B and Table S3). These distance and angle parameters clearly suggest the formation of a strong Se-based chalcogen bonded I^−^ complex in solid state through both *μ*_*1*_ and *μ*_*2*_ coordination.^47^^,^^57^ Further, the *μ*_*2*_ coordination with Se···l···Se bond angle of 85.12⁰ resulted a zigzag 1D extended supramolecular network through chalcogen bonding interactions (Figure S59).

In addition, to evaluate the selective I^−^ binding superiority over a mixture of halides (Cl^−^, Br^−^, and I^−^) by the TPI-3Se, we investigate the fate of the solution of 1:1 mixture of TPI-3Se and sodium salt of halides in 100% aqueous medium. From this solution, we have found that TPI-3Se crystalizes out I^−^ selectively through ChB interaction to form TPI-3Se–MI host-guest complex which is confirmed by single-crystal X-ray crystallography. The crystallographic details are given in Table S4 and Figure S60. Therefore, the solution state selectivity studies along with the aforementioned solid-state analysis manifest the superiority of I^−^ toward TPI-3Se in the presence of competitive halide ions.

### Mode of ChB···l interaction by DFT calculation

**Figure 2:**
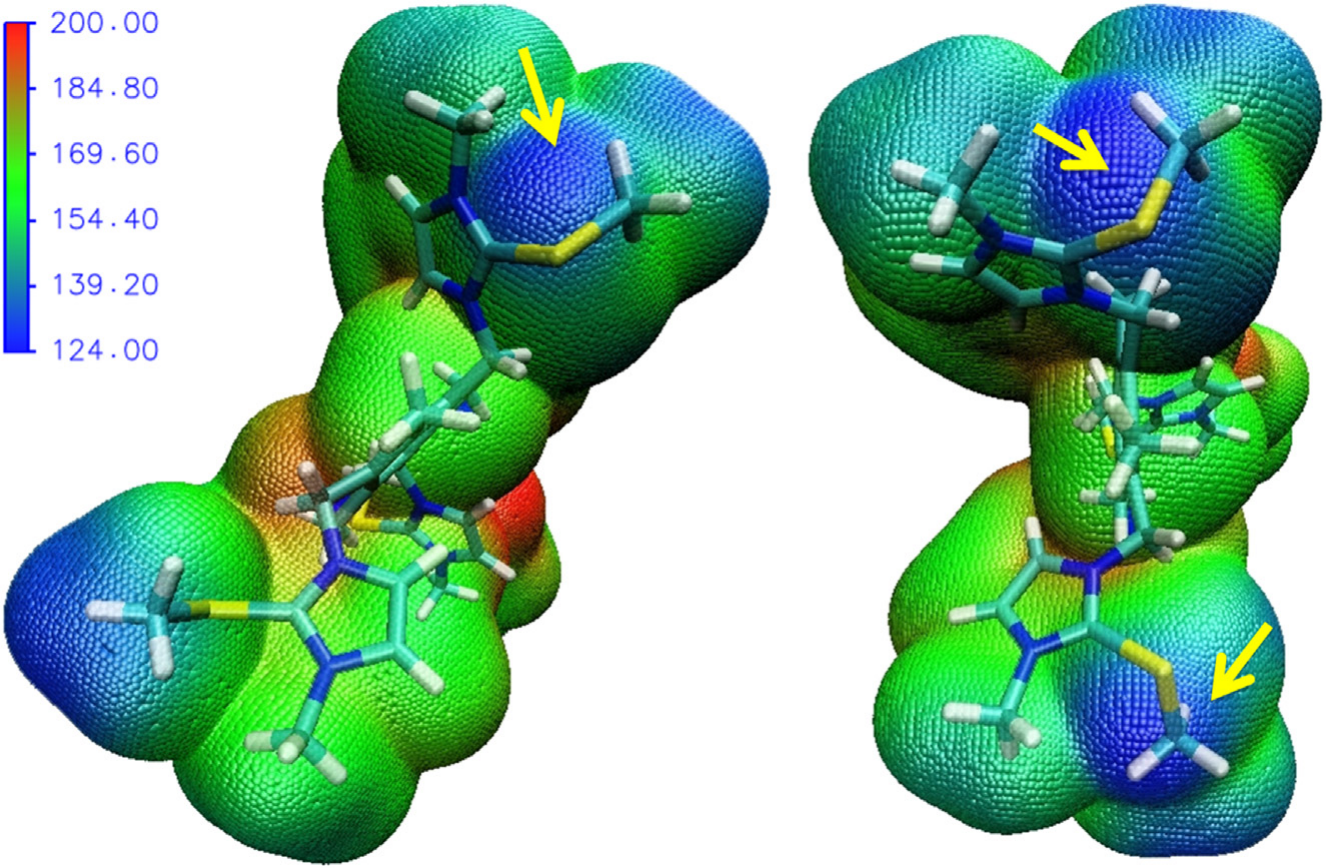
Computed electrostatic potential surface of TPI-3Se; negative charge density (red), and positive charge density (blue) Yellow arrow represents the σ hole. Scale unit: kcal/mol.

Further, DFT calculations of TPI-3Se and its iodide complex (TPI-3Se–I) are performed to correlate the experimental results. The DFT-optimized structures of TPI-3Se and TPI-3Se–I are shown in Figure S61. The optimized geometry of the TPI-3Se–I shows ChB interactions having C—Se···I distances in the range of 2.95–3.04 Å with (C–Se–I) angles ∼180⁰ (Table S5), which represents significant chalcogen bonding interactions.^73^ A weak anion–π interaction is also observed between I78 and imidazolium ring: the shortest distance and angle between the plane of imidazolium system and I^−^ are 3.84 Å and 50.87⁰ respectively (Figure S62).^74^^,^^75^^,^^76^ The single crystal X-ray structure also supports such anion–π interaction (Figure S63). The electrostatic potential calculation of TPI-3Se clearly defines the strong electron-deficient region (σ-holes) on each of the Se atom, whereas one of the σ-holes is centered on C_Imd_–Se and another is on C_Me_–Se bond with large V_s,max_ values (Figure 2 and Table S6). Thus, this structural analysis manifests that there might be an electrostatic attraction between the σ-hole of the Se and electron-rich I^−^, which is comparable with some of the recently reported computational studies.^62^^,^^63^^,^^64^^,^^70^^,^^71^^,^^72^

The nature of non-covalent interactions between iodide and TPI-3Se are further analyzed by the noncovalent interaction-reduced density gradient (NCI-RDG).^77^ The plot of reduced electron density gradient RDG(s) vs. sign(λ_2_)ρ(r) shows three spikes of sign(λ_2_)ρ in the low-density and low-gradient region (Figure S64). The spikes shifted toward a more negative sign(λ_2_)ρ region, suggesting the presence of strong non-covalent interaction between I^−^ and TPI-3Se. It can also be confirmed from the NCI isosurfaces, where the deep blue disc-like embodies are observed due to efficient σ-hole-based ChB interactions between Se and I^−^ (Figure S65).

Additionally, the NBO analysis reveals that the lone pair (LP) electrons of I^−^ transfer to the σ∗ orbital of the ChB donor (C–Se). The extent of the charge transfer from LP (I^−^) into σ∗(C–Se) is represented by a second-order perturbation energy E(2) (Table S7).^78^^,^^79^^,^^80^ The high value of E(2) exhibits the strong σ-hole interaction between Se and I^−^.

### Preliminary iodide extraction study

During ^13^C and ^77^Se-NMR studies of TPI-3Se with I^−^ in D_2_O immediate precipitation of the host-guest adducts was observed which prompted us to extract I^−^ from the aqueous medium through the precipitation method. Therefore, to establish the removal of I^−^ from 100% water medium a typical experiment is carried out by treating 17.6 mM aqueous solution of TPI-3Se with 0.52 M NaI (∼30 equivalents) at pH 7. After that immediate formation of host-guest adducts (TPI-3Se–I) as a white precipitate is collected and characterized by different techniques.

**Figure 3:**
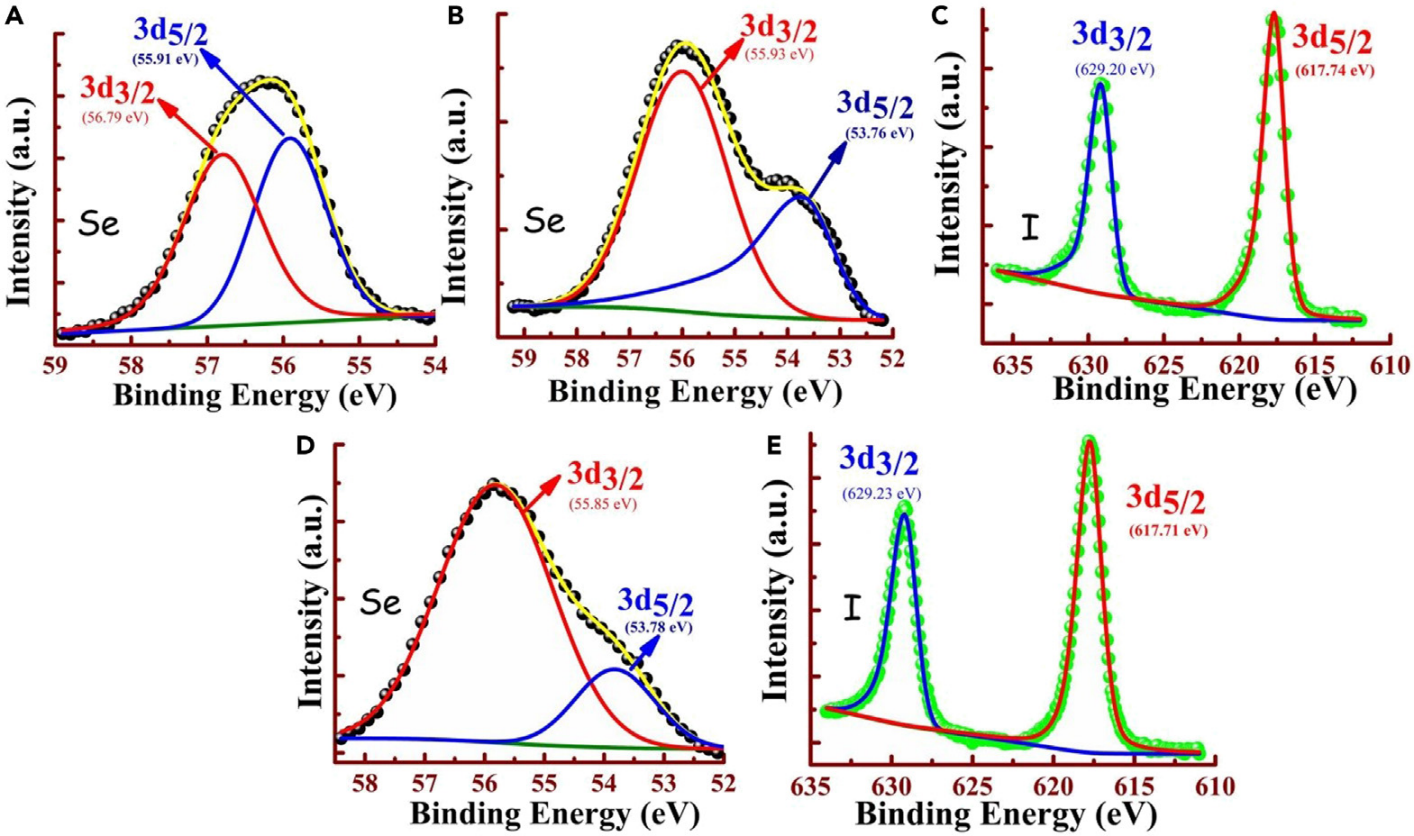
XPS spectra of TPI-3Se before and after uptake of I^−^ (A) Se3d of TPI-3Se (B) Se3d of TPI-3Se–I, (C) I3d of TPI-3Se–I, (D) Se3d of TPI-3Se–MI, and (E) I3d of TPI-3Se–MI.

Firstly, XPS analysis is employed on the precipitated adducts (TPI-3Se–I) to confirm the I^−^ uptake from the solution as well as to determine the binding affinity. In the survey spectrum, two new peaks are located for TPI-3Se–I, which correspond to I3d_3/2_ and I3d_5/2_ with binding energies 617.78 and 629.27 eV, respectively (Figure 3C). Further analysis of Se3d, high-resolution spectra of TPI-3Se and TPI-3Se–I (Figures 3A and 3B) reveal that the binding energies of Se3d_5/2_ and Se3d_3/2_ peaks are shifted from 55.91 to 53.76 eV and 56.79 to 55.93 eV, respectively.^81^^,^^82^ The decrease in the binding energies of Se3d can be attributed to the transfer of charge from I^−^ to Se which eventually increases the electronic repulsion within the Se, as a result, the Se core levels shifted to the lower binding energies.^83^^,^^84^^,^^85^^,^^86^ Therefore, it clearly culminates that I^−^ uptake is occurred by TPI-3Se through the ChB interaction.

**Figure 4:**
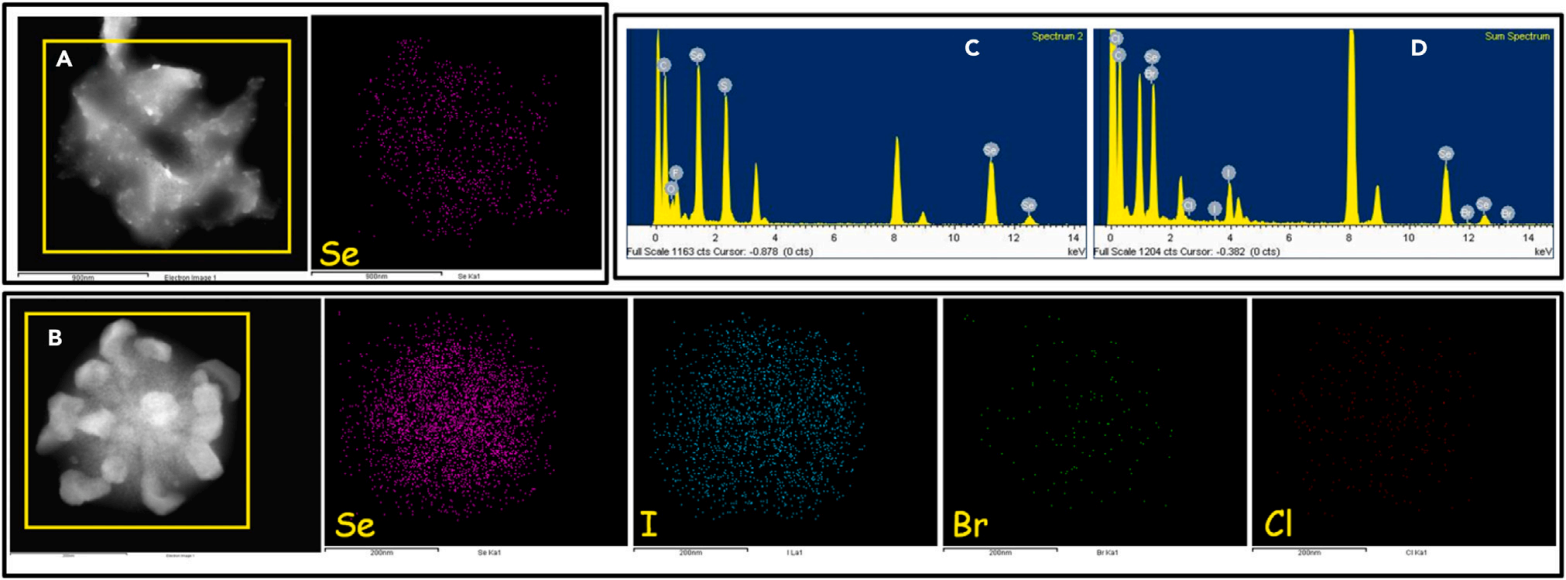
TEM micrographs and EDS element mapping (A) TPI-3Se, (B) TPI-3Se–MI, (C) EDX spectra of TPI-3Se, and (D) EDX spectra of TPI-3Se–MI.

Furthermore, the TEM analysis of TPI-3Se and TPI-3Se–I, is performed to obtain the elemental mapping which clearly demonstrates that iodide is uniformly distributed in the sample of TPI-3Se–I (Figures 4 and S66–S69).

In addition, the FTIR spectrum of both TPI-3Se and TPI-3Se–I, clearly showed that the significant stretching frequency of triflate ion at 1036 cm^−1^ and 641 cm^−1^ almost disappeared in TPI-3Se–I (Figure S70), which is indicative of I^−^ uptake by the TPI-3Se in conjugation with anion exchange mechanism.^87^

Thus, based on the aforementioned studies indeed confirms the extraction of I^−^ from 100% water medium by precipitation through ChB interaction as a complex of TPI-3Se–I.

### Selective iodide extraction from mixed halide ions

We have also investigated the effect of I^−^ uptake by TPI-3Se in the presence of high concentrations of co-existing other halides (Cl^−^ and Br^−^) (I^−^: Cl^−^/Br^−^ = 1:100) in 100% water. Interestingly, it has been found that the iodide complex gets precipitated (TPI-3Se–MI) selectively from the mixed halides (Cl^−^, Br^−^, and I^−^) solution which is confirmed by XPS, TEM-EDX, EDS-mapping, and PXRD data. The extracted solid from the mixed halides solution (TPI-3Se–MI) has also shown the I3d_3/2_, and I3d_5/2_ peaks in the high-resolution XPS spectra with almost the same binding energies compared to TPI-3Se–I, and no peak correspond to Br and Cl are observed (Figure 3E). Not only that, the Se3d_5/2_ and Se3d_3/2_ peaks also show almost same binding energies (53.78 and 55.85 eV respectively) as in TPI-3Se—I (Figure 3D).

Furthermore, to determine the chemical compositions of TPI-3Se–MI, energy dispersive X-ray (EDX) measurements are performed. The results indicate only the presence of C, Se, and I in both TPI-3Se–I and TPI-3Se–MI samples (Figures 4, S68, S69, S71, and S72). Meanwhile, the EDS mapping images corresponding to the area in Figure 4 conclusively revealed the uniform distribution of them. In contrast, significantly negligible bromide or chloride sample distribution is observed in TPI-3Se–MI, which further supports the observation of selective I^−^ uptake by the ChB donor TPI-3Se.

Additionally, the FTIR spectrometric analyses of TPI-3Se–MI indicated no obvious spectral changes *w.r.t* TPI-3Se–I (Figure S70), which also confirms the appeal interpretation.

Besides, Cl^−^ and Br^−^, we have also precipitated the ChB-anion complex in the presence of a mixture of sodium salts (I^−^: Anions = 1:50) in 100% water (anion salts = NaF, NaCl, NaBr, NaI, NaH_2_PO_4_, NaCN, and NaAcO). The TEM EDX analysis of the precipitated ChB-anion complex clearly shows that only a significant amount of Se and I, have been distributed in the sample, rather than F, Cl, Br, P, and O (Figures S73 and S74). In addition to the absence of stretching frequencies for -CN, and C=O (AcO^−^) in the IR spectra, which manifest that ChB receptor shows fondness selectively to form ChB-anion complex with I^−^ only (Figure S75).

Therefore, all these studies clearly suggest that the TPI-3Se selectively binds and extracts the I^−^ from the pure water medium even in the presence of various sodium salt anions as well as high concentration of Br^−^ and Cl^−^.

**Figure 5:**
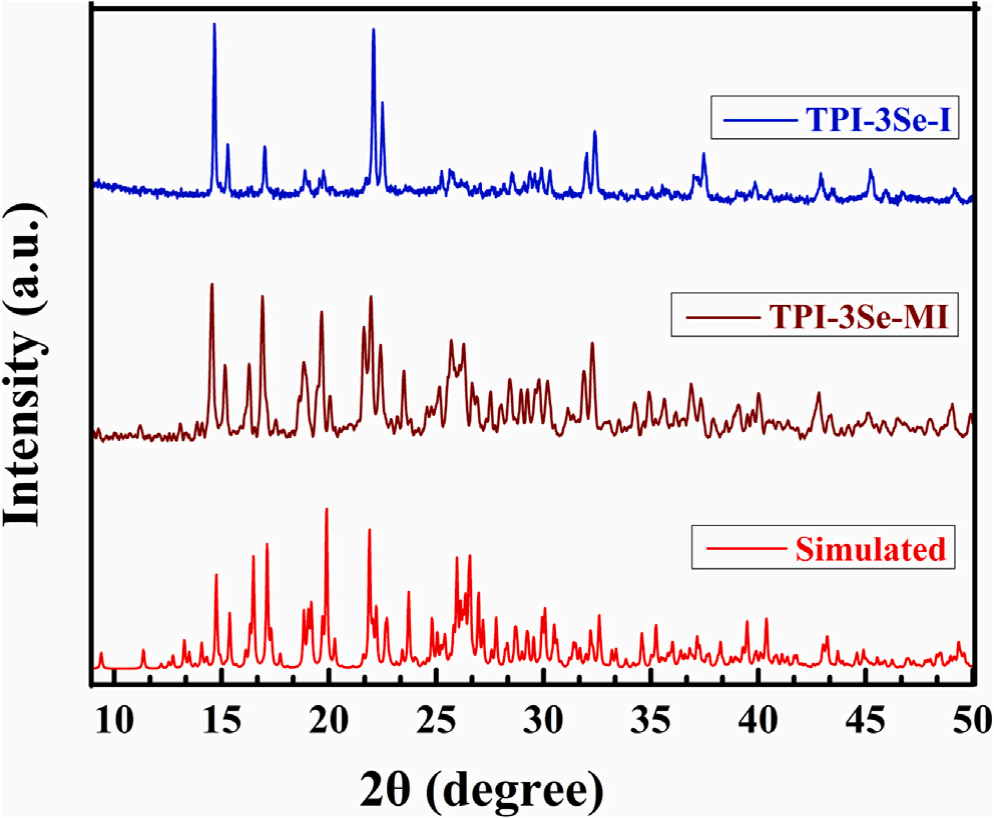
PXRD spectra of the iodide complexes and the simulated pattern

In order to check the bulk purity of the extracted mass, we have further investigated the powder diffraction pattern of the extracted iodide complexes (TPI-3Se–I and TPI-3Se–MI) and have compared it with the simulated pattern. The resemblance of the experimental PXRD patterns with the simulated one indicated that the precipitate iodide complexes form with a high degree of bulk purity (Figure 5). Thus, we can endorse that the ChB receptor TPI-3Se can be used for pure extraction of I^−^ from 100% water medium in the presence of competitive halides.

Furthermore, in order to generalize the superiority of ChB receptor TPI-3Se over their HB (TPI-3H) and XB analogs (TPI-3I), we have investigated the selective precipitation of the halides complex. Eventually, the precipitated halide complexes of TPI-3H and TPI-3I demonstrate all the halides (I^−^, Cl^−^, and Br^−^) are concurrently precipitated, which is confirmed by the TEM-EDX and mapping data (Figures S76–S79). Therefore, it can conclusively reveal that only TPI-3Se is selectively able to remove I^−^ but its analogs TPI-3H and TPI-3I fail to do so.

### Extraction of iodide under batch experiments

To evaluate the removal efficiency of I^−^ through the TPI-3Se, concentration-dependent extraction experiments are carried out. Therefore, we have performed batch experiments with different concentrations of I^−^ which clearly revealed that the minimum ∼0.3 M I^−^ concentration is required to precipitate the ChB-iodide host-guest complex. The detailed removal of I^−^ procedures is discussed in the ESI. To determine, the remaining I^−^ concentration in the solution UV-Vis spectroscopy is used.^88^ It is important to note that TPI-3Se shows maximum 70.4% extraction efficiency when the minimum concentration of I^−^ becomes ∼0.5 M (Table 3). For the practical applicability, the pH-dependent (pH = 3, 7, and 10) I^−^ removal efficiency is also evaluated and the results demonstrated there is no significant effect on efficiency by varying the pH of the solution (Table S8). In all the cases, the PXRD pattern of the precipitated ChB—iodide complexes along with TEM-EDX data have shown similar to that of the pure TPI-3Se-I complex (Figures 4 and S80–S85). This result manifests that in 100% aqueous medium pure I^−^ extraction can be achieved by the ChB receptor TPI-3Se and also illustrates the exploration of the ChB bond as a functional material for real-world application.

**Table 3:** I^−^ extraction efficiency of TPI-3Se at different concentrations of I^−^

Initial concentration of I^−^ (M)	After extraction remaining concentration of I^−^ (M)	Extraction efficiency for TPI-3Se (%)
0.32	0.099 (2.5)	69.06 (2.2)
0.41	0.124 (1.9)	69.80 (2.6)
0.52	0.154 (2.1)	70.40 (1.8)
0.81	0.245 (2.5)	69.75 (2.2)
1.2	0.362 (1.7)	69.83 (2.5)

### Conclusions

In summary, this work demonstrates the first selenium-based tripodal ChB receptor which selectively extracts iodide from 100% water through the σ-hole-based interactions. Thus, it highlights the genuine potential of a sigma-hole-based receptor for anion extraction from water. The integration of ChB donor motifs into hosts of different dimensionalities is currently underway in our laboratory for selective extraction and catalysis studies.

### Limitations of the study

It is worth noting that this work has revealed valuable insights into the ChB chemistry for the removal of iodide from water medium. However, the observed removal capacity by the ChB receptor is dependent on concentration of iodide in the molar range. Despite this, the most intriguing is that in the solid state structure, iodide can bind to the ChB receptor, simultaneously through both *μ*_*1*_ and *μ*_2_ coordination modes. Hence, elucidating these mechanisms will further lead to new advancements to improve the extraction efficiency at the ppm level by integrating the ChB sigma-hole in the receptors as well as open a new area in fine-tuning opportunity for anion selectivity and affinity.

## STAR★Methods

### Key resources table

**Table:** 

REAGENT or RESOURCE	SOURCE	IDENTIFIER
**Chemicals, peptides, and recombinant proteins**		

All chemicals	Sigma-Aldrich	Can be found from Sigma webpage: https://www.sigmaaldrich.com/IN/en?gclid=CjwKCAjwrranBhAEEiwAzbhNtcdAAWpwRLra4P3fSnz757Q7-RKl7DP3hQcwmyhtsLvE_iTx3rhtmxoC9LAQAvD_BwE

**Software and algorithms**		

ChemDraw Professional 20.0	PerkinElmer	https://www.perkinelmer.com/category/chemdraw
CasaXPS: Processing Software for XPS	Casa Software Ltd	http://www.casaxps.com/
Multiwfn	Chen et al.^89^	http://sobereva.com/multiwfn/
VMD	Schulten et al.^90^	https://www.ks.uiuc.edu/Research/vmd/
Gaussian09	Frish et al.^91^	https://gaussian.com
Graph plotting	Origin Lab	Origin: Data Analysis and Graphing Software (originlab.com)

### Resource availability

#### Lead contact

Further information and requests for resources should be directed to and will be fulfilled by the lead contact, Pradyut Ghosh (icpg@iacs.res.in).

#### Materials availability

The material will be provided following the request to the lead contact following the proper channel. For rest see key resources table in STAR Methods.

#### Data and code availability

• Full X-ray crystallography structural factor files are available free of charge from the CCDC via https://www.ccdc.cam.ac.uk/ CCDC 2180385 (for compound TPI-3Se-I) and CCDC 2217469 (for compound TPI-3Se-MI). All synthesis and characterization data of compounds, All NMR titration data, and DFT calculation results are included in Supplemental Experimental Procedures.
• This paper does not report original code.
• Any additional information required to reanalyze the data reported in this paper are available from the lead contact upon request.

### Experimental model and subject details

Our study does not use experimental models.

### Method details

#### Experimental procedures and characterisation

##### Preparation and characterization of P1

To the solution of mesitylene (1.0g, 8.33 mmol) and para-formaldehyde (0.77g, 25.82 mmol) in glacial acetic acid(5 ml), a glacial acetic acid solution of HBr (8ml, 31wt%) was added dropwise and left for heating at 95°C for 12 h. Afterwards, the suspension was poured into a beaker containing 50 ml of distilled water and the obtained solid was filtered with subsequent washing with water, and dried in air (93 % yield). ^1^H NMR (CDCl_3_, 298K, 300 MHz): δ (ppm) 4.58 (s, 6H), 2.47 (s, 9H).(Scheme S1 and Figure S1).

##### Preparation and characterization of P2

An acetonitrile (40mL) solution of 1,3,5-tris(bromomethyl)-2,4,6-trimethylbenzene (1 equiv.,1.85 mmol) was introduced with 1-methyl imidazole (3.3 equiv., 5.9 mmol) under argon atmosphere with reflux condition for 24hr. A thick white precipitate was started to form indicating the product formation. Afterward, the white solid was filtered off and washed by acetonitrile to remove excess 1-methyl imidazole along with subsequent washing with diethyl ether for several times (Yield 84%). ^1^H NMR (300 MHz, 298K, DMSO-d_6_): δ(ppm) 9.04(3H, s), 7.75(t, 3H, J = 1.83 Hz), 7.72(t, 3H, J = 1.83 Hz), 5.54(6H, s), 3.86(9H, s), 2.30(9H, s). (Scheme S1 and Figure S2).

##### Preparation and characterization of P3

Under an argon atmosphere, compound P2 (1 equiv.,0.69 mmol), K_2_CO_3_ (6 equiv.,4.14 mmol) and elemental selenium powder (6 equiv., 4.14 mmol) were taken into a two-necked R.B containing dry Methanol(25mL). The solution was left for reflux for 24 hr. with vigorously stirring. Next the hot solution was filtered by vaccum filtration through celite and washed by DCM for two times. Finally, by evaporating the solvent desired product was obtained (90% yield). ^1^H NMR (300 MHz, 298K, CDCl_3_): δ(ppm) 6.79(d, 3H, J = 2.37 Hz), 6.23(d, 3H, J = 2.34 Hz), 5.28(6H, s), 3.73(9H, s), 2.21(9H, s). (Scheme S1 and Figure S3).

##### Preparation and characterization of TPI-3Se

The targeted compound TPI-3Se was synthesized by methylation of the 3,3',3''-((2,4,6-trimethylbenzene-1,3,5-triyl)tris(methylene))tris(1-methyl-1,3-dihydro-2H-imidazole-2-selenone) (P3). Compound P3 (1 equiv.,0.378 mmol) was taken into a two-necked R.B under an argon atmosphere, 20 mL of dry DCM was added into it while the temperature was kept at 0ᵒC. At this temperature methyl triflate (6 equiv.,2.27 mmol) was added dropwise to the DCM solution. Then the solution was stirring at 0ᵒC for 2 hrs. and after that the stirring was continued at room temperature for overnight. After that, DCM was decanted out and the solid was washed by sonication with diethyl ether for several times to obtained the methylated solid product (85 % yield). ^1^H NMR (500 MHz, 298K DMSO-d_6_): δ(ppm) 7.92 (d, 3H, J = 2.75 Hz), 7.23(d, 3H, J = 2.35 Hz), 5.50(6H, s), 3.94(9H, s), 2.45(9H, s), 2.21(9H, s). ^13^C NMR (75 MHz, DMSO-d_6_): δ(ppm) 141.33, 136.78, 129.33, 125.58, 122.81, 122.37,118.54, 49.12, 37.25, 16.39, 9.69. ^13^C-DEPT-135 NMR: 126.15 (CH), 122.91(CH), 49.69(CH_2_), 37.87(CH_3_),16.97(CH_3_) 10.35(CH_3_). ESI-MS: m/z: calcd. for C_29_H_39_F_6_N_6_O_6_S_2_Se_3_ [L.2OTf]^+^ 982.97, found 982.99. (Scheme S1 and Figures S4–S11).

##### Preparation and characterization of TPI-3H

An acetonitrile (40mL) solution of 1,3,5-tris(bromomethyl)-2,4,6-trimethylbenzene (1 equiv.,1.85 mmol) was introduced with 1-methyl imidazole (3.3 equiv., 5.9 mmol) under argon atmosphere with reflux condition. A thick precipitate was started to form and the reaction was continued till 1day. Afterward, the white solid was filtered off and washed by acetonitrile to remove excess 1-methyl imidazole and finally washed with diethyl ether for several times. The obtained bromide salt was dissolved in water and poured into a AgOTf solution of water resulting in thick white precipitate of AgBr. After that, the remaining solution was filtered off and evaporated to dryness (93 % yield). ^1^H NMR (300 MHz, 298K, DMSO-d_6_): δ(ppm) 8.75(3H, s), 7.73(3H, s), 7.62(d, 3H, J = 1.62 Hz), 5.53(6H, s), 3.80(9H, s), 2.30(9H, s); ^13^C NMR (75 MHz, 298K, DMSO-d_6_): δ(ppm) 141.06, 136.06, 129.39, 123.83, 122.16, 47.61, 35.89, 16.20. (Figures S12–S14).

##### Preparation and characterization of TPI-3I

An acetonitrile (40mL) solution of 1,3,5-tris(bromomethyl)-2,4,6-trimethylbenzene (1 equiv.) was introduced with 1-methyl-2-iodo imidazole (3.3 equiv.) under argon atmosphere with reflux condition. Precipitate was started to form and the reaction was continued till 24hr. Afterward, the yellow solid was filtered off and washed by acetonitrile to remove excess 1-methyl-2-iodo imidazole and finally washed with diethyl ether for several times. The dried mass was dissolved in water and poured into a AgOTf solution of water resulting in thick white precipitate of AgBr. After that, the remaining solution was filtered off and evaporated to dryness (82 % yield). ^1^H NMR (300 MHz, 298K, DMSO-d_6_): δ(ppm) 7.98(3H, s, J = 2.01 Hz), 7.24(s, 3H, J = 2.1 Hz), 5.34(6H, s), 3.86(9H, s), 2.16(9H, s); 13C NMR (75 MHz, 298K, DMSO-d_6_): δ(ppm) 141.50, 128.93, 126.41, 123.27, 122.81, 118.54, 114.26, 102.32, 51.04, 16.43. (Figures S15–S17).

##### NMR titration studies

^1^H-NMR titrations of TPI-3Se with the sodium salts of guest anions were carried out in a 300 MHz NMR instrument at 298K. The measured amount of receptor was taken in the NMR tube and dissolved in 0.5 ml of D_2_O. Known volumes of anionic guest as their sodium salts in D_2_O were added and the spectra were recorded after each addition. In each case, Se-Me proton and one imidazole proton signal of the host was monitored. While for TPI-3H and TPI-3I, titration experiments could not be performed in pure D_2_O due to the precipitation problem. Instead, a binary 1:1 D_2_O and CD_3_CN solvent mixture was used in these cases. For TPI-3H and TPI-3I, acidic proton in the azolium ring and imidazole proton signal of the receptors were monitored respectively. The change in the chemical shift value for the host spectra were monitored as a function of guest concentration. The data was analysed to get the binding constant values from Bindfit software. (Figures S18–S47 and Table S1).

^13^C NMR titrations with TPI-3Se were appeared to be difficult in recording in D_2_O because of the precipitation problem (after addition of 1equiv. NaI). Thus, we choose a 1:1 mixture of D_2_O and CD_3_CN as our experimental binary solvent for this titration experiment. Here also our host was dissolved in 0.5ml mixed solvent and known volumes of sodium halide guest in the same mixed solvent were added and the spectra were recorded. Here Se-Me carbon signal of the host was monitored. (Figures S48–S50).

^77^Se NMR titrations were also done using 1:1 mixture of D_2_O and CD_3_CN. In this case dimethyl diselenide (Me_2_Se_2_) used as an external standard and the spectra of the host were recorded in the absence and presence of 8 equiv. amount of guest solution. The Se-peak corresponds to the host was monitored. (Figures S51–S54).

##### Isothermal titration calorimetric (ITC) studies

In a typical ITC experiment, a 1mM host TPI-3Se solution was titrated with 20mM of sodium salt of halides at 298 K. A 1:1 binary solution of acetonitrile and water(milli-Q) was used as solvent for ITC experiment. (Figures S55–S57).

##### I^—^ extraction studies

The extraction experiments were conducted through batch conditions at pH 3, 7, and 10 where NaI used as the iodide source. The pH value of the solutions was adjusted by using NaOH and HNO_3_ solution. For each pH, 2 mL iodide solution in water with the concentration ∼ 0.3, 0.4, 0.5, 0.8, and 1.0 (M) were prepared and 30mg of TPI-3Se were added into each of them. All the solutions were stirred for 12 hours at room temperature. After-ward, the precipitated solid and the solution were separated using 0.45 μm nylon membrane filter. Then 5μl of each filtrate solutions was diluted in 10ml of distilled water, and 100μl of those solutions was taken in 2ml of distilled water and absorbance was measured by ultraviolet Spectro-photometer (UV–vis) at 227 nm. Finally, the removal capacity was calculated according to the following equation:

$$Removal\;efficiency=\frac{C_{initial}-C_{final}}{C_{initial}}\times 100$$

Where, *C*_initial_ is the initial concentration of I^–^ solution (M), *C*_final_ is the concentration of I^–^ solution after extraction.

Additionally, PXRD and TEM analysis was performed with the extracted mass. (Figures S66–S85 and Table S8).

#### Instrumentation

##### Isothermal Titration Calorimetric (ITC) studies

The thermodynamics of anion binding by TPI-3Se were analyzed by ITC experiments using Microcal VP-ITC.

##### Transmission electron microscope (TEM)

This microscopic analysis was done by JEOL JEM-2100F electron microscope. Energy-dispersive spectroscopic (EDS) measurements to investigate the chemical composition of the structure were also carried out on the same instrument. Samples were prepared on a carbon coated copper grid (300 mesh) by drop casting method where a drop of very dilute solution of the samples in CH_3_CN were placed onto the grid and were dried very well by vacuum desiccators before analysis.

##### Nuclear Magnetic Resonance spectroscopy (NMR)

NMR spectra for both characterisation as well as titration experiment were recorded on FT-NMR Bruker DPX 300 MHz NMR spectrometer (FT-NMR Bruker DPX). Chemical shift values were measured in parts per million (ppm), with reference to the residual solvent peaks (^1^H and ^13^C) or the external standard (dimethyl diselenide) for ^77^Se and were analyzed with MestReNova.

##### Infrared spectroscopy

The FT-IR spectra was recorded on a FTIR-8400S IR spectrophotometer (SHIMADZU) at room temperature and the data analysis was done by Origin software.

##### Uv-Vis spectroscopy

The concentration of iodide in the extraction process were measured by Agilent carry 5000 UV-VIS NIR spectrophotometer.

##### Powder X-ray diffraction

PXRDs were recorded using a Bruker D8 Advance instrument and the data analysis was done by Origin software.

##### X-ray photoelectron spectroscopy (XPS)

XPS analysis were performed using commercial Omicron spectrometer (model 1712-62-11). An Al Kα (1486.7 eV) non-monochromatic x-ray source running at 150 W (15 kV and 10 mA) was used to acquire the data at ambient temperature.

##### High-Resolution ESI-MS

Waters QtoF Model YA 263 instrument in positive mode was used for the ESI-MS.

##### X-ray single crystal

Bruker D8 Venture Microfocus diffractometer with MoKα (λ = 0.71073 Å) radiation (equipped with a PHOTON II detector) was used to collect the crystal data. The data collection was managed by the APEX3(v2017.3-0) software and processed through SAINT.^92^ Furthermore, utilizing the SADABS program, the collected reflections were subjected to empirical absorption correction.^93^ To solved the structures, SHELXTL^94^ and SHELXL-2014^95^ program package were employed. Graphics were generated using PLATON-97^96^ and MERCURY 3.7.^97^

##### Computational Methods

Geometry optimization of TPI-3Se and TPI-3Se-I were carried out choosing higher performance M06-2X function which is mainly taken for noncovalent interactions systems. For the H, C and N atoms, 6-311++G∗∗ basis set was choosen, while for Se and I, the aug-ccpVDZ-PP basis set was taken from the EMSL database.^98^^,^^99^ The electrostatic potential values, including the minima(Vs,min) and maxima (Vs,max) on the TPI-3Se surfaces, were assessed by Multiwfn,^89^ and V(r) was assessed on the 0.001 electrons Bohr-3 contour of ρ(r), while ESP mapping were generated from VMD.^90^ By combining the Multiwfn results with VMD, the corresponding RDG-based NCI spike were generated.^90^ The NBO calculation was also perform to analyse the noncovalent interactions.^100^ Further, the strength of noncovalent interactions was evaluated by calculating the second-order perturbation stabilization energy, E(2). Therefore, for chalcogen bonding, NBO-based analysis was performed on the structures at the M06-2X/6-311++G∗∗/aug-ccpVDZ-PP level using the NBO program implemented in the Gaussian 09 package.^91^

## Supplemental information

Document S1. Figures S1–S85, Scheme S1, and Table S1–S8
