# Supplementary material for: Selective recognition and extraction of iodide from pure water by a tripodal selenoimidazol(ium)-based chalcogen bonding receptor
Source: iScience. 2024 Jan 15;27(2):108917. doi: 10.1016/j.isci.2024.108917 (PMC10847689; doi:10.1016/j.isci.2024.108917)
Supplement: Document S1. Figures S1–S85, Scheme S1, and Table S1–S8 [file mmc1.pdf]

iScience, Volume *27*

Supplemental information

Selective recognition and extraction of iodide from pure water by a tripodal selenoimidazol(ium)-based chalcogen bonding receptor

Abu S.M. Islam, Sourav Pramanik, Sahidul Mondal, Rajib Ghosh, and Pradyut Ghosh

Chalcogen bond donor TPI-3Se:

(i) Synthetic route:

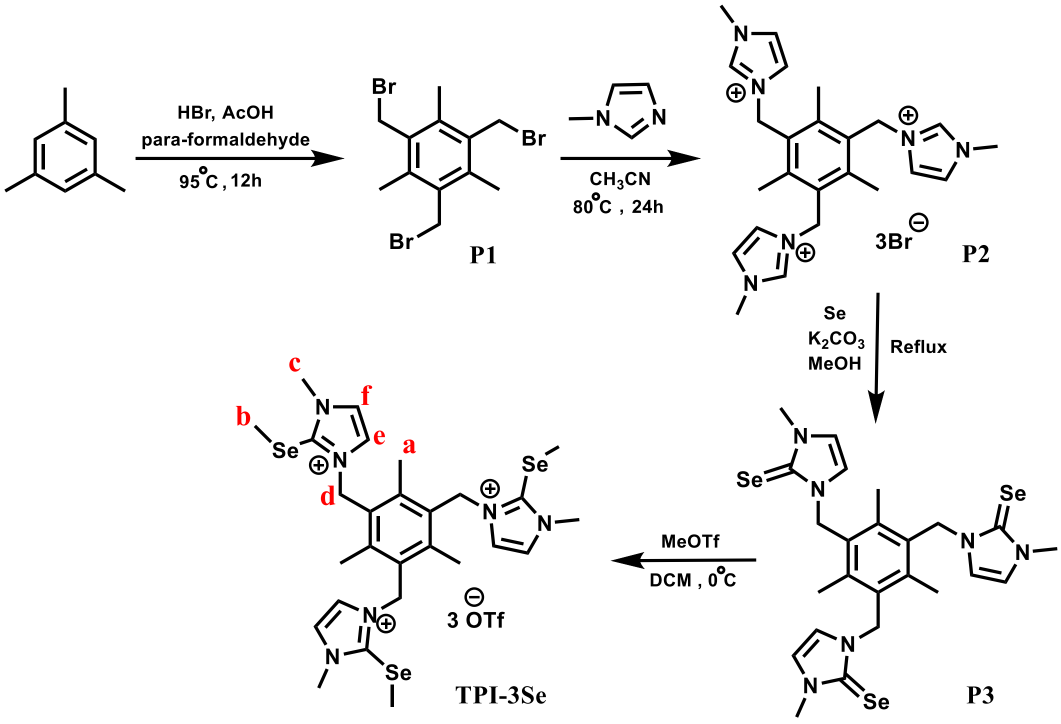

Scheme S1: Synthetic route for the preparation of **TPI-3Se**, related to STAR Methods.

(ii) NMR and Mass spectra:

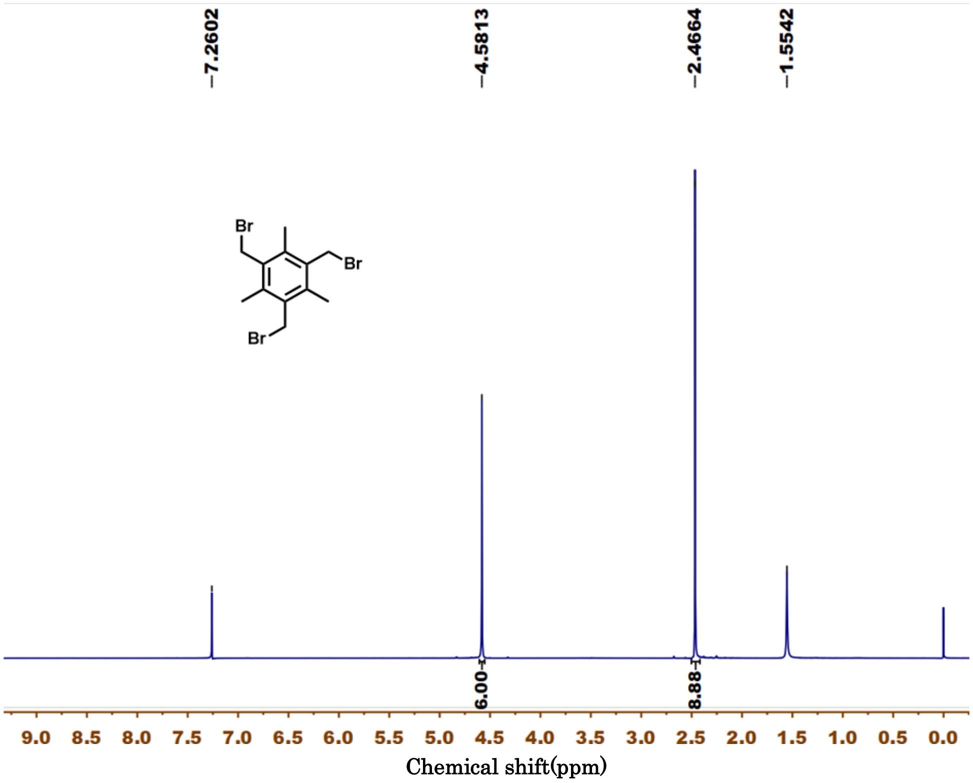

Figure S1. ^{1}H NMR spectrum of **P1** (300 MHz, $CDCl_3$, 298K), related to STAR Methods.

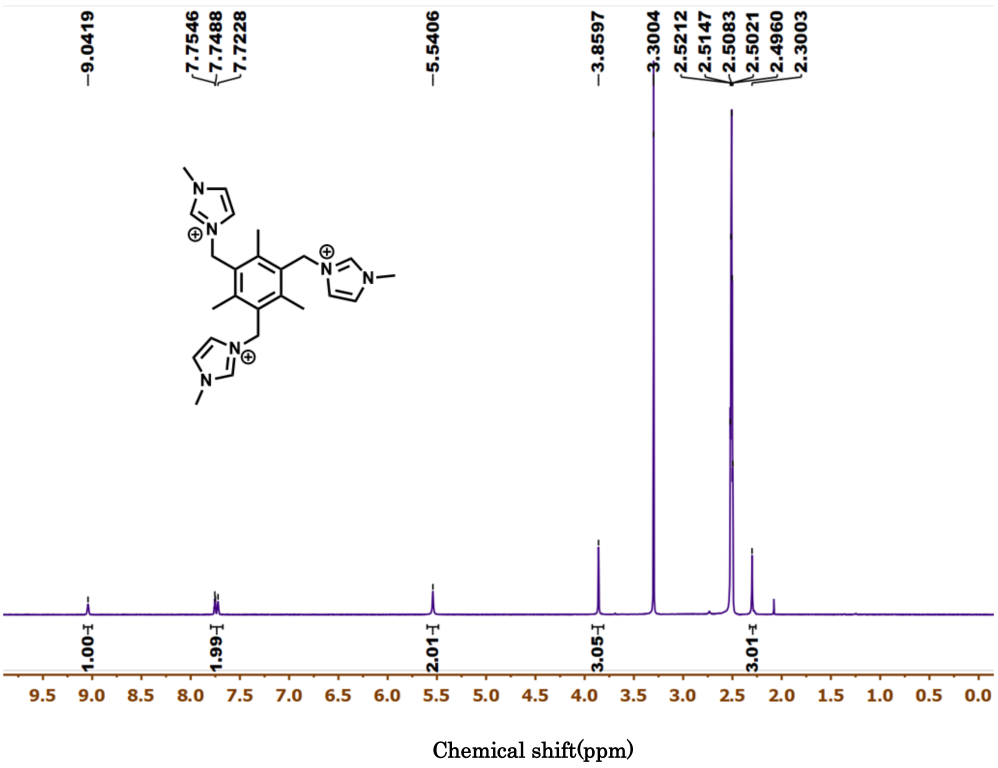

Figure S2. ^1H NMR spectrum of **P2** (300 MHz, DMSO-d_6, 298K), related to STAR Methods.

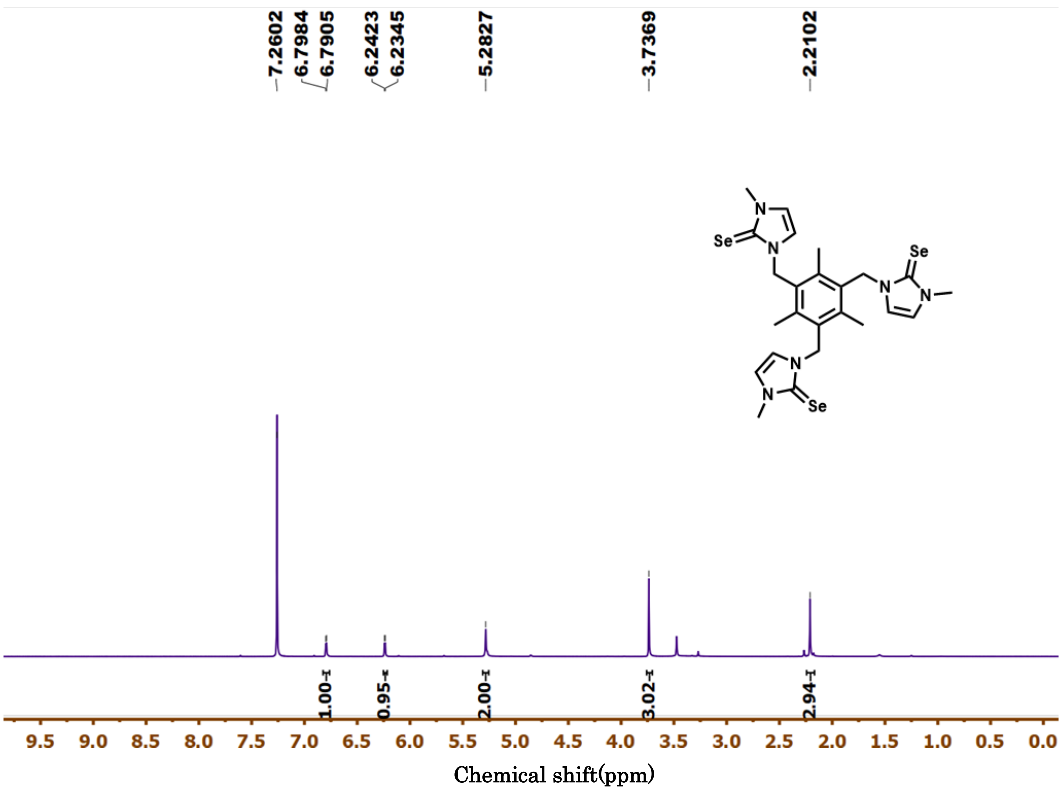

Figure S3. ^1H NMR spectrum of **P3** (300 MHz, $CDCl_3$, 298K), related to STAR Methods.

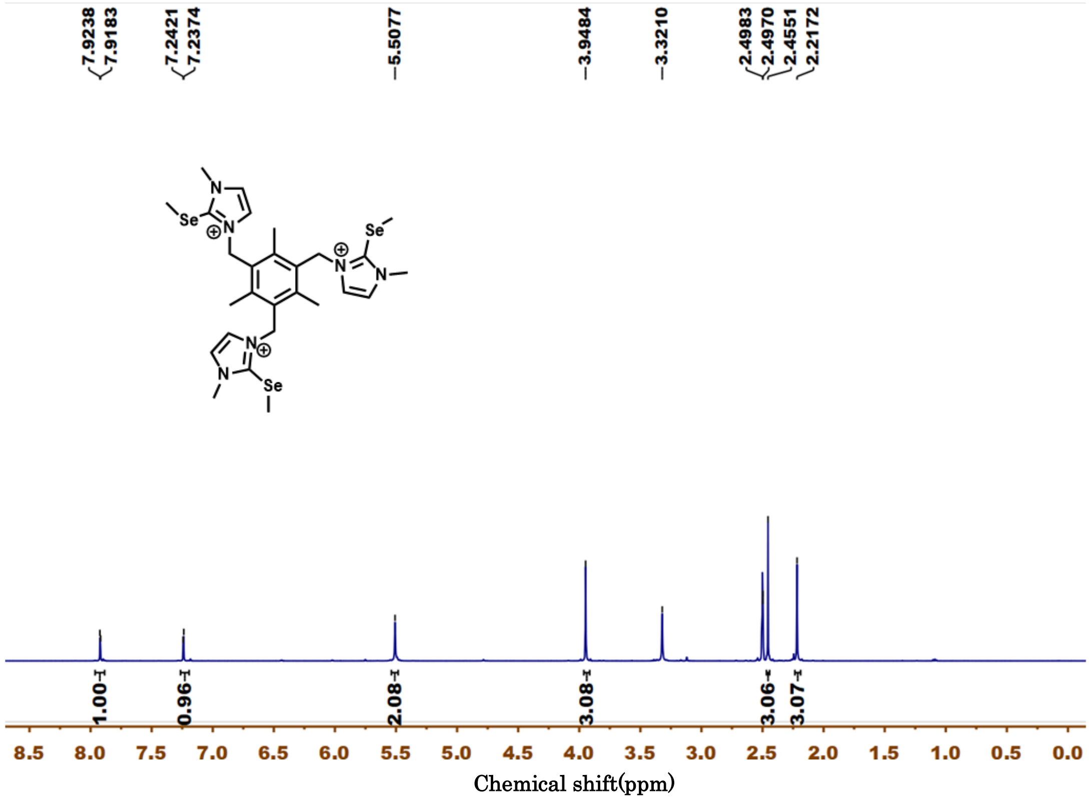

Figure S4. ^{1}H NMR spectrum of **TPI-3Se** (300 MHz, DMSO-d$_6$, 298K), related to STAR Methods.

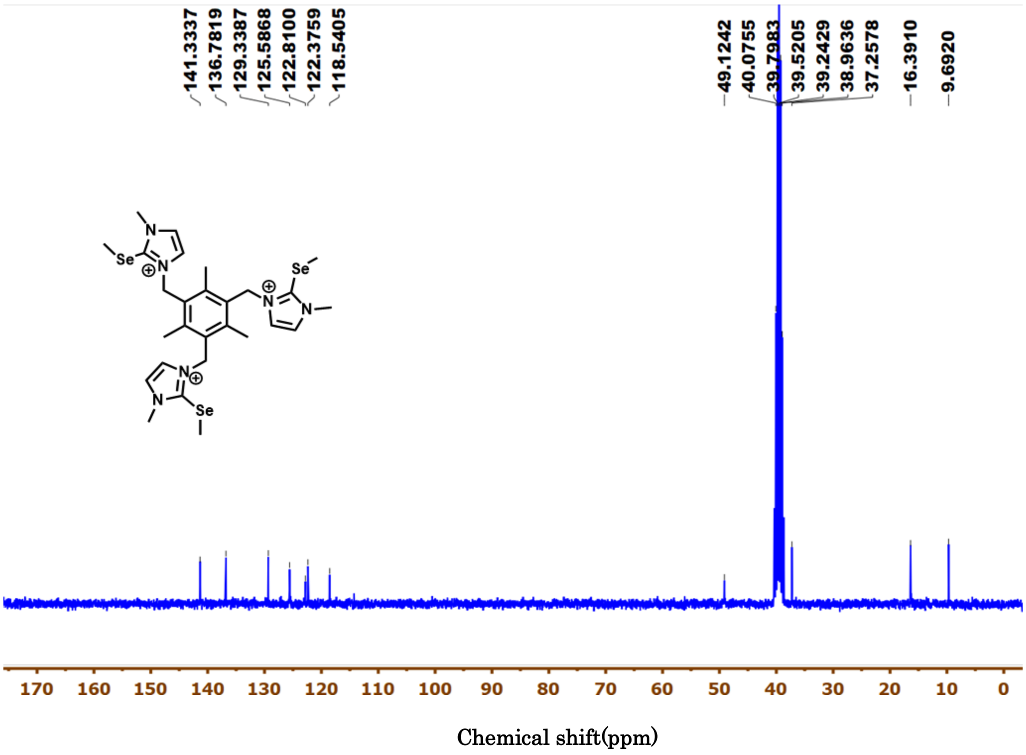

Figure S5. ^{13}C NMR spectrum of **TPI-3Se** (75 MHz, DMSO-d$_6$, 298K), related to STAR Methods.

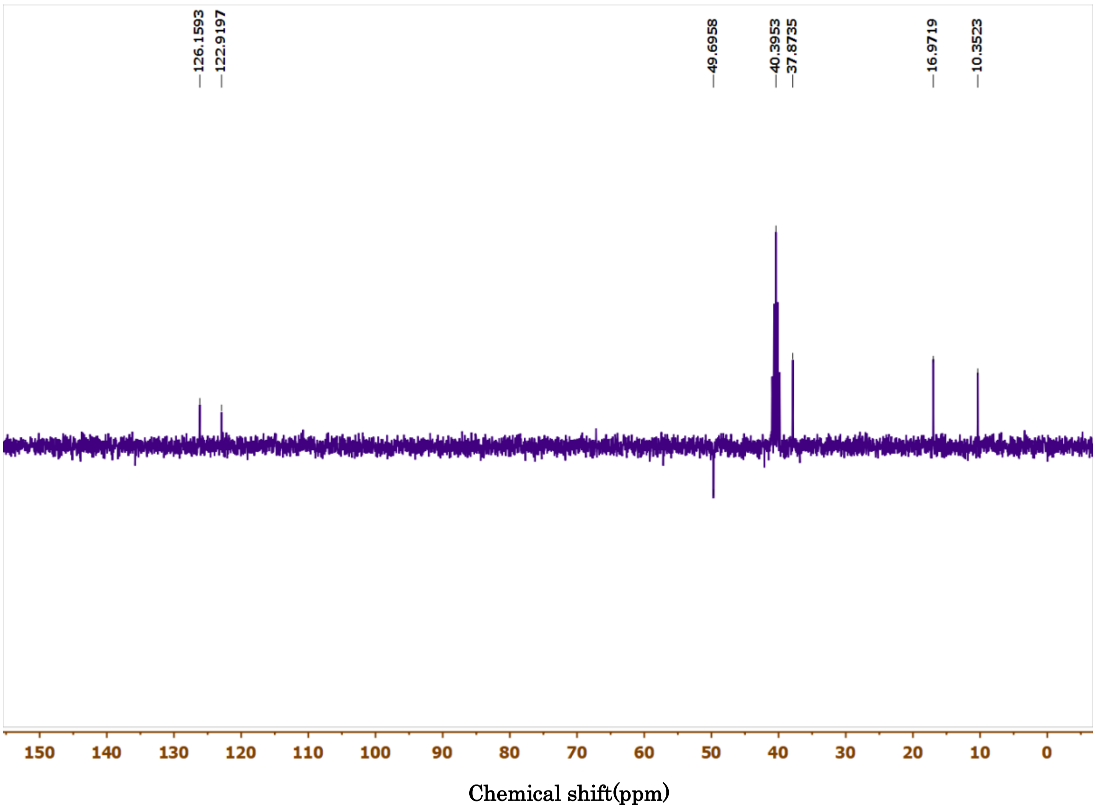

Figure S6. ^{13}C DEPT spectrum of **TPI-3Se** (75 MHz, DMSO-d_6, 298K), related to STAR Methods.

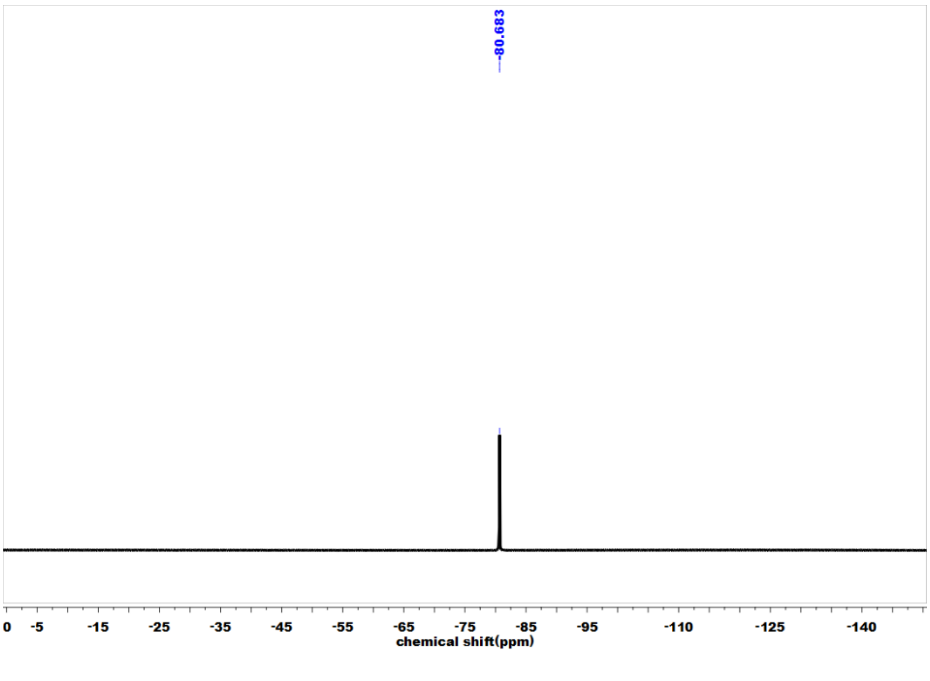

Figure S7. ^{19}F NMR spectrum of **TPI-3Se** (400 MHz, DMSO-d_6, 298K), related to STAR Methods.

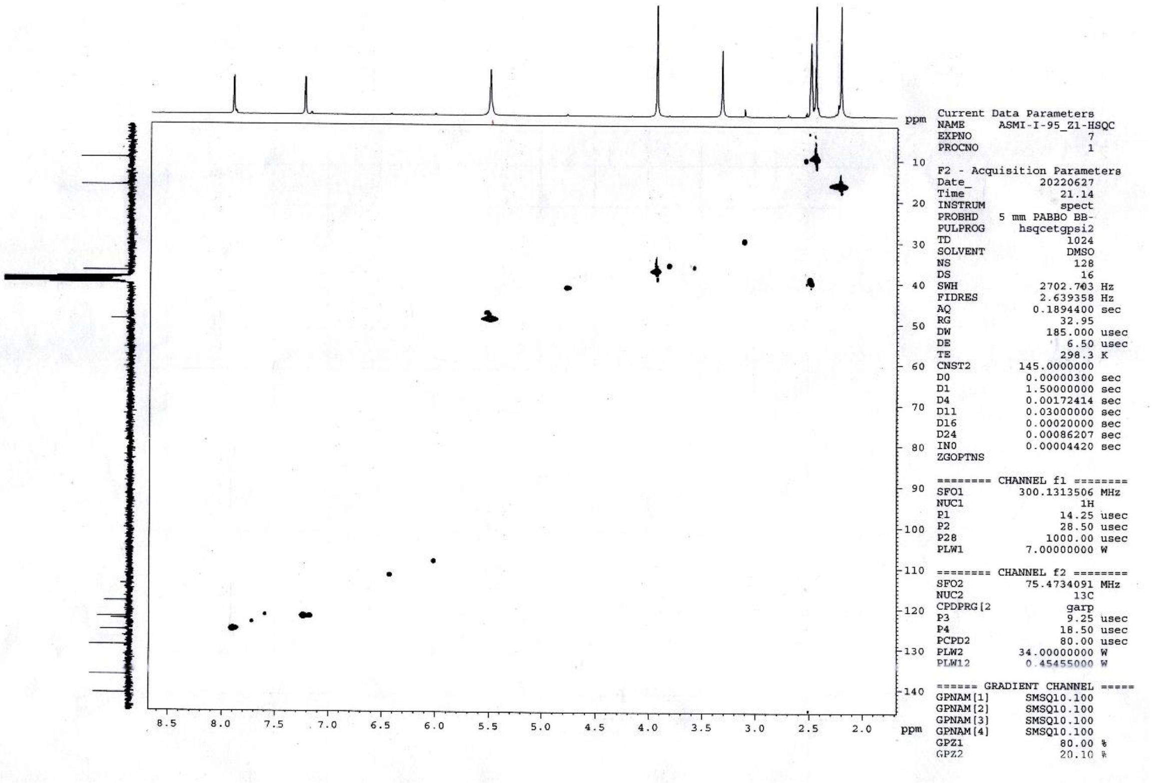

Figure S8. ^{13}C-^{1}H HSQC spectrum of **TPI-3Se** in DMSO-d_6 at 298K, related to STAR Methods.

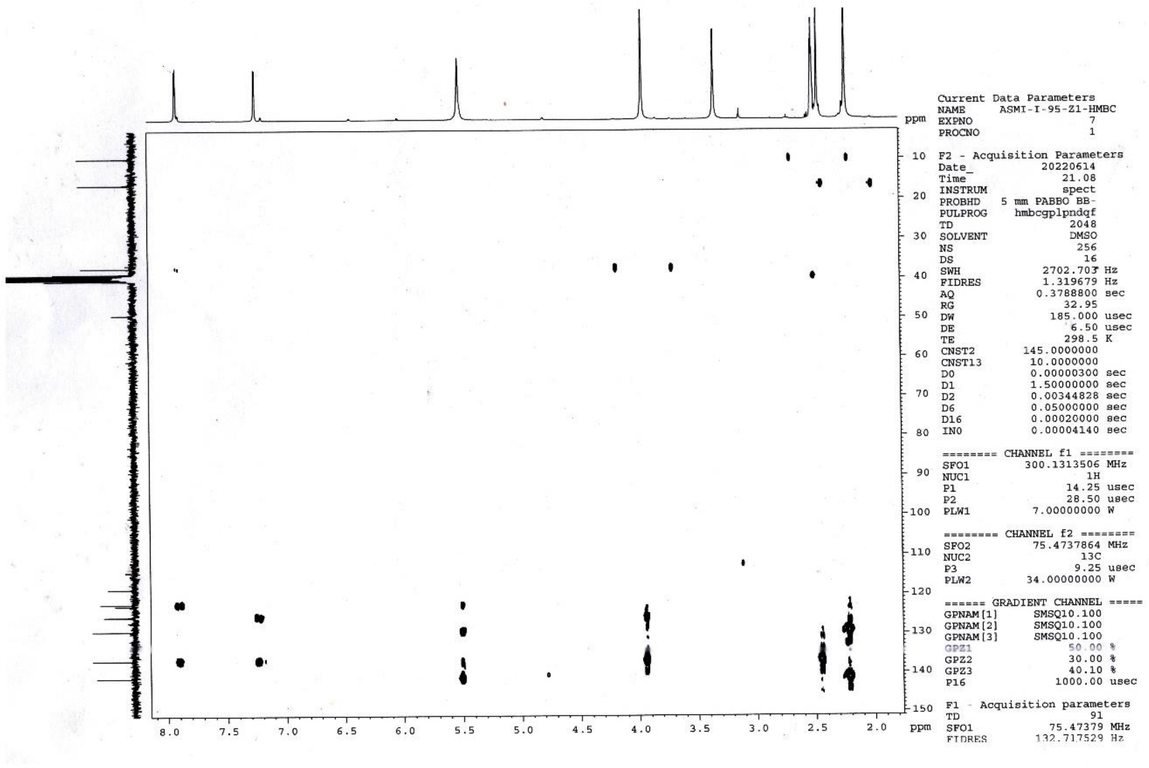

Figure S9. ^{13}C-^{1}H HMBC spectrum of **TPI-3Se** in DMSO-d_6 at 298K, related to STAR Methods.

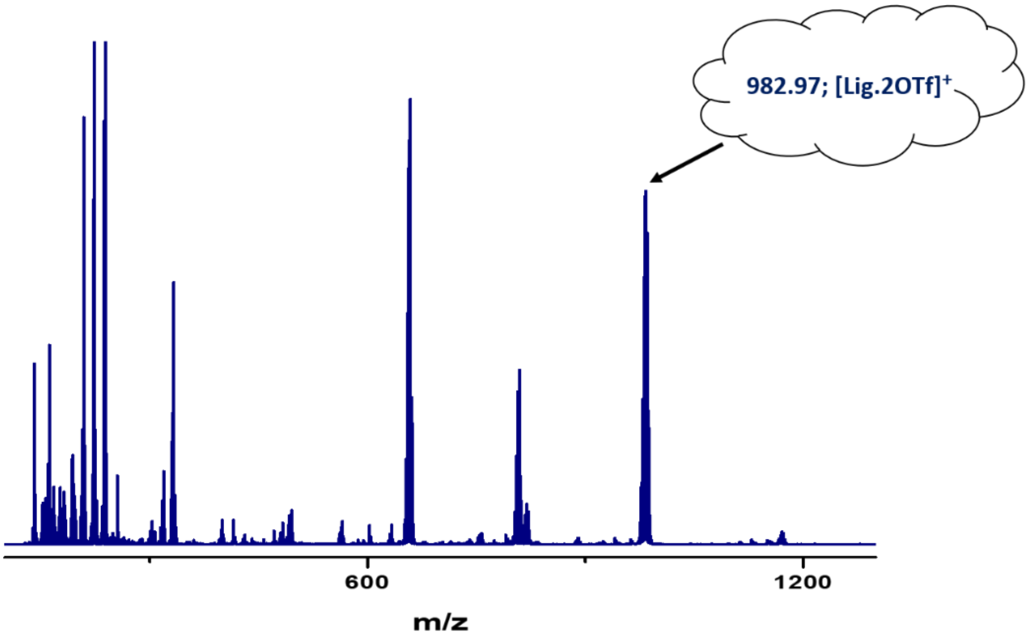

Figure S10. ESI-MS (+ve) spectrum **TPI-3Se** at 298K, related to STAR Methods.

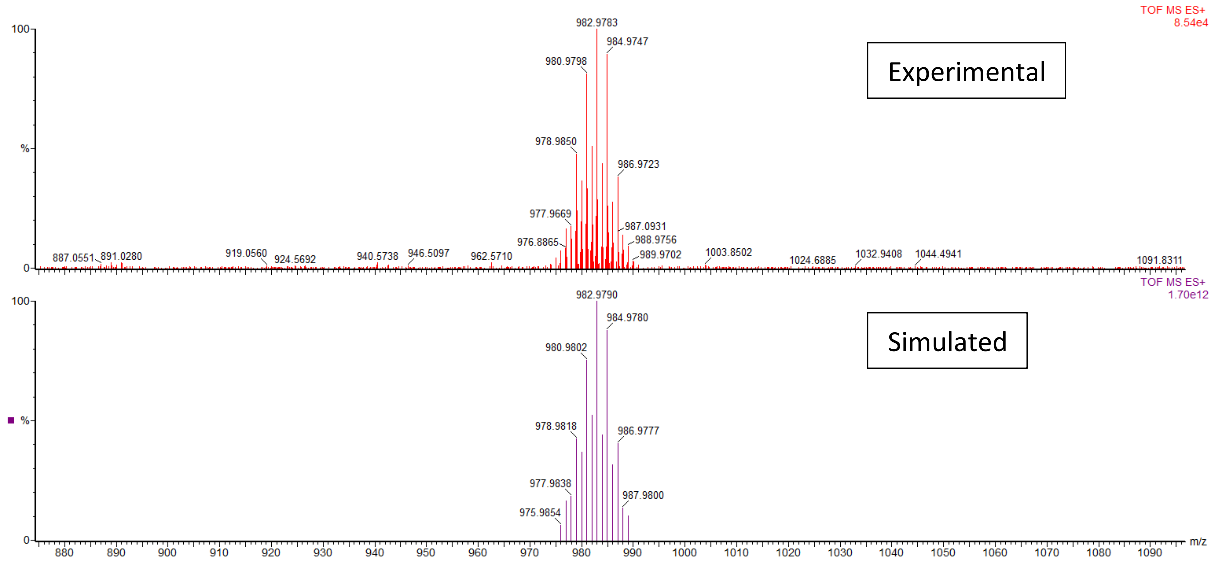

Figure S11. Isotropic distribution of ESI-MS (+ve) spectrum of [Lig.2OTf]$^+$ at 298K, related to STAR Methods.

Hydrogen bond donor TPI-3H:

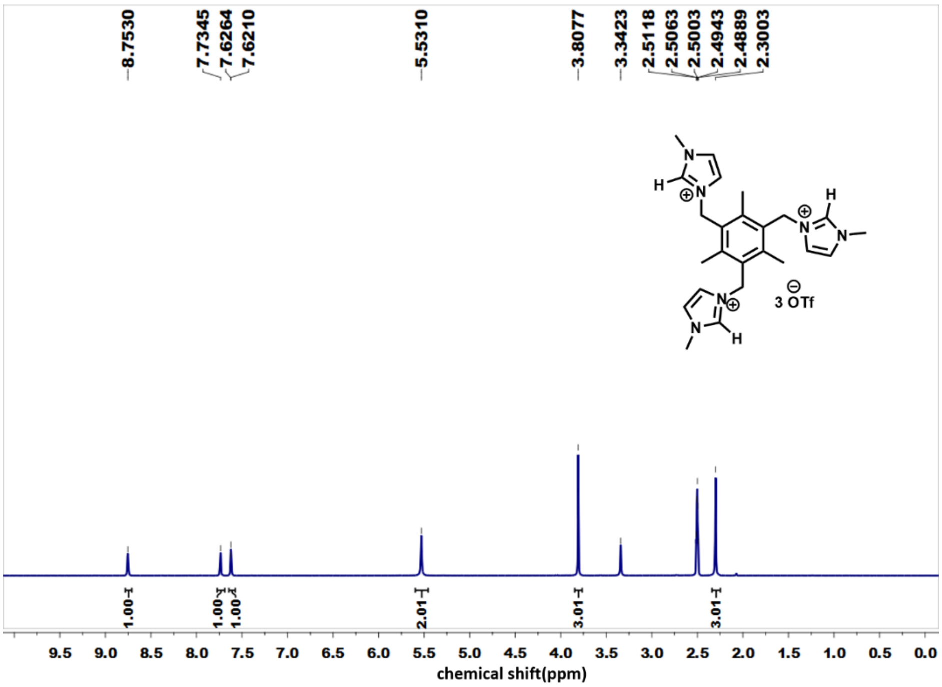

Figure S12. ^{1}H NMR spectrum of **TPI-3H** (300 MHz, DMSO-d_6, 298K), related to STAR Methods.

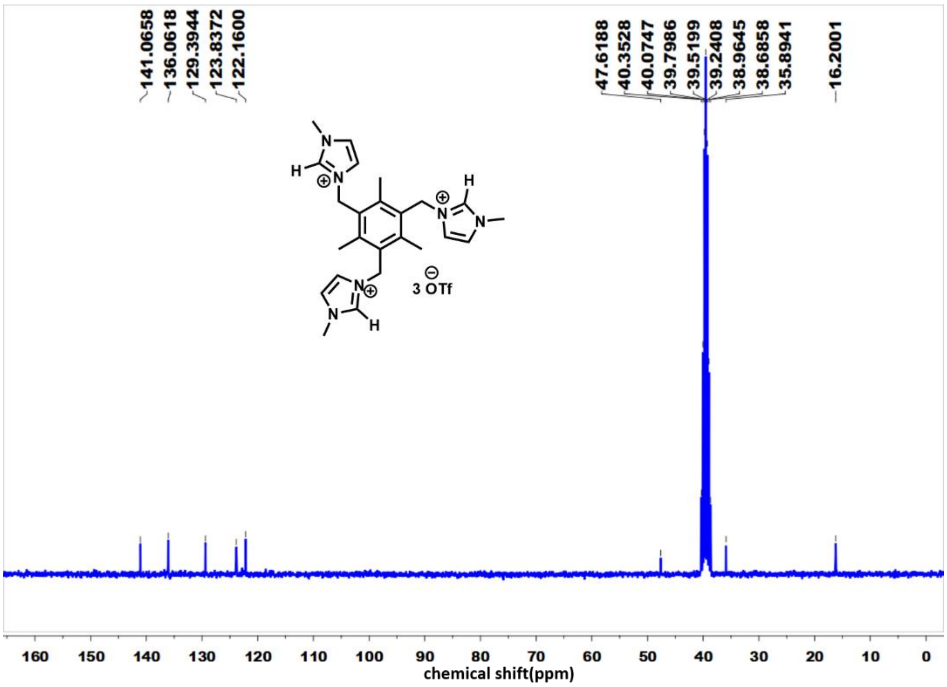

Figure S13. ^{13}C NMR spectrum of **TPI-3H** (75 MHz, DMSO-d_6, 298K), related to STAR Methods.

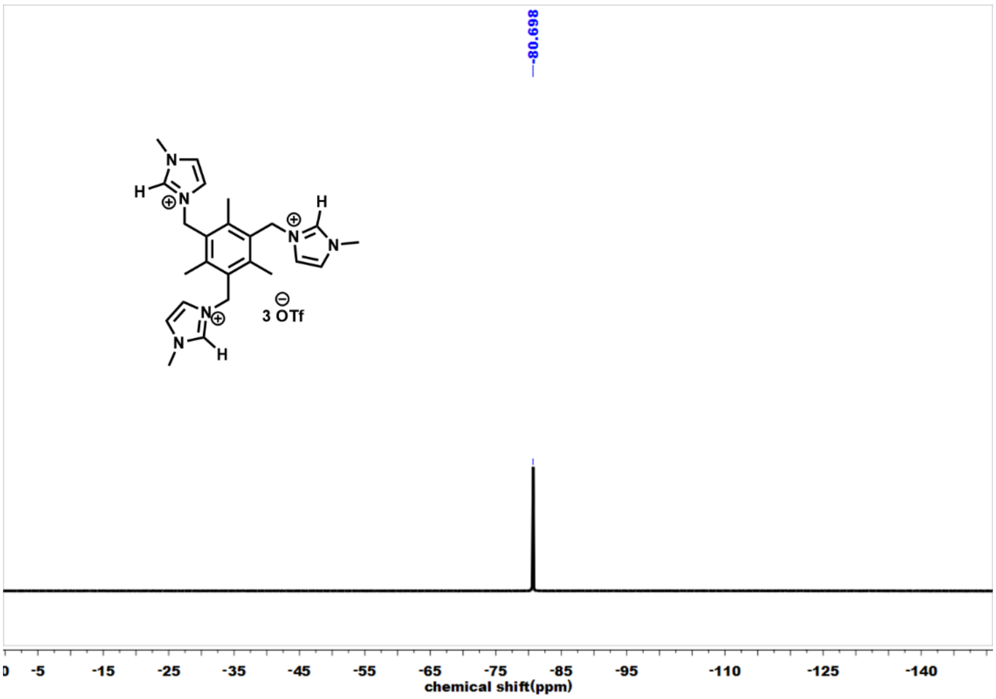

Figure S14. ^{19}F NMR spectrum of **TPI-3H** (400 MHz, DMSO-d_6, 298K), related to STAR Methods.

Halogen bond donor TPI-3Se:

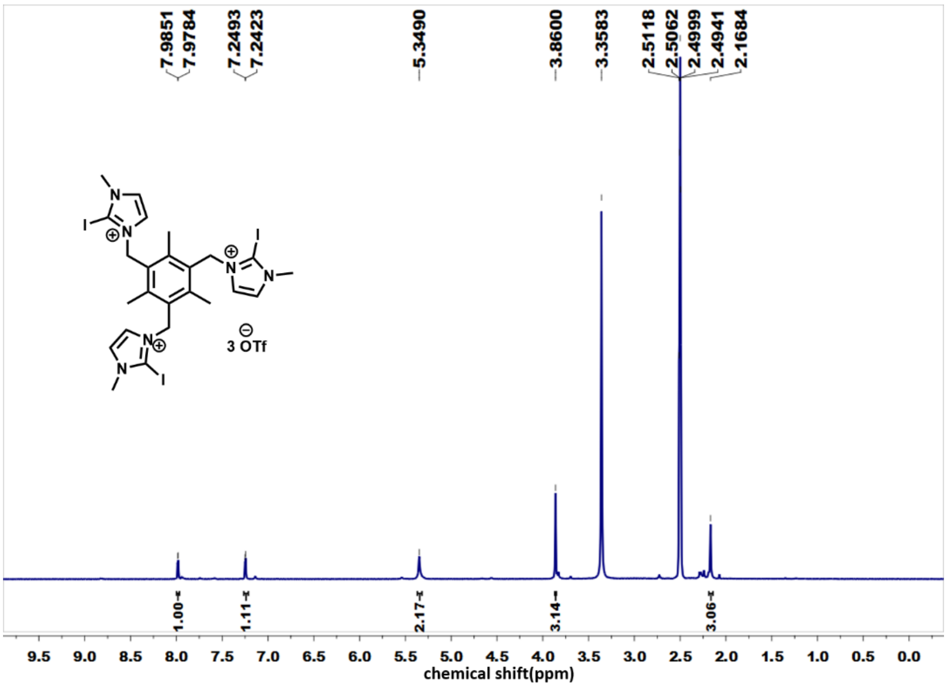

Figure S15. ^{1}H NMR spectrum of **TPI-3I** (300 MHz, DMSO-d_6, 298K), related to STAR Methods

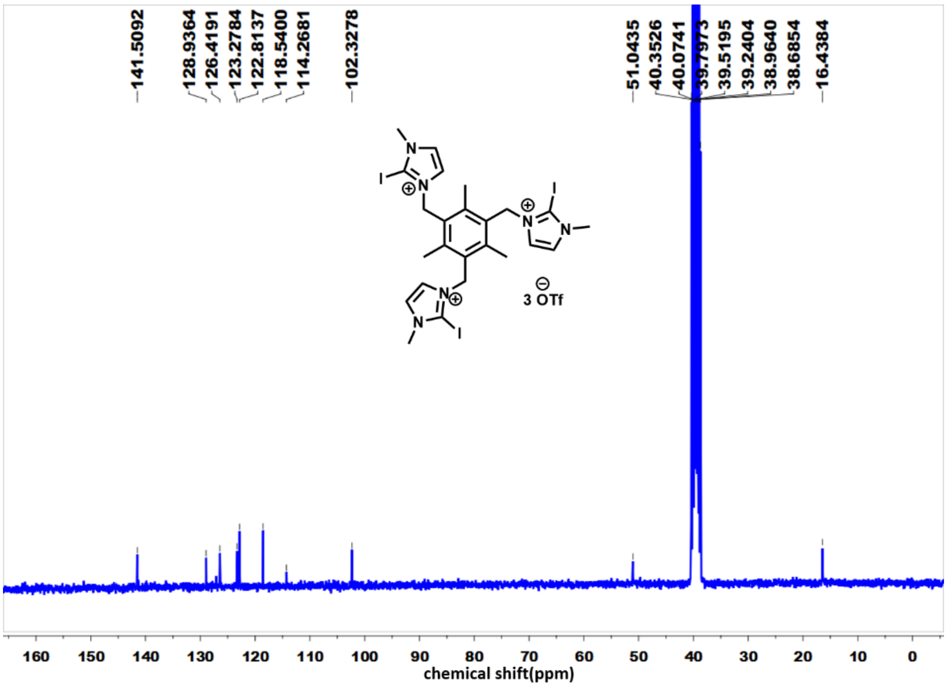

Figure S16. ^{13}C NMR spectrum of **TPI-3I** (75 MHz, DMSO-d_6, 298K), related to STAR Methods.

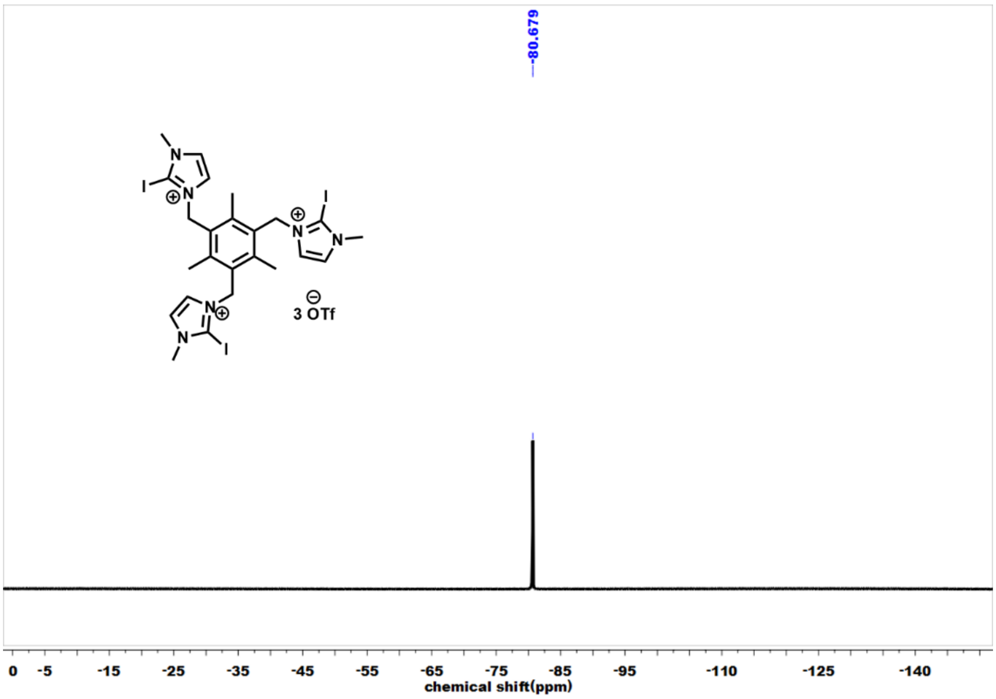

Figure S17. ^{19}F NMR spectrum of **TPI-3I** (400 MHz, DMSO-d_6, 298K), related to STAR Methods.

NMR Titration Studies:

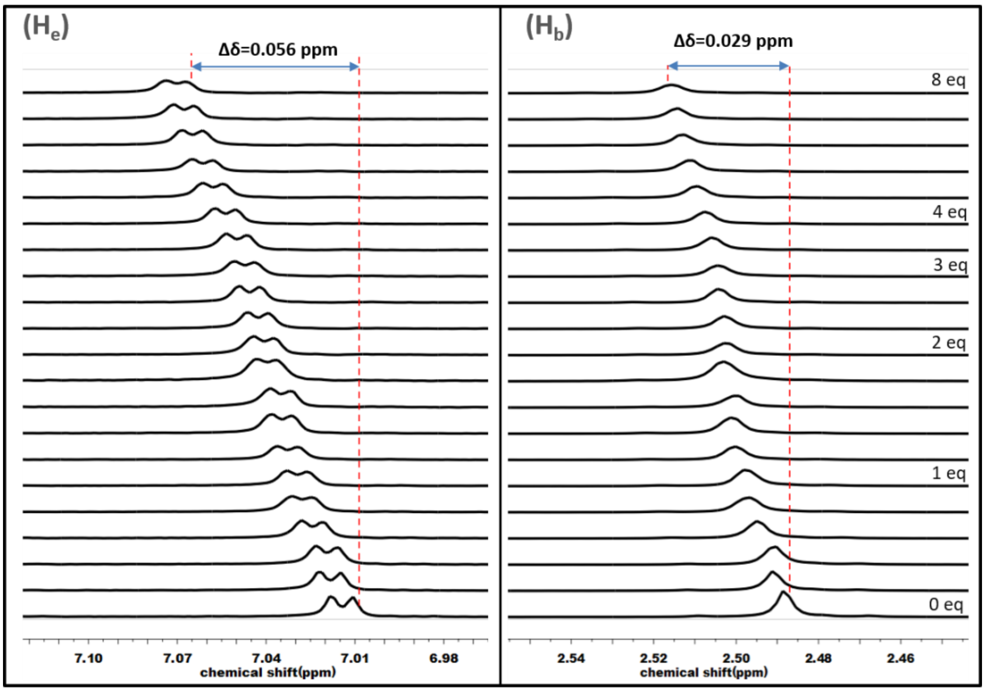

Figure S18. ^{1}H-NMR titration plot of TPI-3Se with NaI in D_2O, related to STAR Methods.

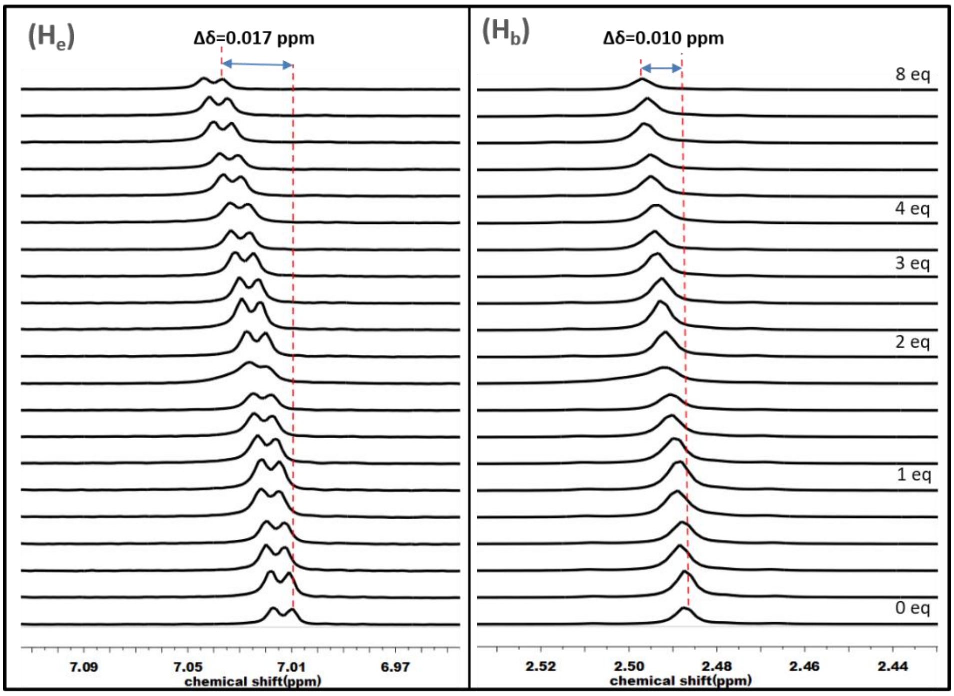

Figure S19. ^{1}H-NMR titration plot of TPI-3Se with NaBr in D_2O, related to STAR Methods.

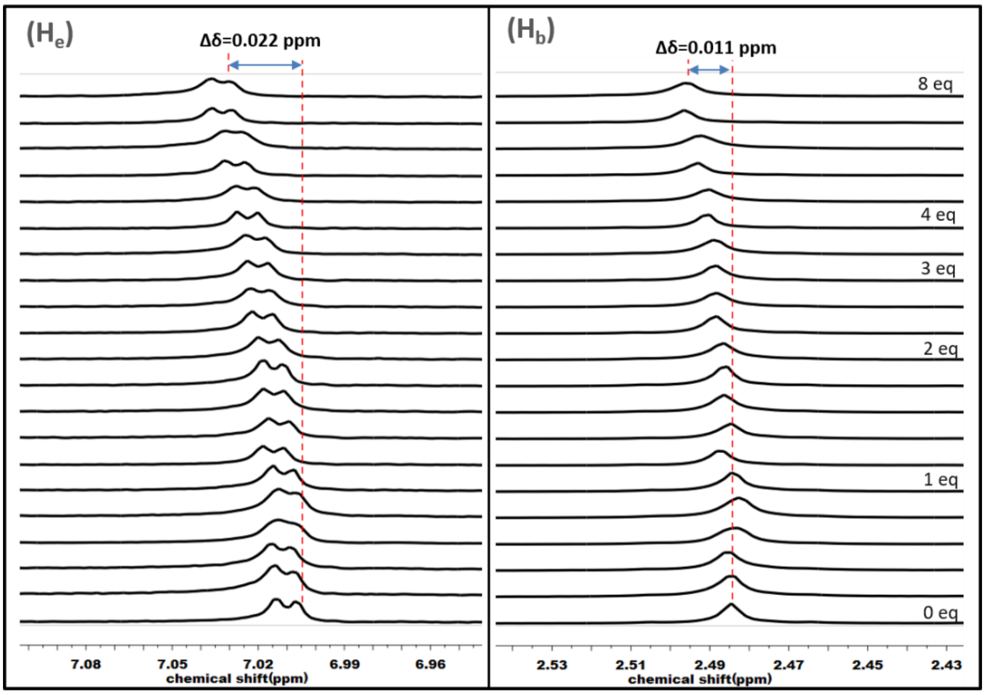

Figure S20. ^{1}H-NMR titration plot of TPI-3Se with NaCl in D_2O, related to STAR Methods.

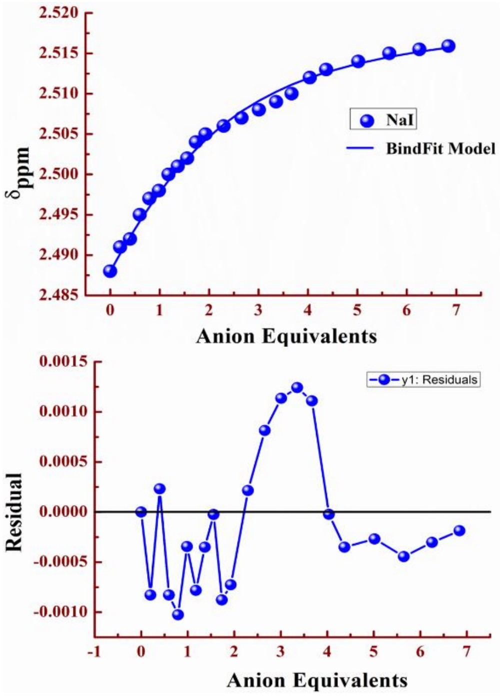

Figure S21. ^{1}H NMR spectral changes of the Se-Me proton of TPI-3Se in D_2O upon addition of increasing amounts of I^- anion along with its 1:1 fitting and corresponding residual plot for the I^- titration obtained from Bindfit software, related to STAR Methods.

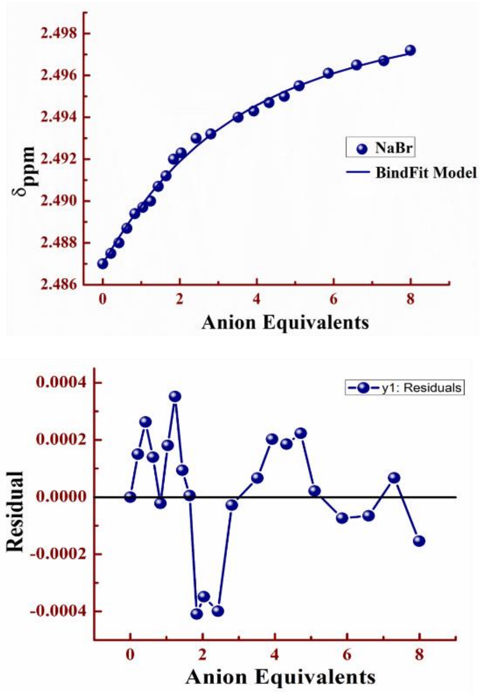

Figure S22. ^{1}H NMR spectral changes of the Se-Me proton of TPI-3Se in D_2O upon addition of increasing amounts of Br^- anion along with its 1:1 fitting and corresponding residual plot for the Br^- titration obtained from Bindfit software, related to STAR Methods.

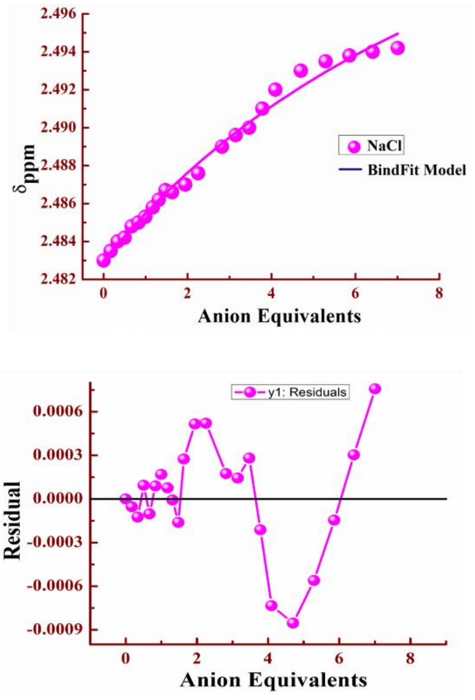

Figure S23. ^{1}H NMR spectral changes of the Se-Me proton of TPI-3Se in D_2O upon addition of increasing amounts of Cl^- anion along with its 1:1 fitting and corresponding residual plot for the Cl^- titration obtained from Bindfit software, related to STAR Methods.

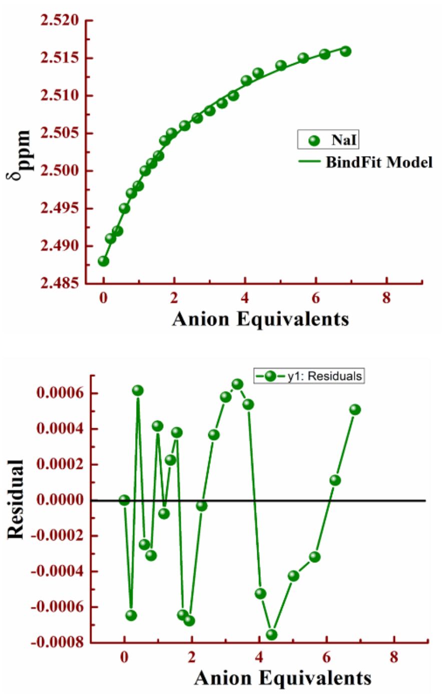

Figure S24. ^{1}H NMR spectral changes of the Se-Me proton of TPI-3Se in D_2O upon addition of increasing amounts of I^- anion along with its 1:2 fitting and corresponding residual plot for the I^- titration obtained from Bindfit software, related to STAR Methods.

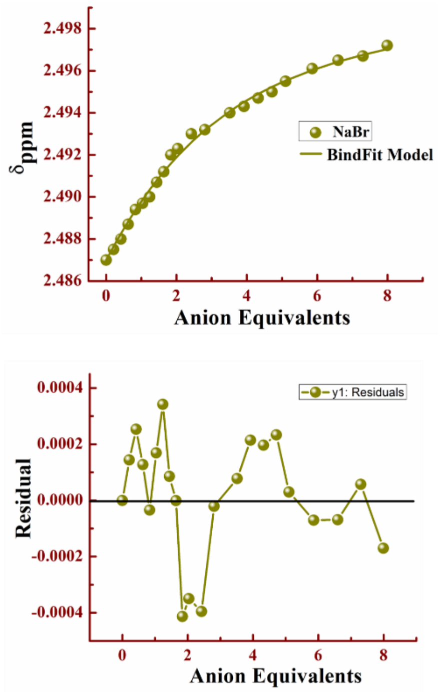

Figure S25. ^{1}H NMR spectral changes of the Se-Me proton of TPI-3Se in D_2O upon addition of increasing amounts of Br^- anion along with its 1:2 fitting and corresponding residual plot for the Br^- titration obtained from Bindfit software, related to STAR Methods.

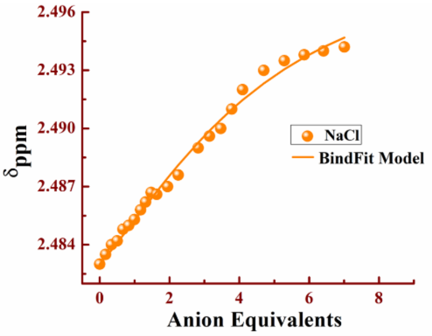

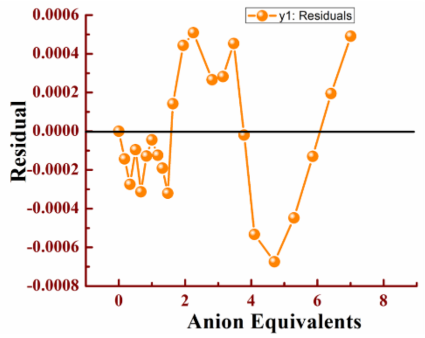

Figure S26. ^{1}H NMR spectral changes of the Se-Me proton of TPI-3Se in D_2O upon addition of increasing amounts of Cl^- anion along with its 1:2 fitting; Corresponding residual plot for the Cl^- titration obtained from Bindfit software, related to STAR Methods.

Table S1. Association Constants K_I and K_{II} (M^{-1}) of TPI-3Se in 1:2 halide binding in D_2O, related to STAR Methods.

Anion	K_I / M^{-1} (D_2O) TPI-3Se	K_{II} / M^{-1} (D_2O) TPI-3Se
Cl^-	17 ± 19%	12 ± 25%
Br^-	97 ± 15%	26 ± 14%
I^-	1052 ± 39%	25 ± 6%

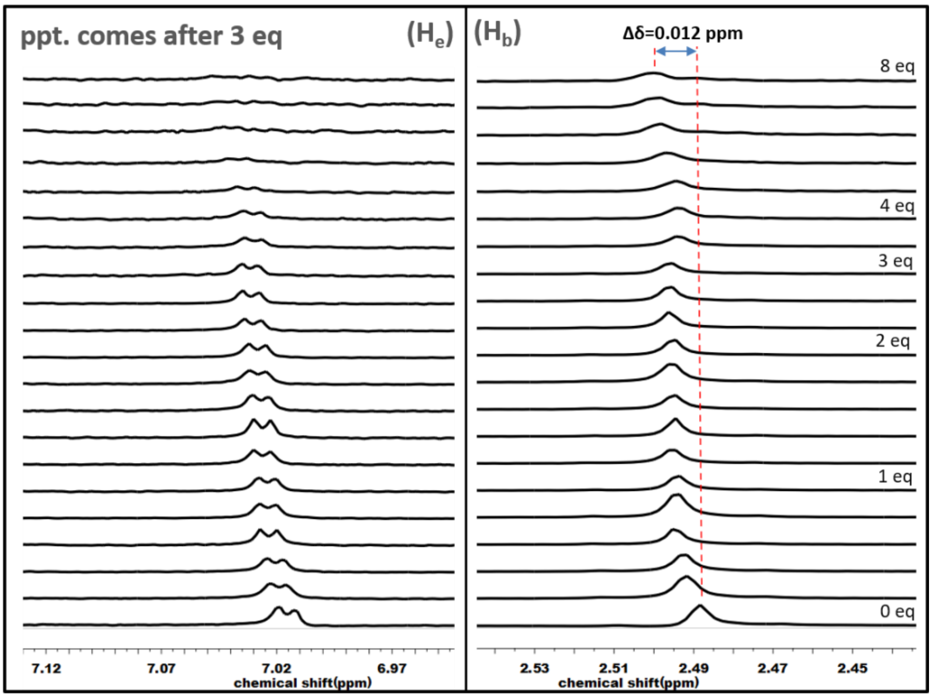

Figure S27. ^{1}H-NMR titration plot of TPI-3Se with $NaReO_4$ in D_2O, related to STAR Methods.

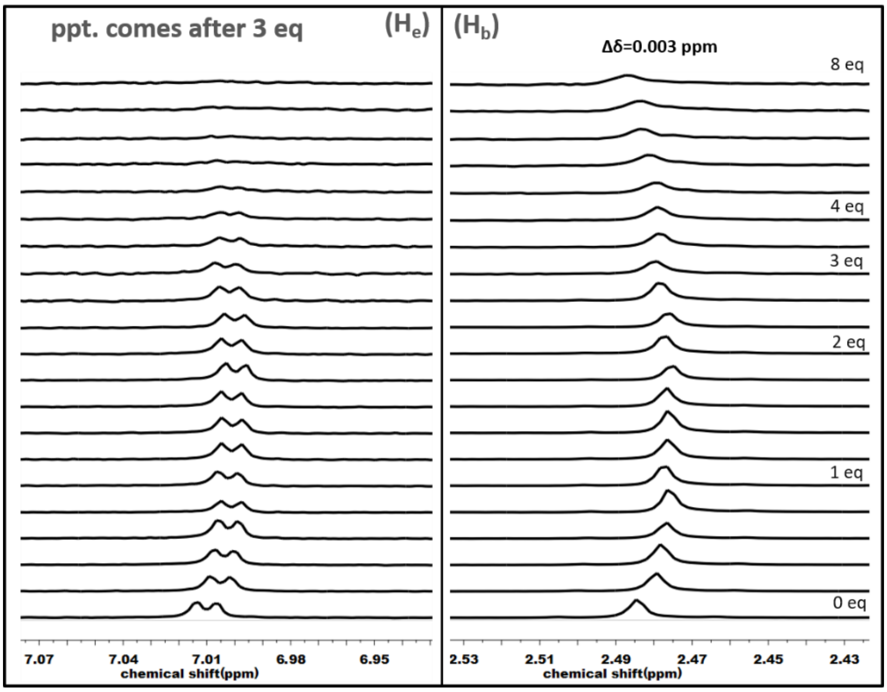

Figure S28. ^{1}H-NMR titration plot of TPI-3Se with $NaClO_4$ in D_2O, related to STAR Methods.

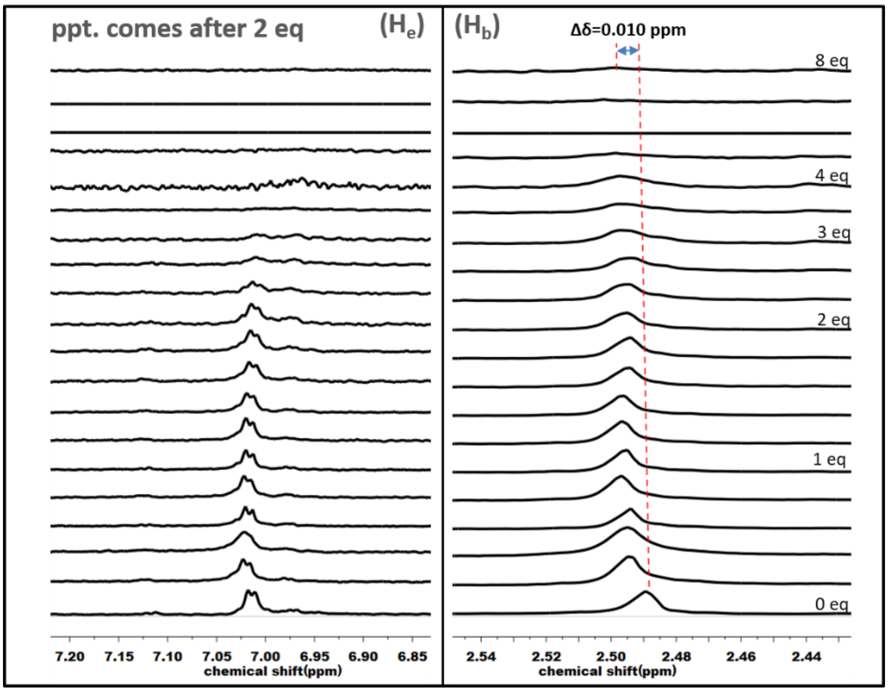

Figure S29. ^{1}H-NMR titration plot of TPI-3Se with $NaPF_6$ in D_2O, related to STAR Methods.

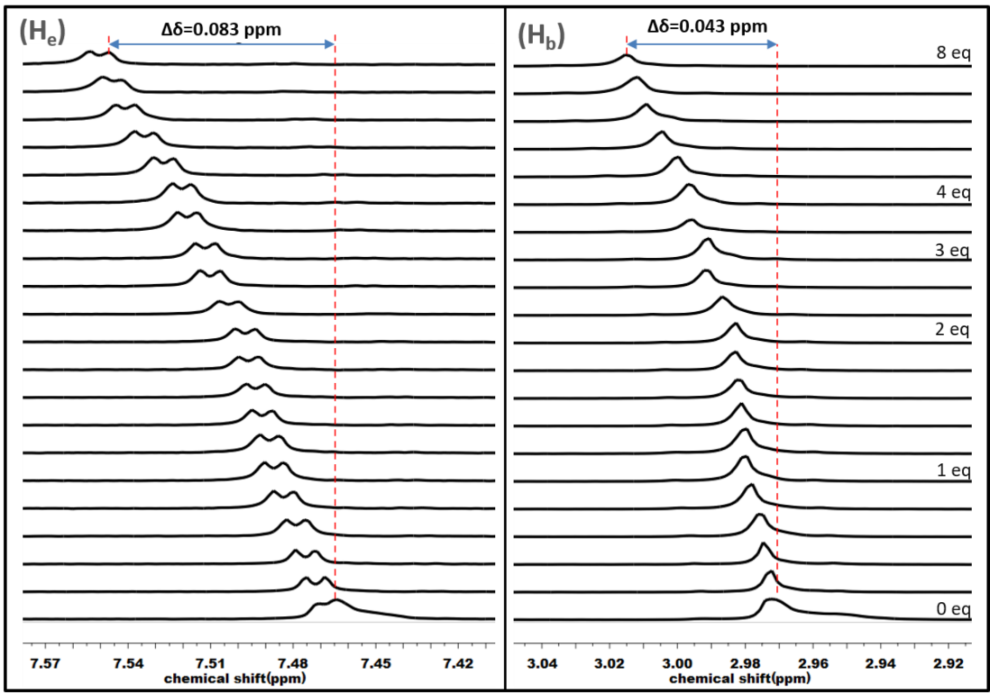

Figure S30. ^{1}H-NMR titration plot of TPI-3Se with NaI in D_2O/CD_3CN (1:1), related to STAR Methods.

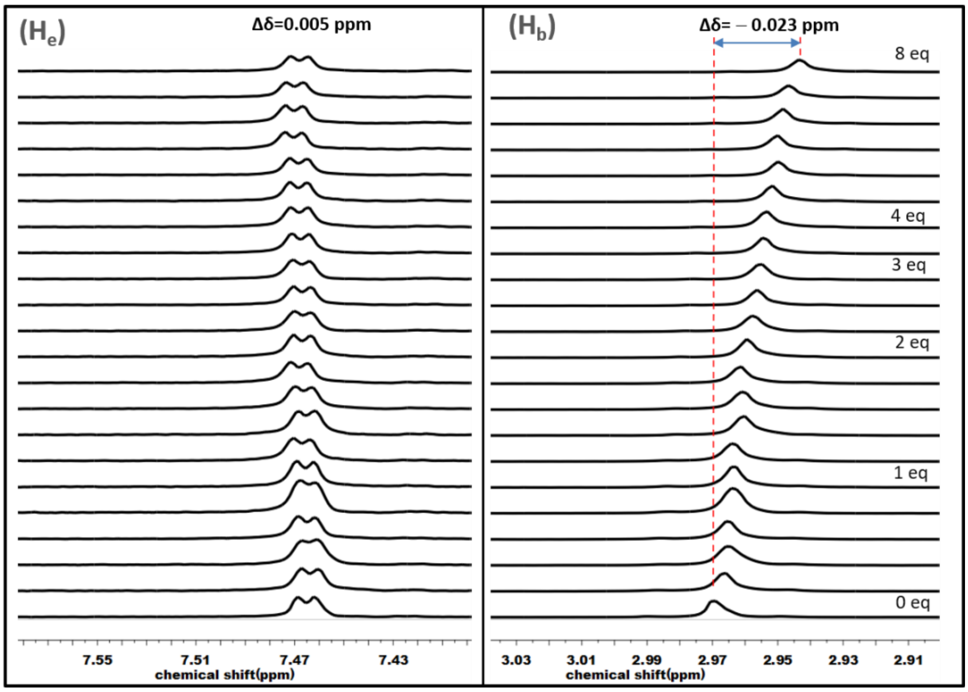

Figure S31. ^{1}H-NMR titration plot of TPI-3Se with NaBr in D_2O/CD_3CN (1:1), related to STAR Methods.

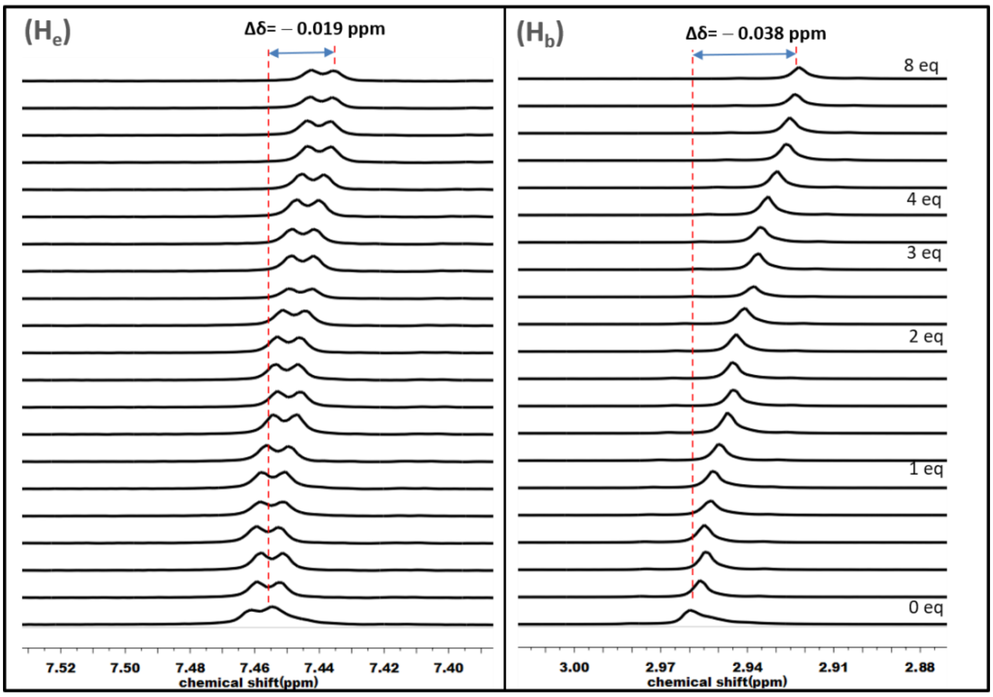

Figure S32. ^{1}H-NMR titration plot of TPI-3Se with NaCl in D_2O/CD_3CN (1:1), related to STAR Methods.

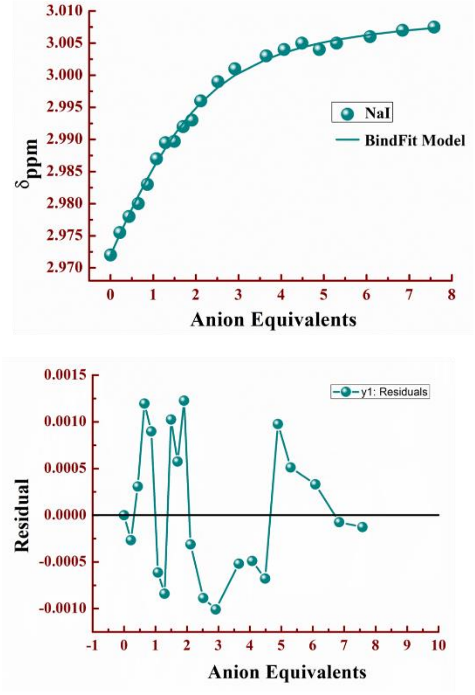

Figure S33. ^{1}H NMR spectral changes of the Se-Me proton of TPI-3Se in D_2O/CD_3CN (1:1) upon addition of increasing amounts of I^- anion along with its 1:1 fitting; Corresponding residual plot for the I^- titration obtained from Bindfit software, related to STAR Methods.

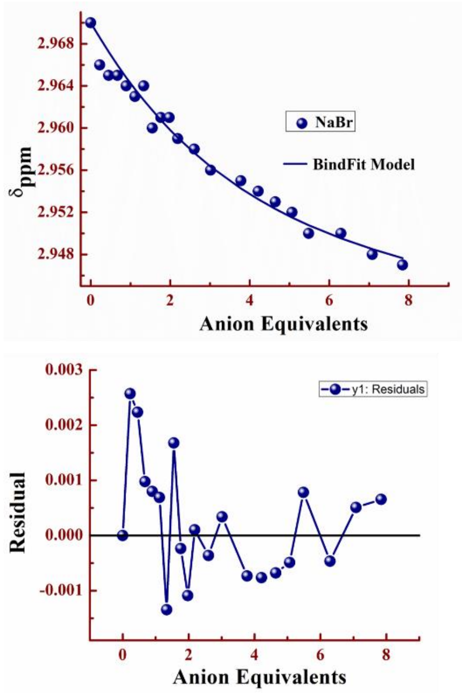

Figure S34. ^{1}H NMR spectral changes of the Se-Me proton of TPI-3Se in D_2O/CD_3CN (1:1) upon addition of increasing amounts of Br^- anion along with its 1:1 fitting; Corresponding residual plot for the Br^- titration obtained from Bindfit software, related to STAR Methods.

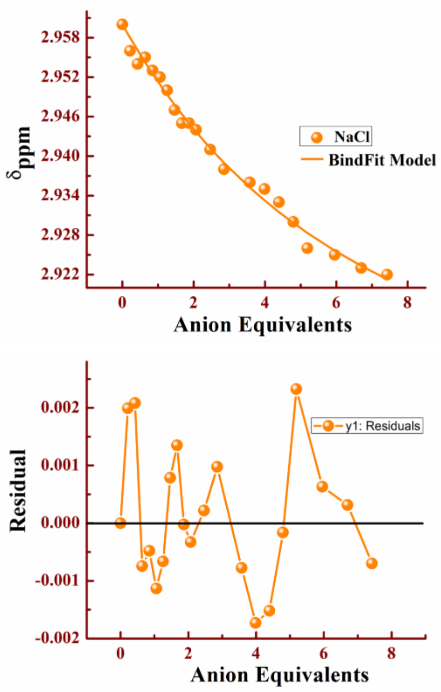

Figure S35. ^{1}H NMR spectral changes of the Se-Me proton of TPI-3Se in D_2O/CD_3CN (1:1) upon addition of increasing amounts of Cl^- anion along with its 1:1 fitting; Corresponding residual plot for the Cl^- titration obtained from Bindfit software, related to STAR Methods.

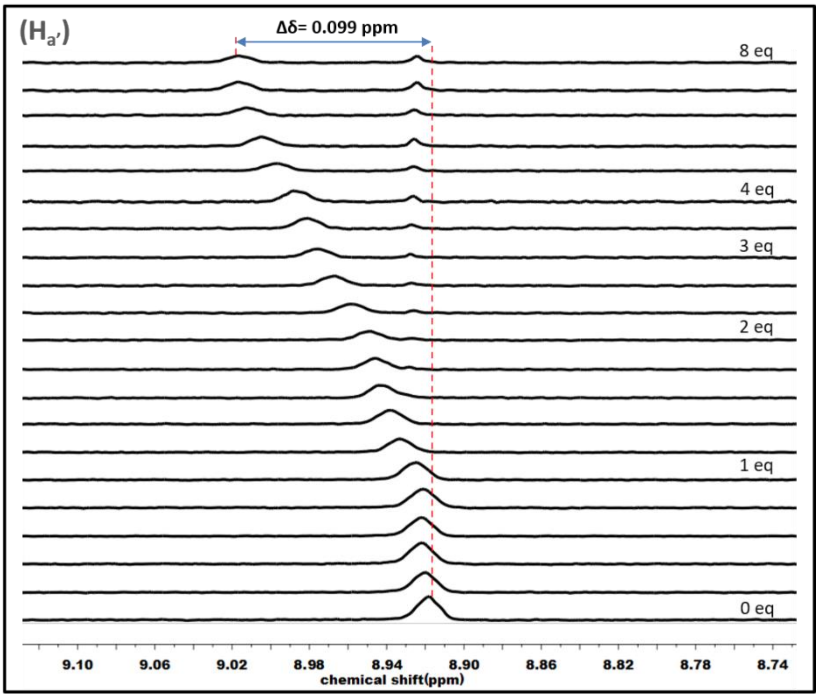

Figure S36. ^{1}H-NMR titration plot of TPI-3H with NaI in D_2O/CD_3CN (1:1), related to STAR Methods.

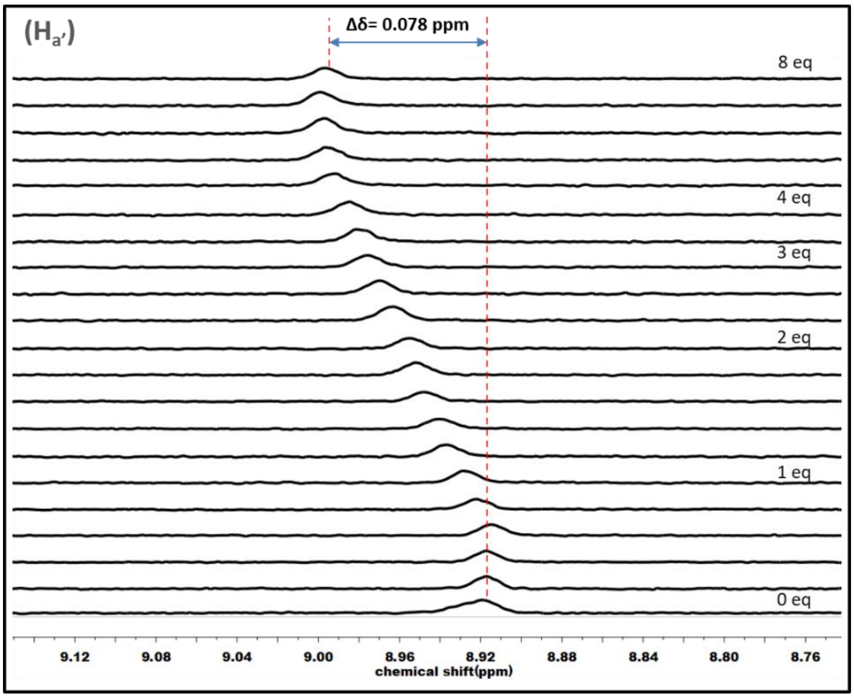

Figure S37. ^{1}H-NMR titration plot of TPI-3H with NaBr in D_2O/CD_3CN (1:1), related to STAR Methods.

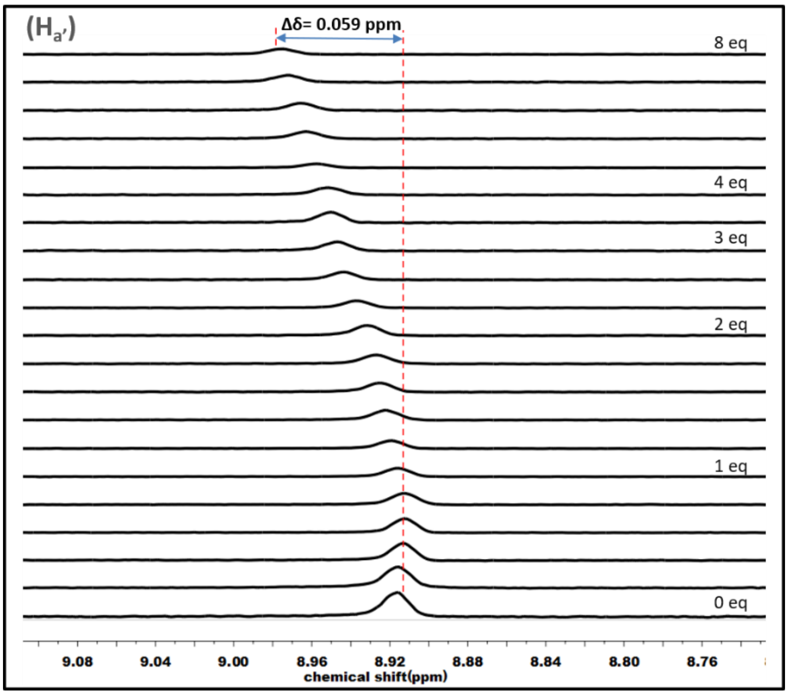

Figure S38. ^{1}H-NMR titration plot of TPI-3H with NaCl in D_2O/CD_3CN (1:1), related to STAR Methods.

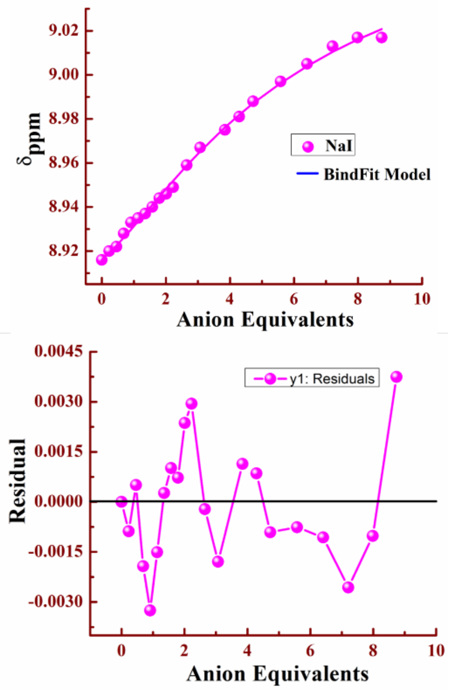

Figure S39. ^{1}H NMR spectral changes of the $H_{a'}$ proton of TPI-3H in D_2O/CD_3CN (1:1) upon addition of increasing amounts of I^- anion along with its 1:1 fitting; Corresponding residual plot for the I^- titration obtained from Bindfit software, related to STAR Methods.

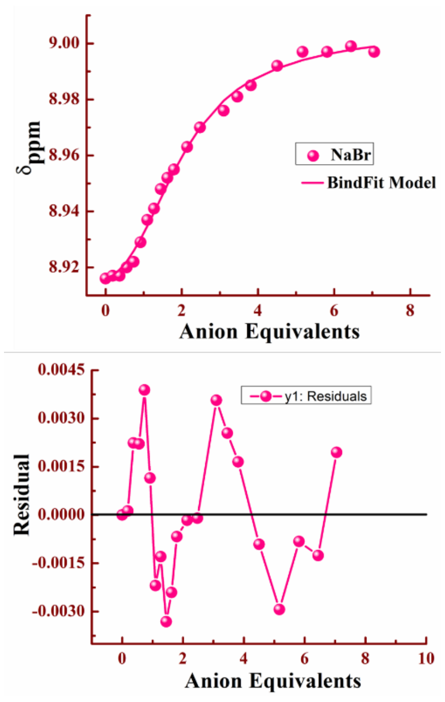

Figure S40. ^{1}H NMR spectral changes of the $H_{a'}$ proton of TPI-3H in D_2O/CD_3CN (1:1) upon addition of increasing amounts of Br^- anion along with its 1:1 fitting; Corresponding residual plot for the Br^- titration obtained from Bindfit software, related to STAR Methods.

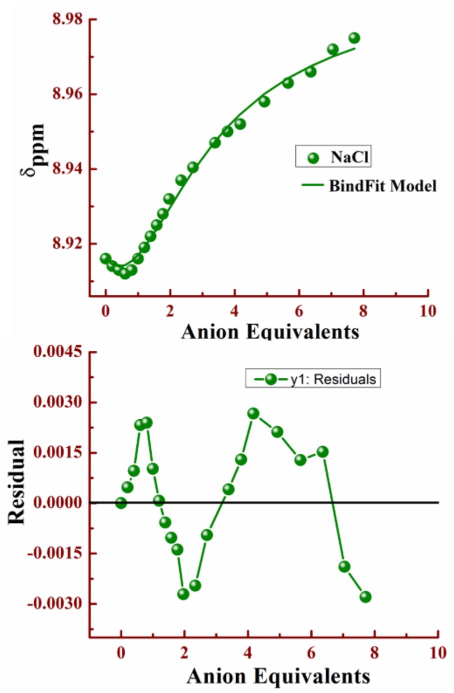

Figure S41. ^{1}H NMR spectral changes of the $H_{a'}$ proton of TPI-3H in D_2O/CD_3CN(1:1) upon addition of increasing amounts of Cl^- anion along with its 1:1 fitting; Corresponding residual plot for the Cl^- titration obtained from Bindfit software, related to STAR Methods.

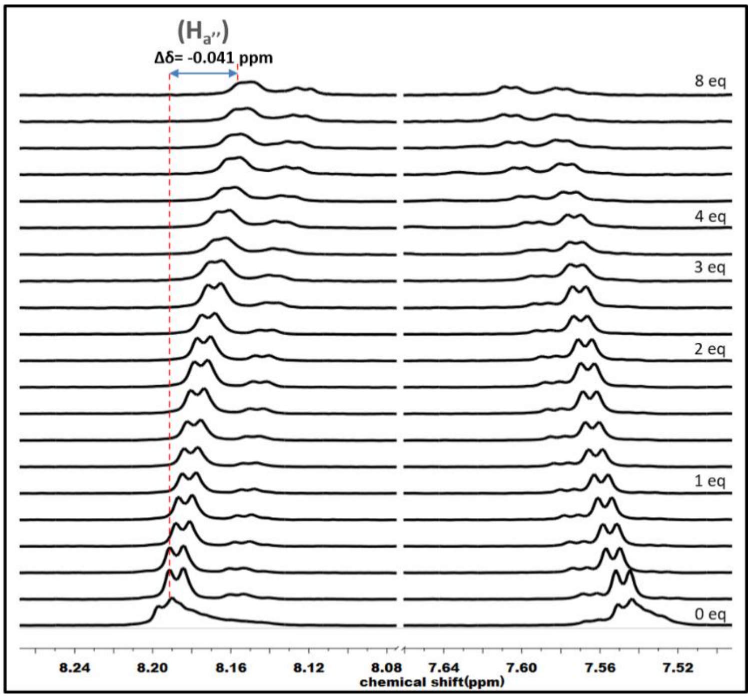

Figure S42. ^{1}H-NMR titration plot of TPI-3I with NaI in D_2O/CD_3CN (1:1), related to STAR Methods.

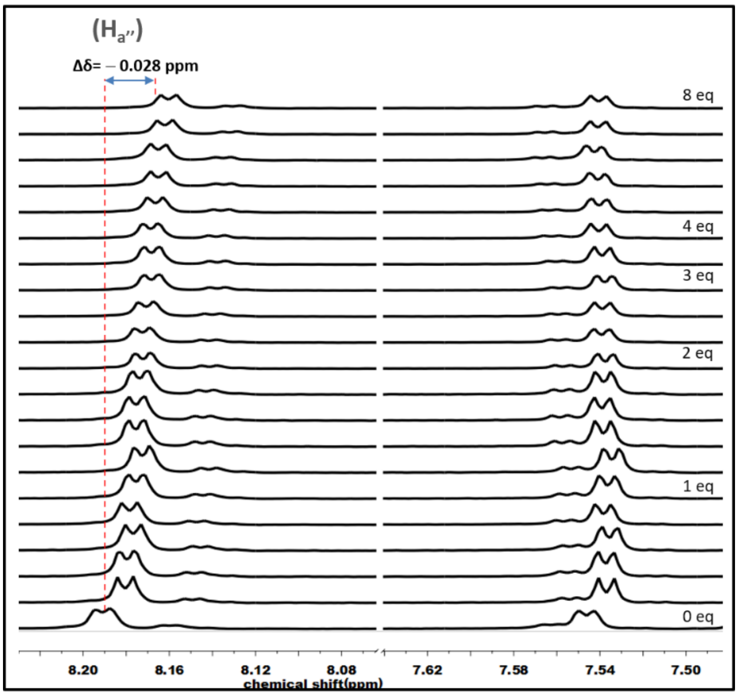

Figure S43. ^{1}H-NMR titration plot of TPI-3I with NaBr in D_2O/CD_3CN (1:1), related to STAR Methods.

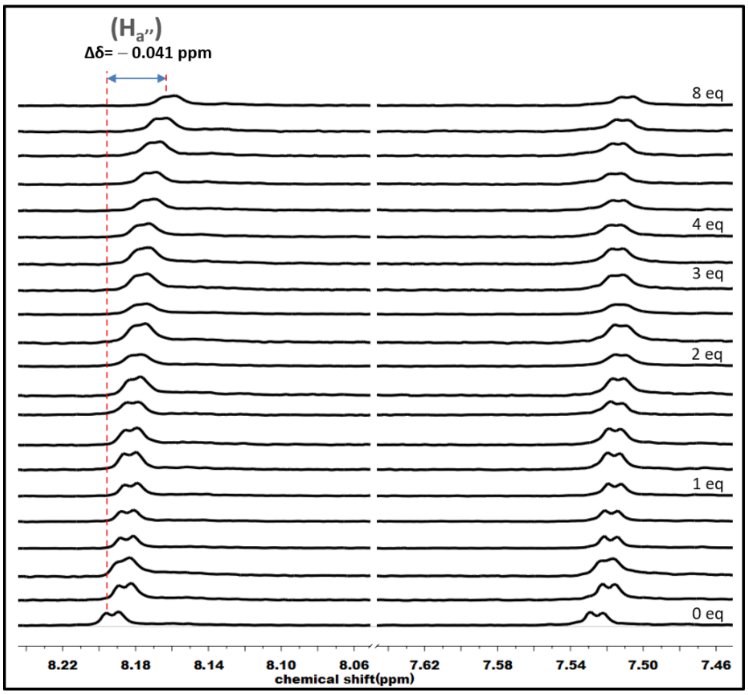

Figure S44. ^{1}H-NMR titration plot of TPI-3I with NaCl in D_2O/CD_3CN (1:1), related to STAR Methods.

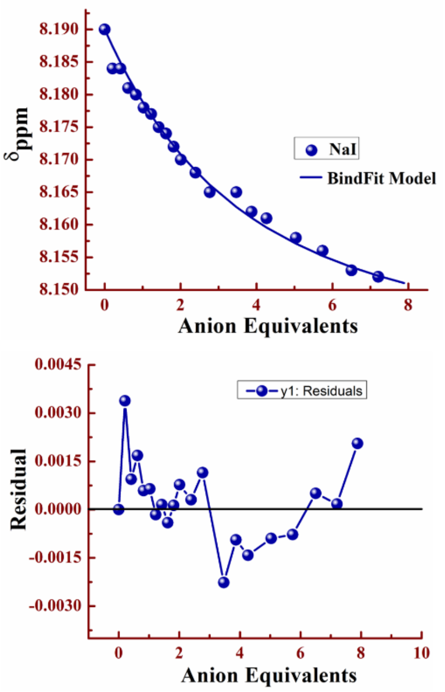

Figure S45. ^{1}H NMR spectral changes of the $H_{a''}$ proton of TPI-3I in D_2O/CD_3CN (1:1) upon addition of increasing amounts of I^- anion along with its 1:1 fitting; Corresponding residual plot for the I^- titration obtained from Bindfit software, related to STAR Methods.

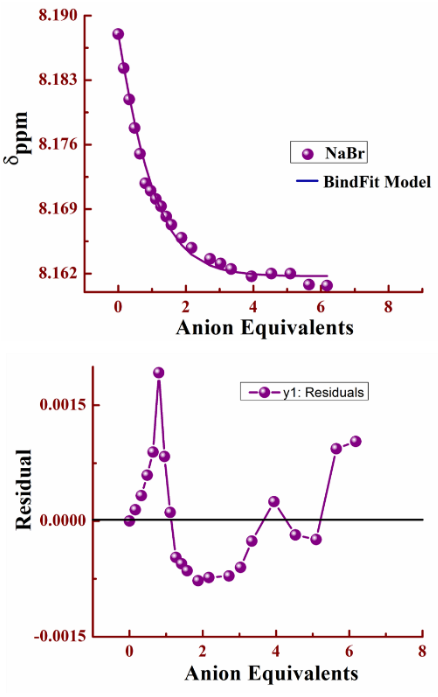

Figure S46. ^{1}H NMR spectral changes of the $H_{a''}$ proton of TPI-3I in D_2O/CD_3CN (1:1) upon addition of increasing amounts of Br^- anion along with its 1:1 fitting; Corresponding residual plot for the Br^- titration obtained from Bindfit software, related to STAR Methods.

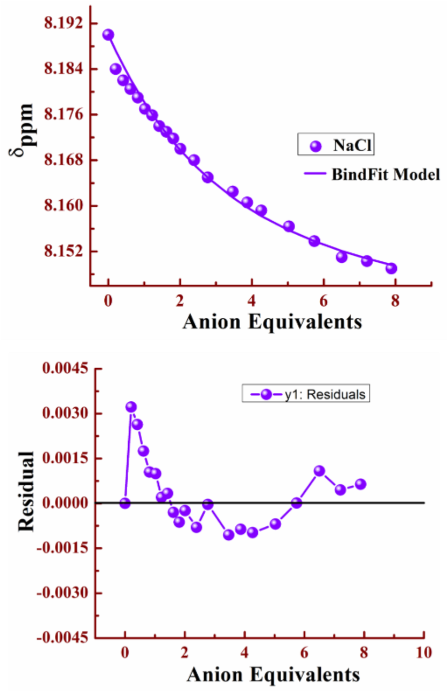

Figure S47. ^{1}H NMR spectral changes of the $H_{a''}$ proton of TPI-3I in D_2O/CD_3CN (1:1) upon addition of increasing amounts of Cl^- anion along with its 1:1 fitting; Corresponding residual plot for the Cl^- titration obtained from Bindfit software, related to STAR Methods.

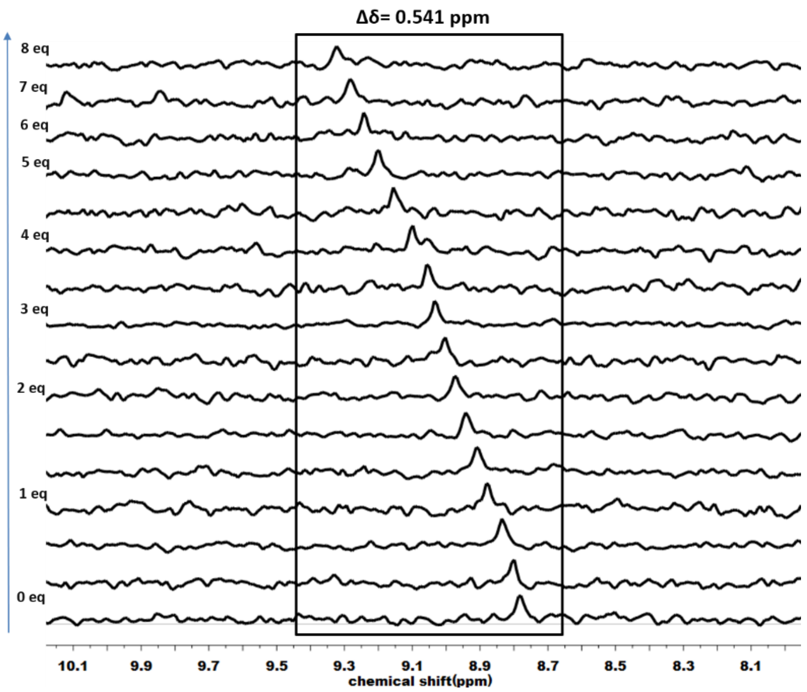

Figure S48. ^{13}C-NMR titration plot of **TPI-3Se** with NaI in 1:1 D_2O and CD_3CN, related to STAR Methods.

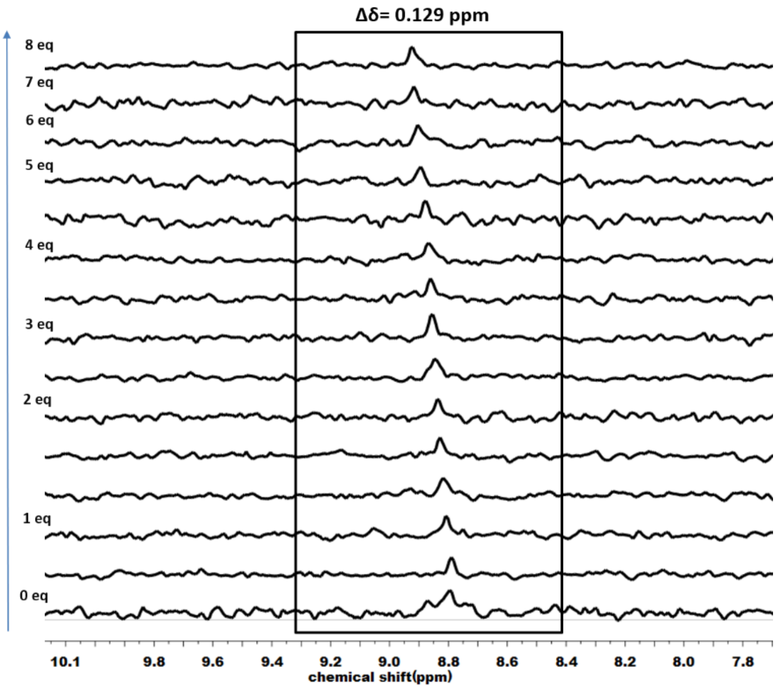

Figure S49. ^{13}C-NMR titration plot of **TPI-3Se** with NaBr in 1:1 D_2O and CD_3CN, related to STAR Methods.

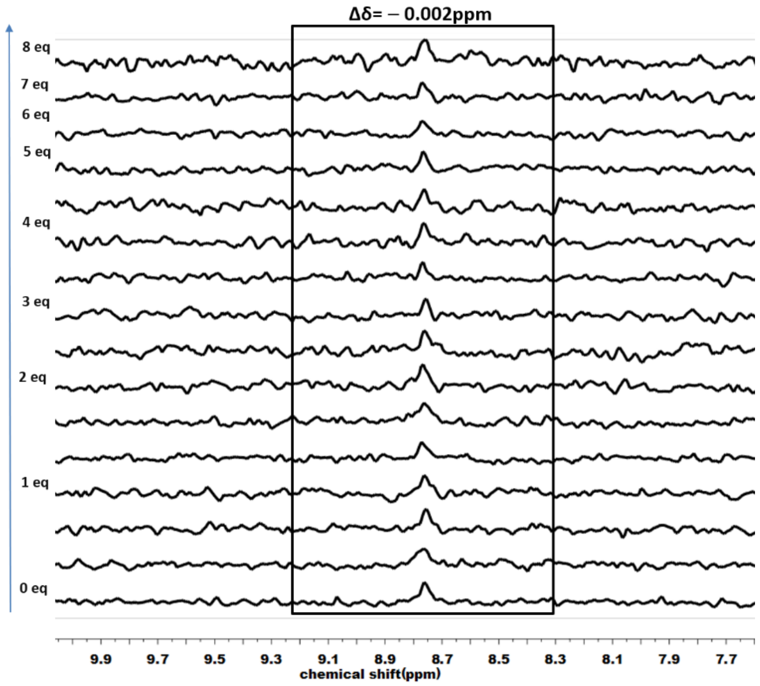

Figure S50. ^{13}C-NMR titration plot of **TPI-3Se** with NaCl in 1:1 D_2O and CD_3CN, related to STAR Methods.

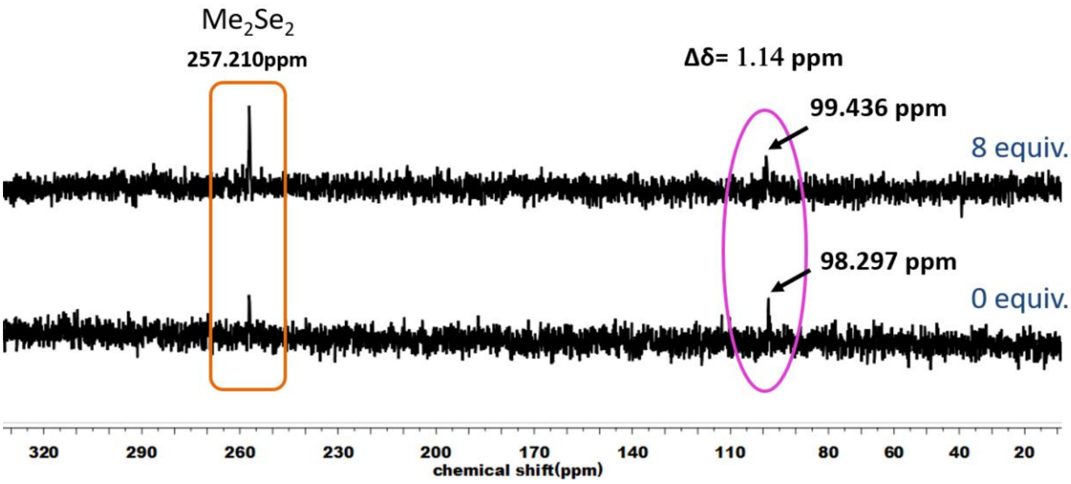

Figure S51. ^{77}Se-NMR titration plot of **TPI-3Se** with NaI (8 equiv.) in 1:1 D_2O and CD_3CN, related to STAR Methods.

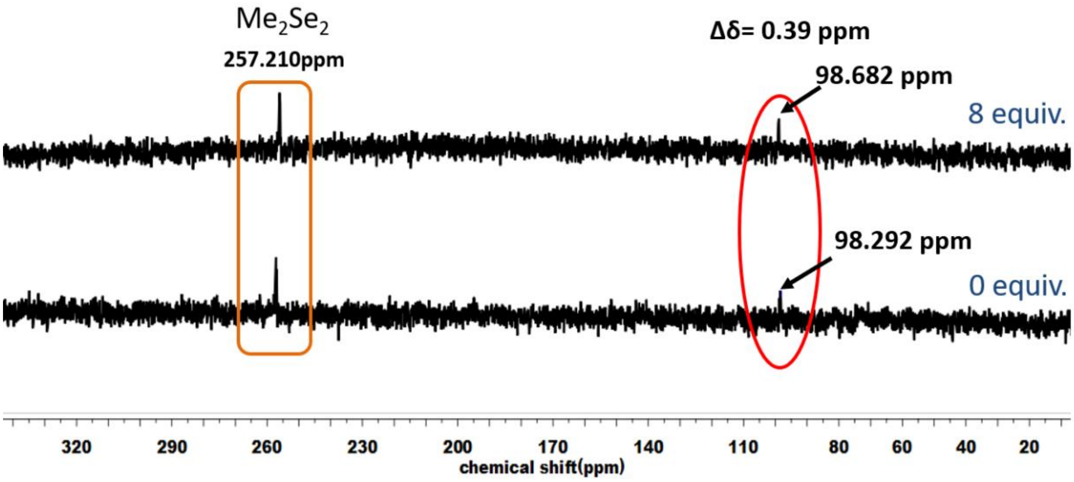

Figure S52. ^{77}Se-NMR titration plot of **TPI-3Se** with NaBr (8 equiv.) in 1:1 D_2O and CD_3CN, related to STAR Methods.

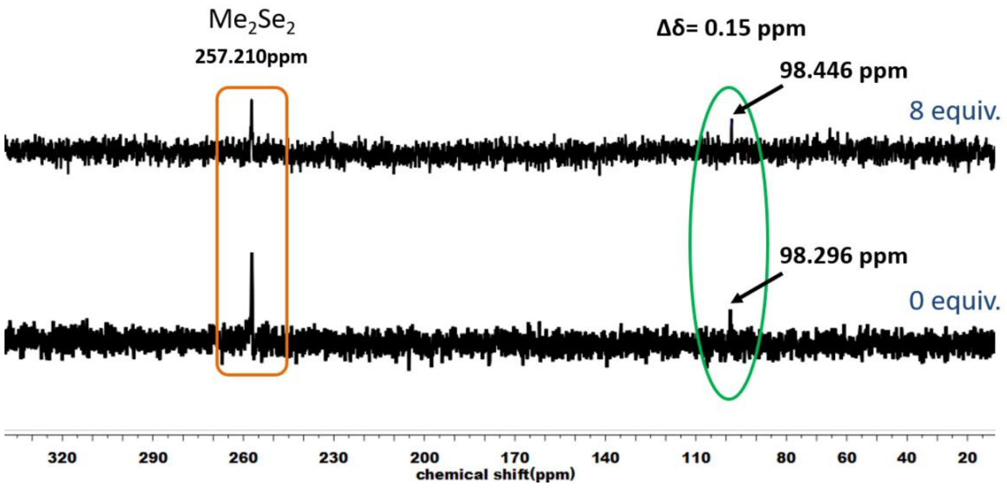

Figure S53. ^{77}Se-NMR titration plot of **TPI-3Se** with NaCl (8 equiv.) in 1:1 D_2O and CD_3CN, related to STAR Methods.

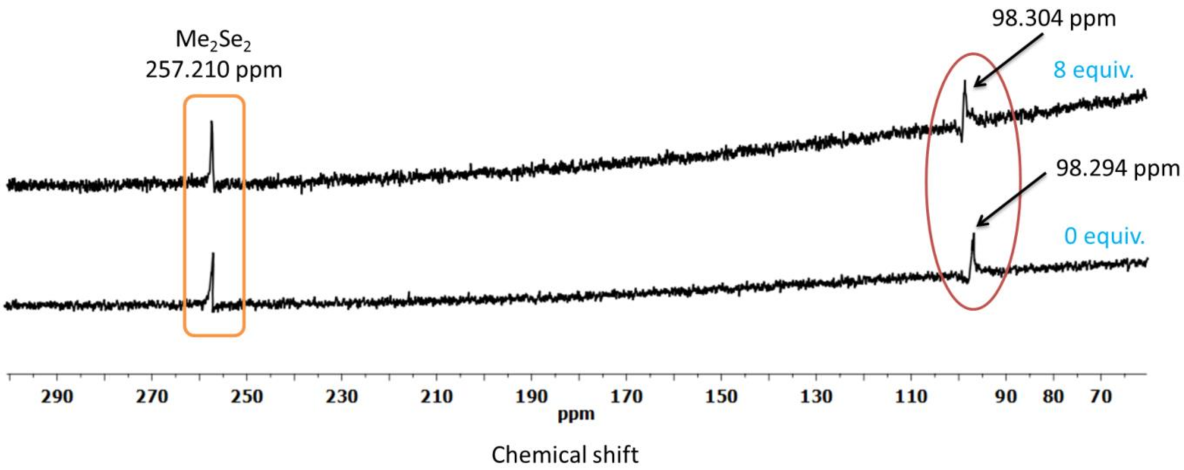

Figure S54. ^{77}Se-NMR titration plot of **TPI-3Se** with NaCN (8 equiv.) in 1:1 D_2O and CD_3CN, related to STAR Methods.

5. **Isothermal Titration Calorimetric (ITC) studies:**

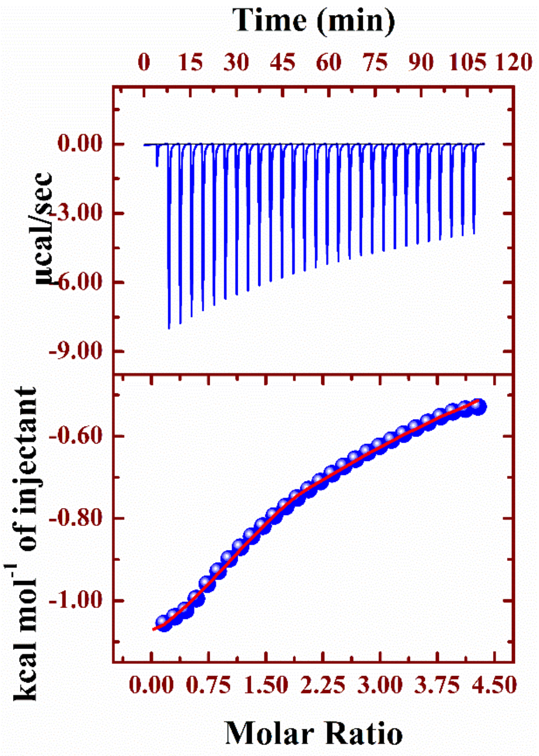

Figure S55. TPI-3Se (1 mM) was titrated with NaI (20 mM) in 1:1(v/v) water-acetonitrile solvent at 298K, related to STAR Methods.

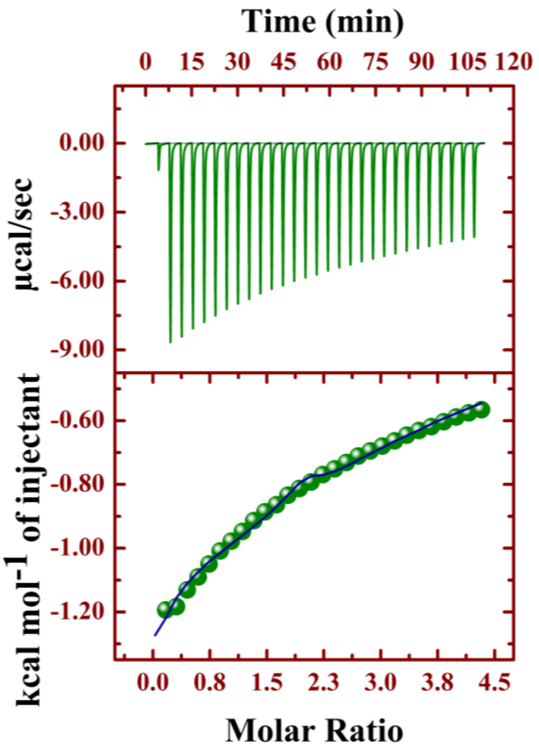

Figure S56. TPI-3Se (1 mM) was titrated with NaBr (20 mM) in 1:1(v/v) water-acetonitrile solvent at 298K, related to STAR Methods.

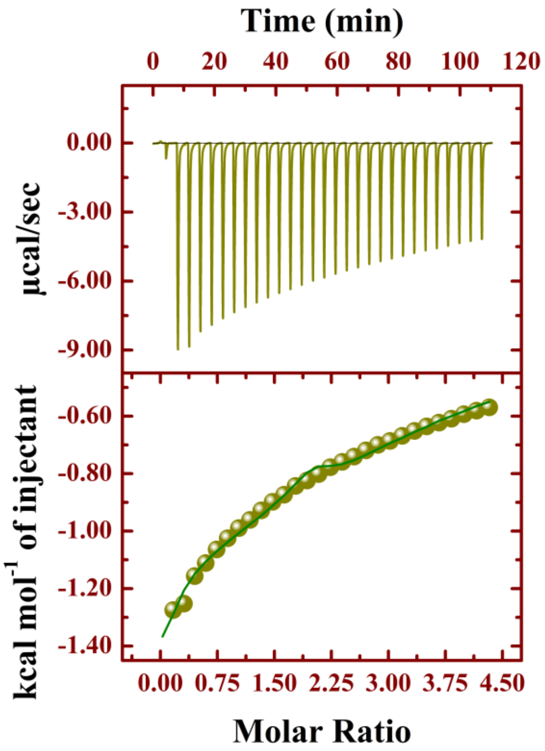

Figure S57. TPI-3Se (1 mM) was titrated with NaCl (20 mM) in 1:1(v/v) water-acetonitrile solvent at 298K, related to STAR Methods.

Crystal Information and Structure:

Table S2. Crystallographic details of the TPI-3Se Iodide crystal, related to Figure 1.

**Compound reference**	**TPI-3Se-I**
**Chemical formula**	$C_{27}H_{39}N_6Se_3$•3(I) •2(O)
**Formula Mass**	1097.22
**Crystal system**	monoclinic
***a*/Å**	7.4457(17)
***b*/Å**	23.996(6)
***c*/Å**	20.624(5)
***α*/°**	90
***β*/°**	97.573(7)
***γ*/°**	90
**Unit cell volume/Å^3**	3652.7(15)
**Temperature/K**	144
**Space group**	*P*21/n
**No. of formula units per unit cell, *Z***	4
**Radiation type**	MoKα
**No. of reflections measured**	28997
**No. of independent reflections**	5243
R_{int}	0.1034
**Final R_1 values ($I > 2\sigma(I)$)**	0.0425
**Final $wR(F^2)$ values ($I > 2\sigma(I)$)**	0.0982
**Final R_1 values (all data)**	0.0710
**Final $wR(F^2)$ values (all data)**	0.1197
**CCDC number**	2180385

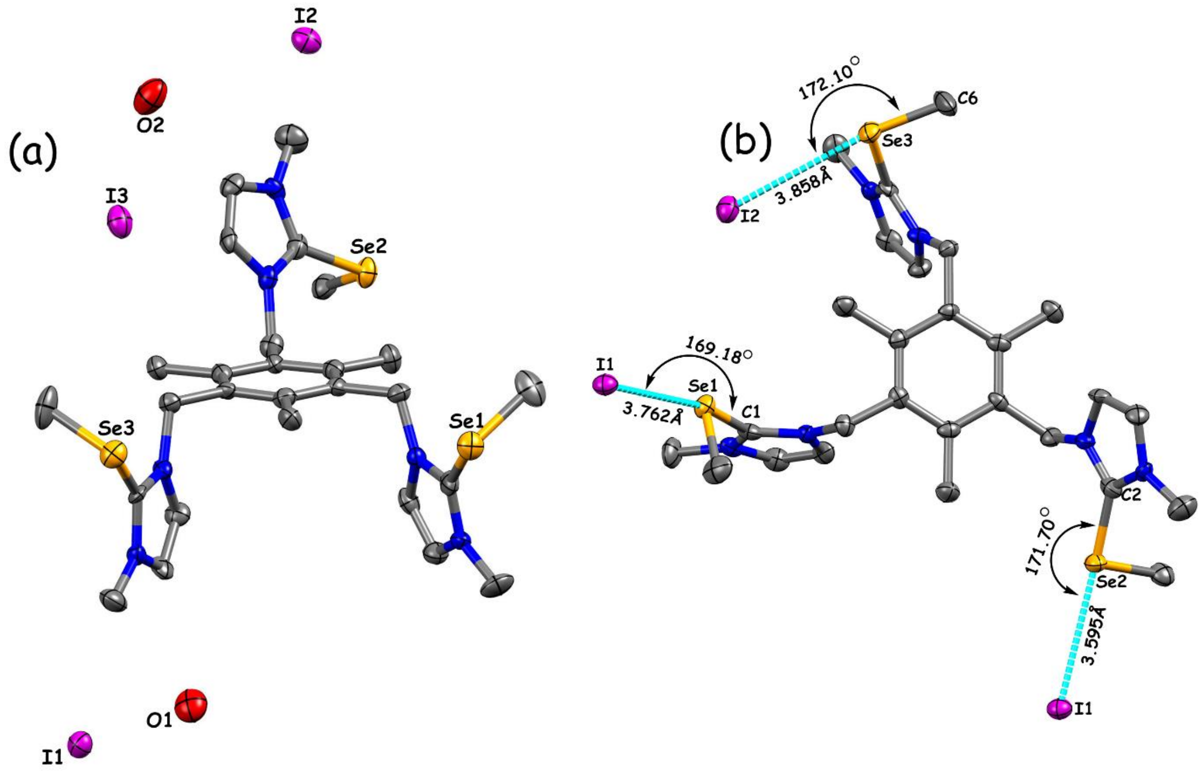

Figure S58. (a) ORTEP diagram of TPI-3Se-I. Thermal ellipsoids are shown at 50% probability. (b) Solid state structure of TPI-3Se-I, presented as linear chalcogen bonding interaction of C—Se•••I, related to Figure 1.

Table S3. Different Se•••I bond length and I•••Se-C bond angle obtained from the crystal of TPI-3Se iodide complex, related to Figure 1.

Bond Lengths (Å)		Bond Angles (º)	
Se1-I1	3.762	I1-Se2-C2 (imidazole)	171.70
Se2-I1	3.595	I1-Se1-C1 (imidazole)	169.18
Se3-I2	3.858	I2-Se3-C6 (Methyl)	172.10

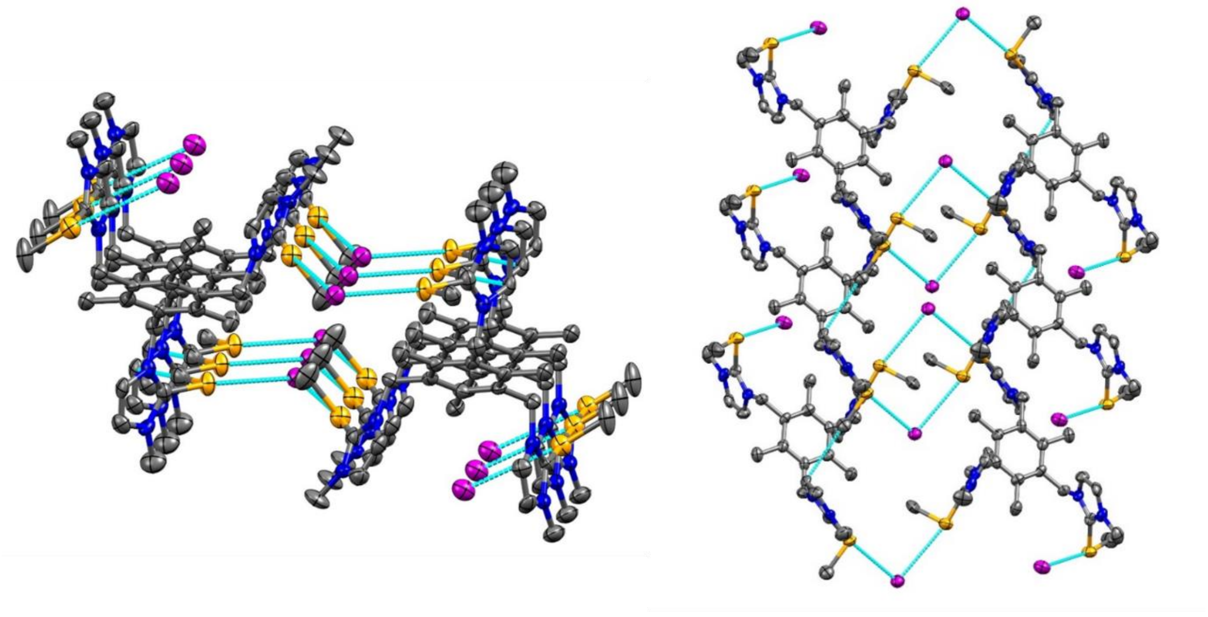

(a) (b)

Figure S59. Different zigzag Crystal packing diagram of **TPI-3Se** iodide crystal, related to Figure 1.

Table S4: Crystallographic details of **TPI-3Se-MI**, related to STAR Methods.

**Compound reference**	TPI-3Se-MI
**Chemical formula**	$C_{23}H_{16}O_3$
**Formula Mass**	1101.25
**Crystal system**	monoclinic
***a*/Å**	7.4483(13)
***b*/Å**	23.936(5)
***c*/Å**	20.584(4)
***α*/°**	90
***β*/°**	97.645(6)
***γ*/°**	90
**Unit cell volume/Å^3**	3637.2(12)
**Temperature/K**	145
**Space group**	*P*21/n
**No. of formula units per unit cell, *Z***	4
**Radiation type**	MoKα
**No. of reflections measured**	38656
**No. of independent reflections**	7966
R_{int}	0.072
**Final R_1 values ($I > 2\sigma(I)$)**	0.0336
**Final $wR(F^2)$ values ($I > 2\sigma(I)$)**	0.0786
**Final R_1 values (all data)**	0.0423
**Final $wR(F^2)$ values (all data)**	0.0827
**CCDC number**	2217469

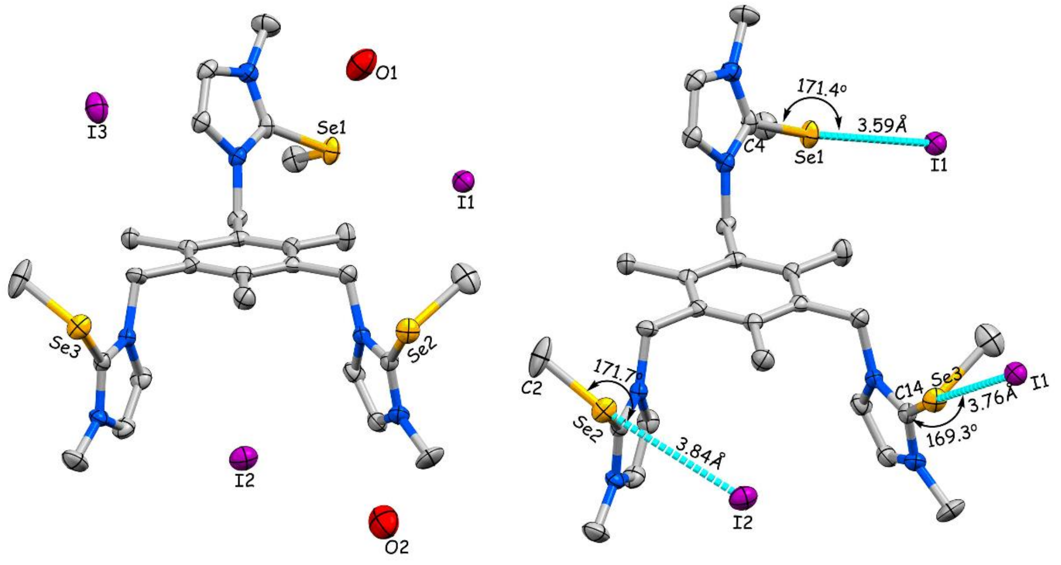

Figure S60. (a) ORTEP diagram of TPI-3Se-MI. Thermal ellipsoids are shown at 50% probability. (b) Solid state structure of TPI-3Se-MI, presented as linear chalcogen bonding interaction of C—Se•••I, related to STAR Methods.

7. Theoretical results:

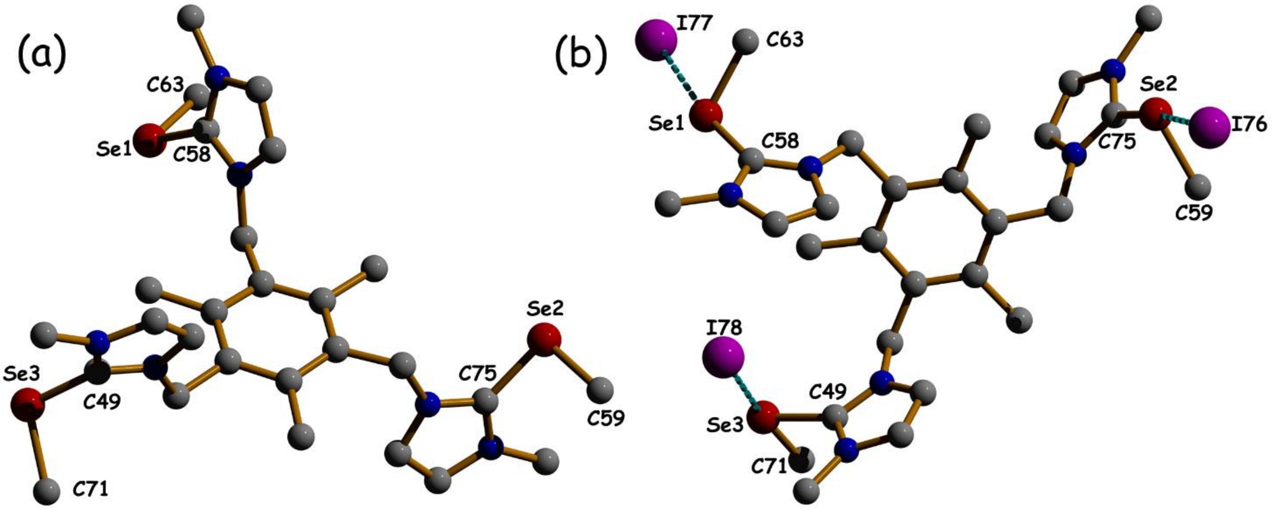

Figure S61. DFT Optimised structure of (a) TPI-3Se (b) TPI-3Se iodide complex, related to STAR Methods.

Table S5. Different Se•••I bond length and I•••Se-C bond angle obtained from DFT optimised structure of TPI-3Se iodide complex, related to STAR Methods.

Bond Lengths (Å)		Bond Angles (°)	
Se1-I77	3.036	I77-Se1-C58 (imidazole)	170.84
Se2-I76	2.946	I76-Se2-C75 (imidazole)	172.83
Se3-I78	2.950	I78-Se3-C71 (Methyl)	172.10

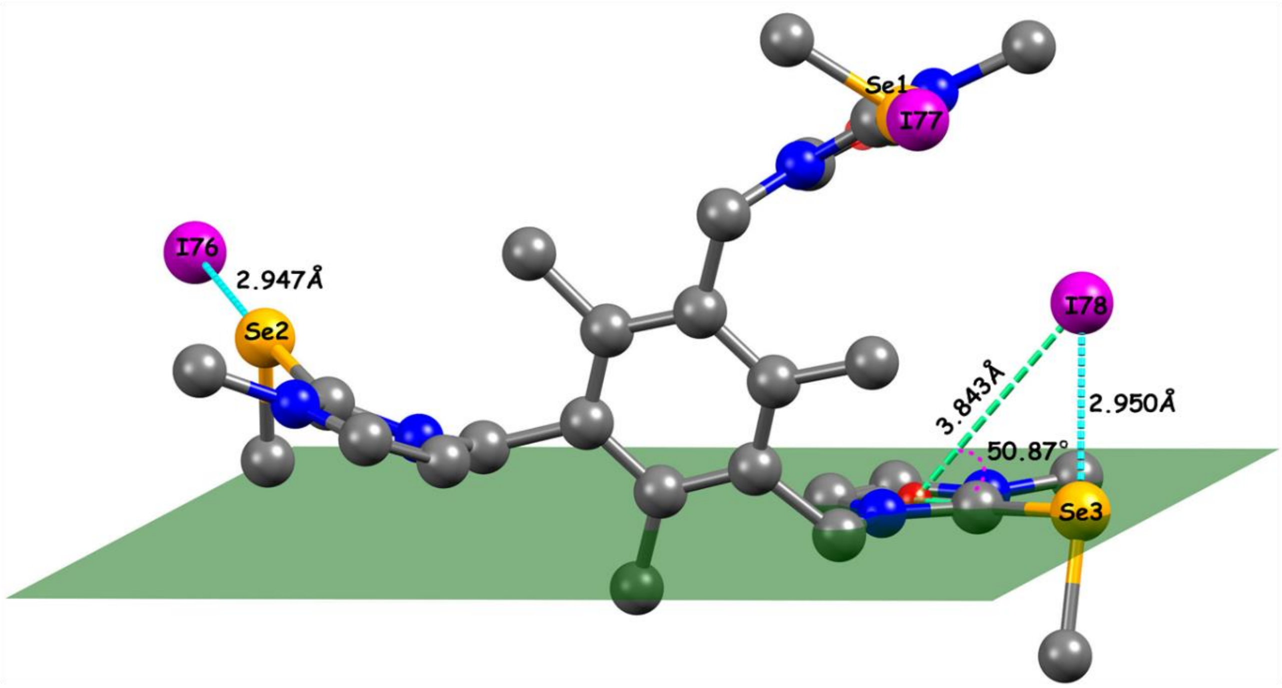

Figure S62. Anion-π interaction between one imidazole ring with I78. (Obtained from the DFT optimised structure of TPI-3Se iodide complex), related to STAR Methods.

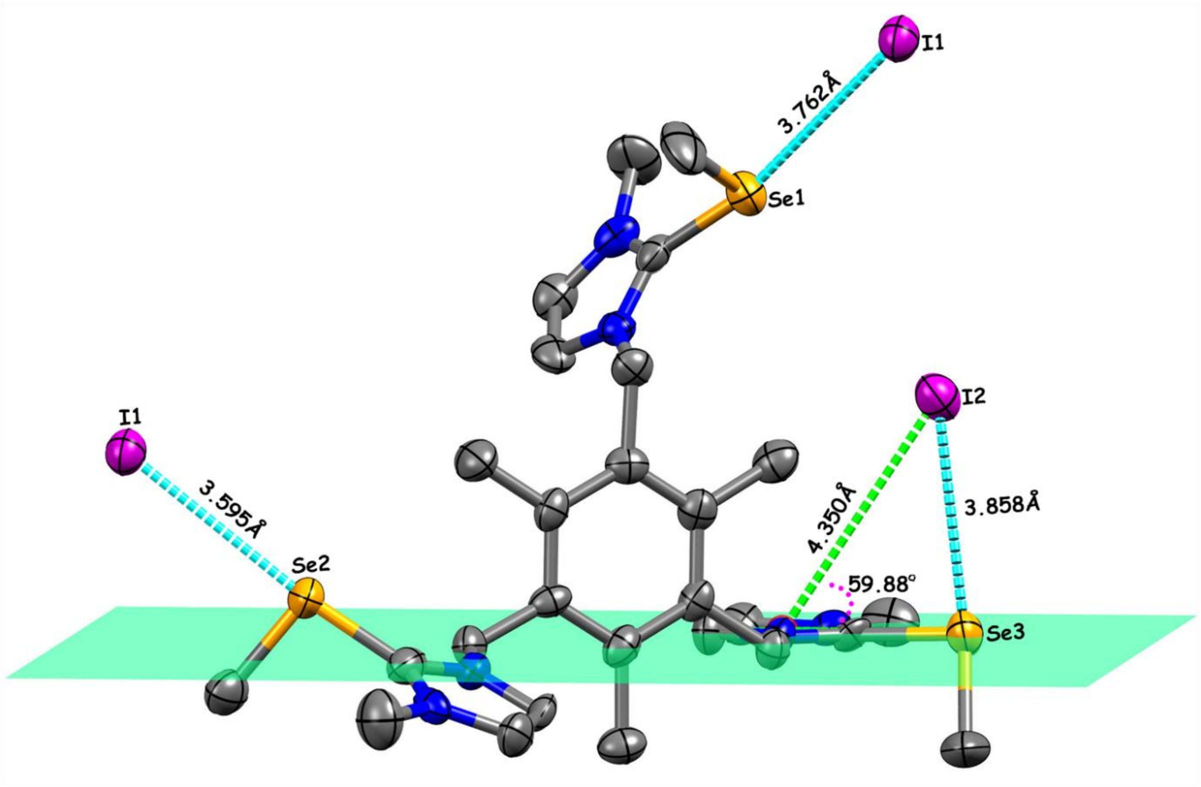

Figure S63. Anion-π interaction between one imidazole ring with I2. (Obtained from the crystal structure of TPI-3Se iodide crystal), related to STAR Methods.

Table S6. Calculated Vs,max (kcal/mol) on the 0.001 a.u. isodensity molecular surfaces associated with the two sigma holes of each three Se atoms of TPI-3Se, related to Figure 2.

Se1	Se2	Se3
166.331 / 179.093	179.88 / 167.382	166.22 / 174.83

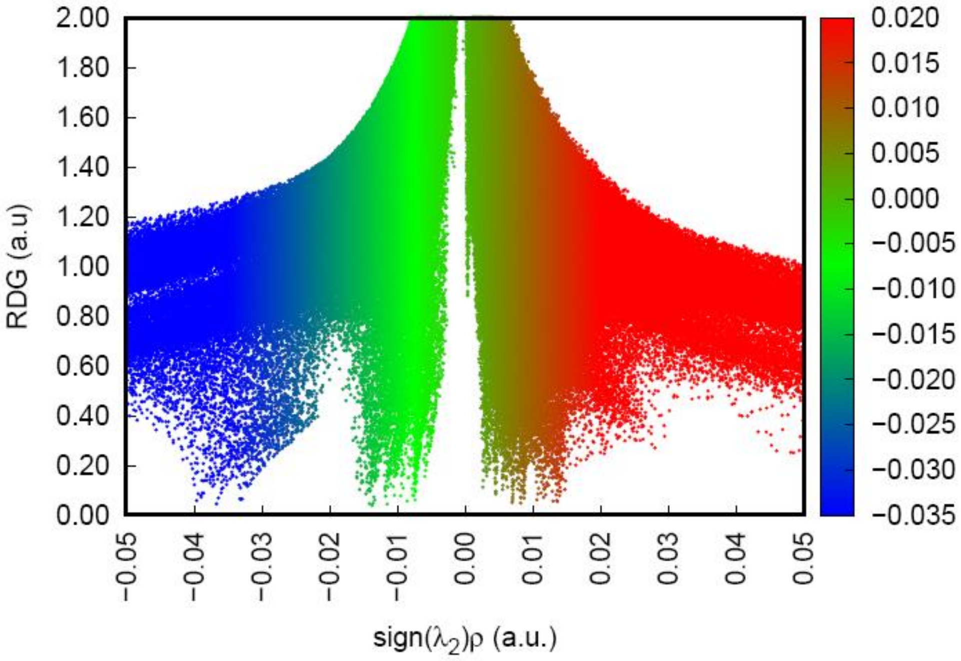

Figure S64. Plot of RDG versus sign(λ_2)ρ, related to STAR Methods.

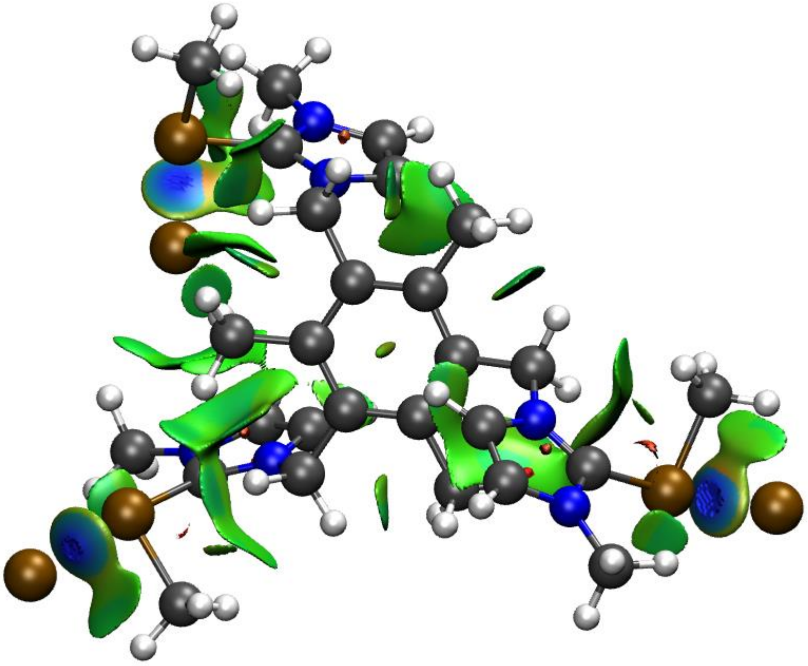

Figure S65. Gradient isosurfaces of TPI-3Se—I, related to STAR Methods.

Table S7. Second order perturbation corresponding donor-acceptor orbital in NBO Analysis, related to STAR Methods.

Donor-Acceptor Orbital	E^2 (kcal/mol)	E(j)-E(i) (a.u.)	E(l,j) (a.u.)
LP(4) I76 to BD*(1)Se2 - C75	56.43	0.32	0.121
LP(4) I77 to BD*(1)Se1 - C58	10.23	0.66	0.074
LP(2) I78 to BD*(1)Se3 - C71	8.74	0.07	0.016

Extraction Studies:

TEM EDS Mapping and EDX Spectra

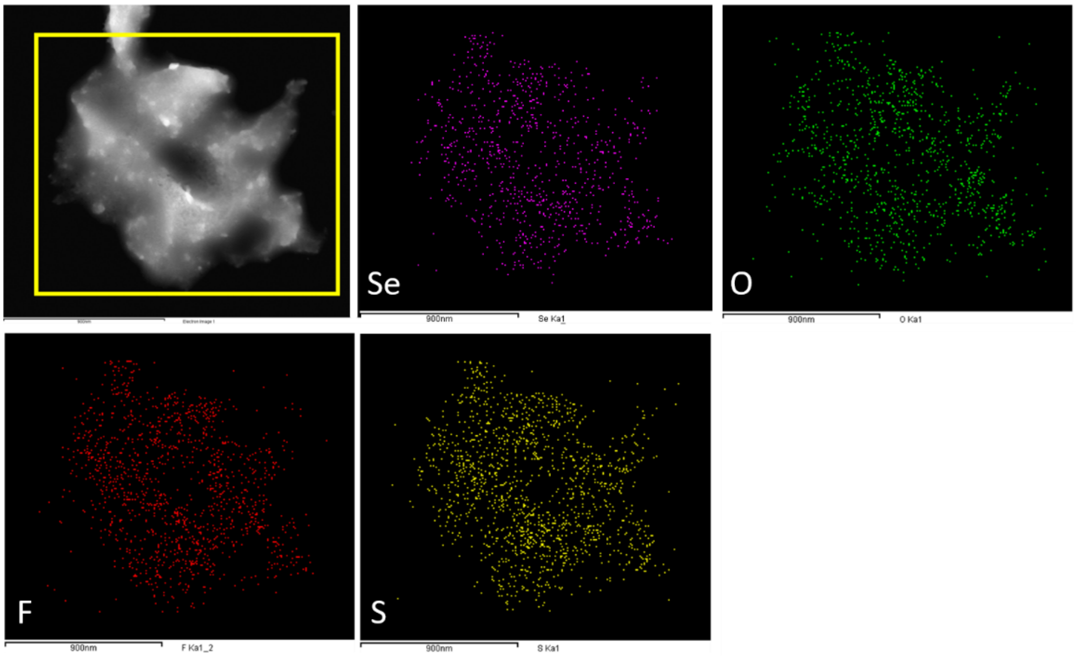

Figure S66. TEM EDS element mapping TPI-3Se, related to Figure 4 and STAR Methods.

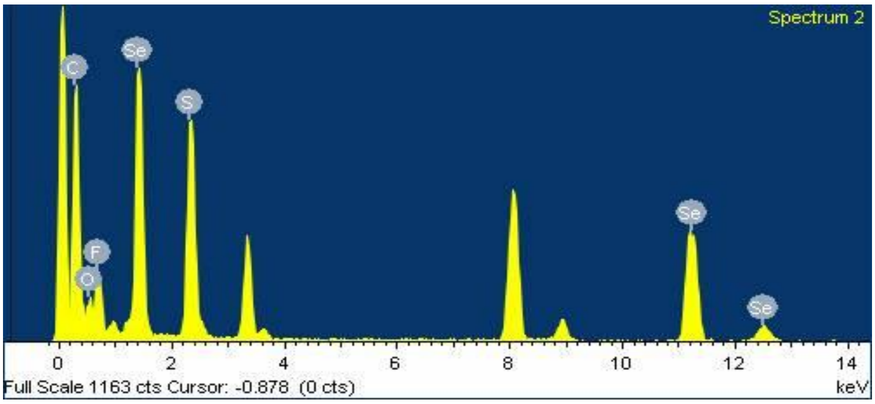

Element	Peak Area	Area Sigma	k factor	Abs Corrn.	Weight%	Weight% Sigma	Atomic%
C K	4289	144	2.209	1.000	35.55	0.89	64.26
O K	503	92	1.811	1.000	3.42	0.61	4.63
F K	1204	90	1.575	1.000	7.12	0.51	8.13
S K	5745	149	0.940	1.000	20.26	0.54	13.72
Se K	4737	117	1.893	1.000	33.65	0.75	9.25
Totals					100.00		

Figure S67. TEM EDX Spectra (Upper) and corresponding elemental measurement(below) of TPI-3Se, related to Figure 4 and STAR Methods.

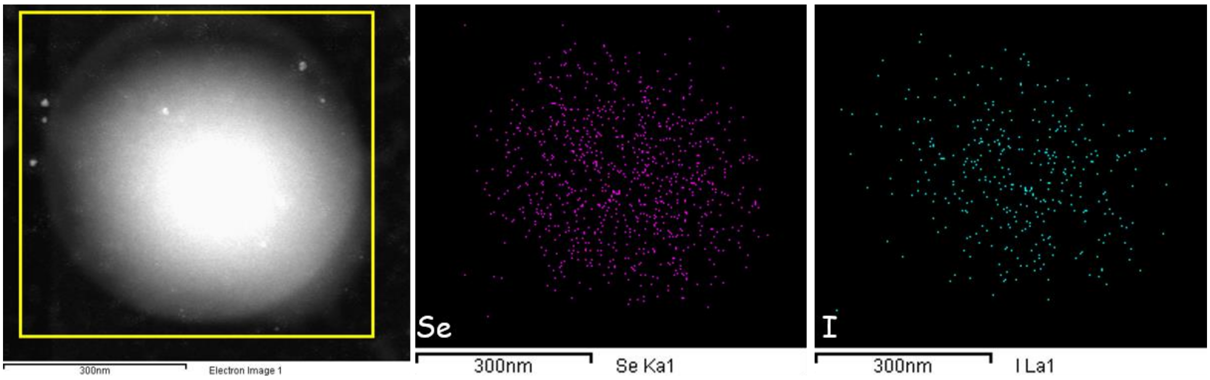

Figure S68. TEM EDS element mapping TPI-3Se-I, related to STAR Methods.

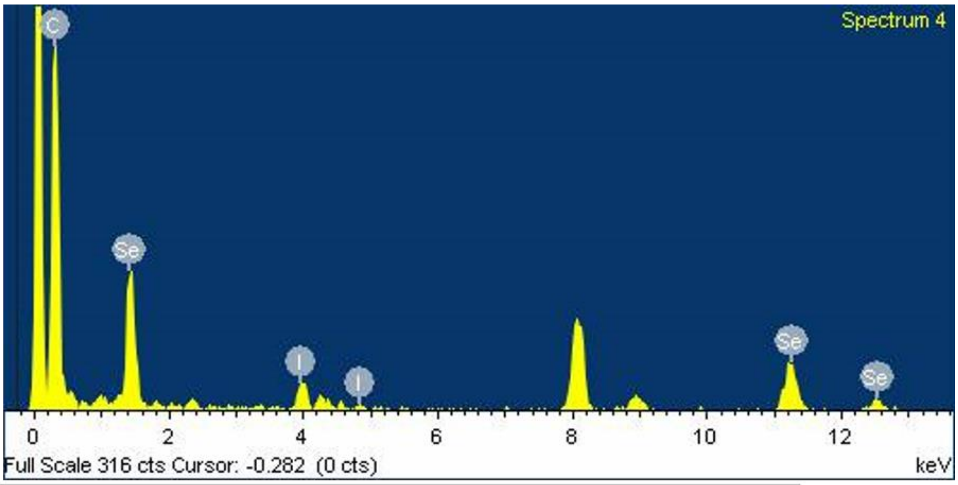

Element	Peak Area	Area Sigma	k factor	Abs Corrn.	Weight%	Weight% Sigma	Atomic%
C K	2622	99	2.208	1.000	62.20	1.36	92.81
Se K	1063	56	1.894	1.000	21.64	1.06	4.91
I L	801	60	1.877	1.000	16.16	1.10	2.28
Totals					100.00		

Figure S69. TEM EDX Spectra (Upper) and corresponding elemental measurement (below) of TPI-3Se-I, related to STAR Methods.

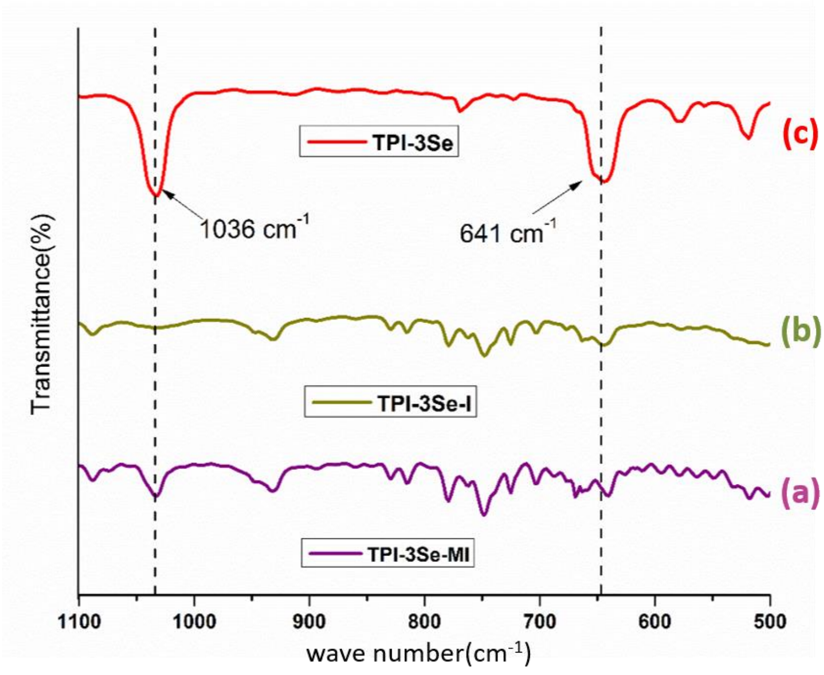

Figure S70. FTIR spectrum of (a) TPI-3Se-MI; (b) TPI-3Se-I; (c) TPI-3Se, related to STAR Methods.

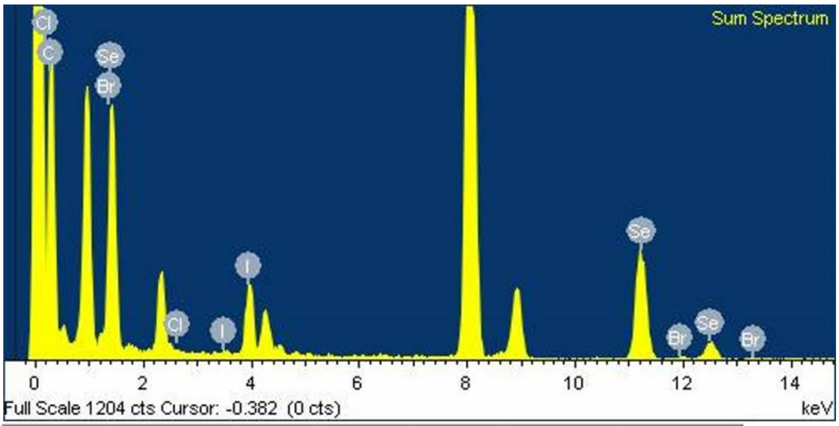

Element	Peak Area	Area Sigma	k factor	Abs Corrn.	Weight%	Weight% Sigma	Atomic%
C K	3702	188	2.209	1.000	34.77	1.26	80.63
Cl K	99	51	0.964	1.000	0.40	0.21	0.32
Se K	4457	116	1.893	1.000	35.87	0.96	12.65
Br K	44	32	1.964	1.000	0.37	0.27	0.13
I L	3584	131	1.877	1.000	28.60	0.95	6.28
Totals					100.00		

Figure S71. TEM EDX Spectra (Upper) and corresponding elemental measurement (below) of TPI-3Se-MI, related to Figure 4 and STAR Methods.

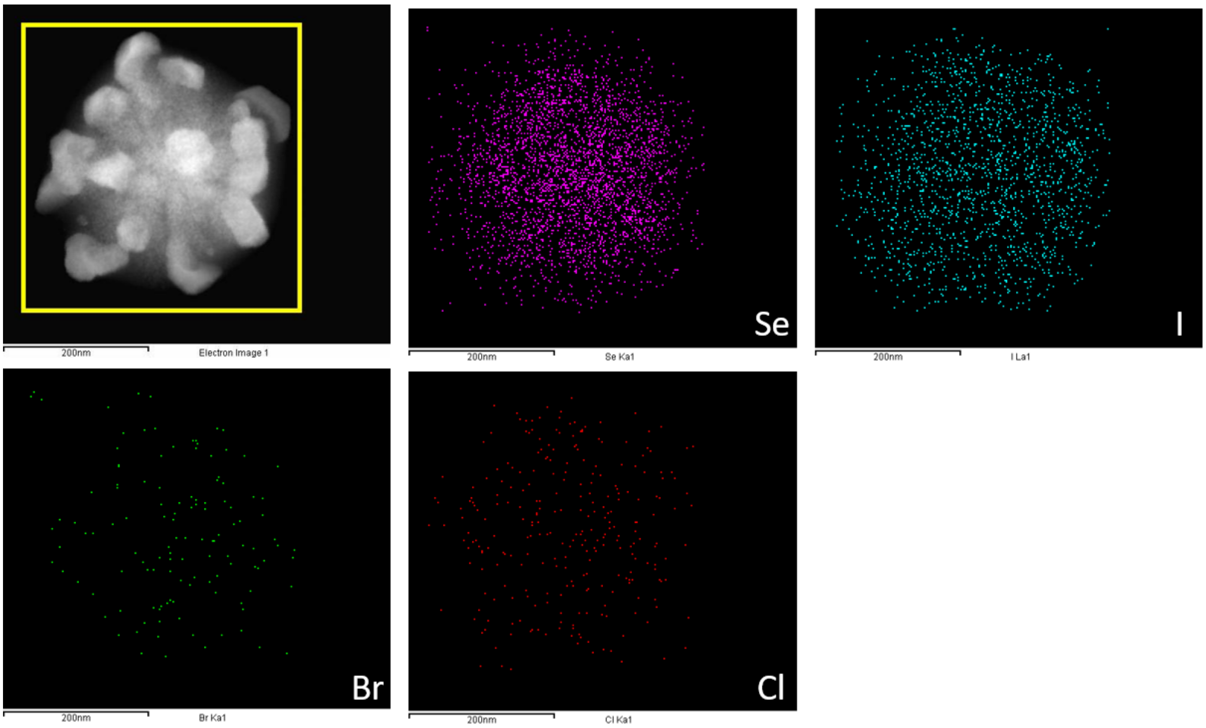

Figure S72. TEM EDS element mapping TPI-3Se-MI, related to Figure 4 and STAR Methods.

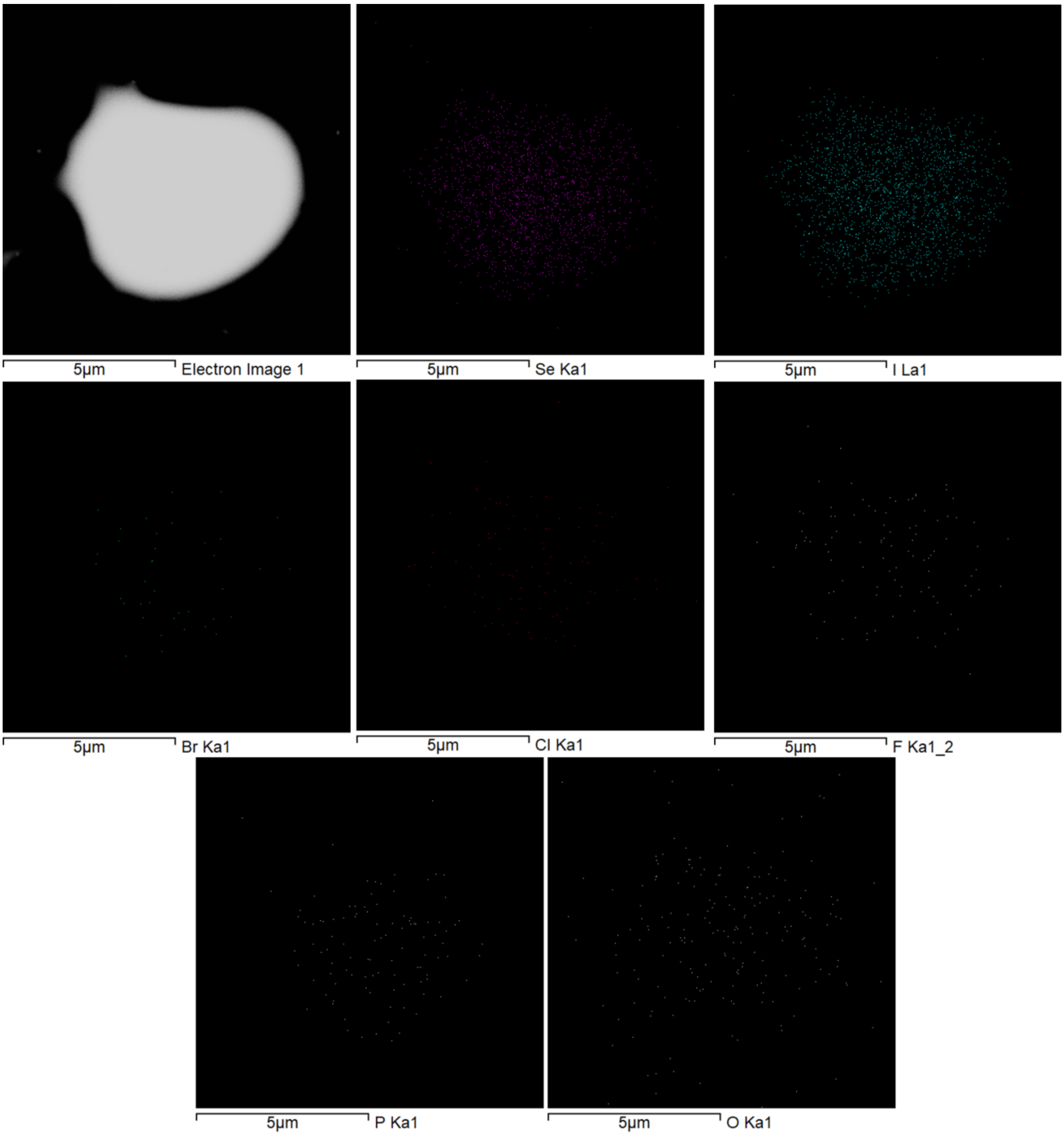

Figure S73. TEM EDS element mapping of TPI-3Se –anion complex. (anions= NaF, NaCl, NaBr, NaI, NaH_2PO_4, NaCN, NaAcO), related to STAR Methods.

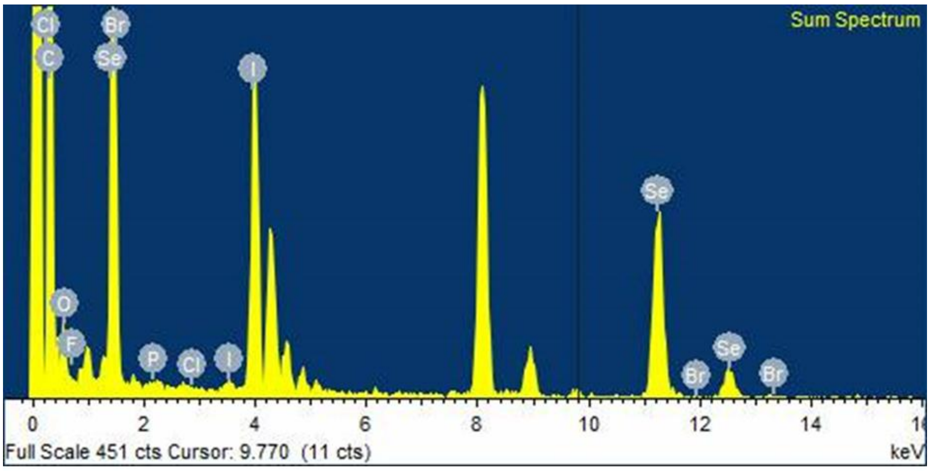

Element	Weight%	Atomic%
C K	32.48	79.30
O K	0.99	1.81
F K	0.07	0.12
P K	0.07	0.07
Cl K	0.10	0.08
Se K	23.38	8.68
Br K	0.23	0.08
I L	42.68	9.86
Totals	100.00	

Figure S74. TEM EDX Spectra (Upper) and corresponding elemental measurement (below) of TPI-3Se–anion complex. (anions= NaF, NaCl, NaBr, NaI, NaH_2PO_4, NaCN, NaOAc), related to STAR Methods.

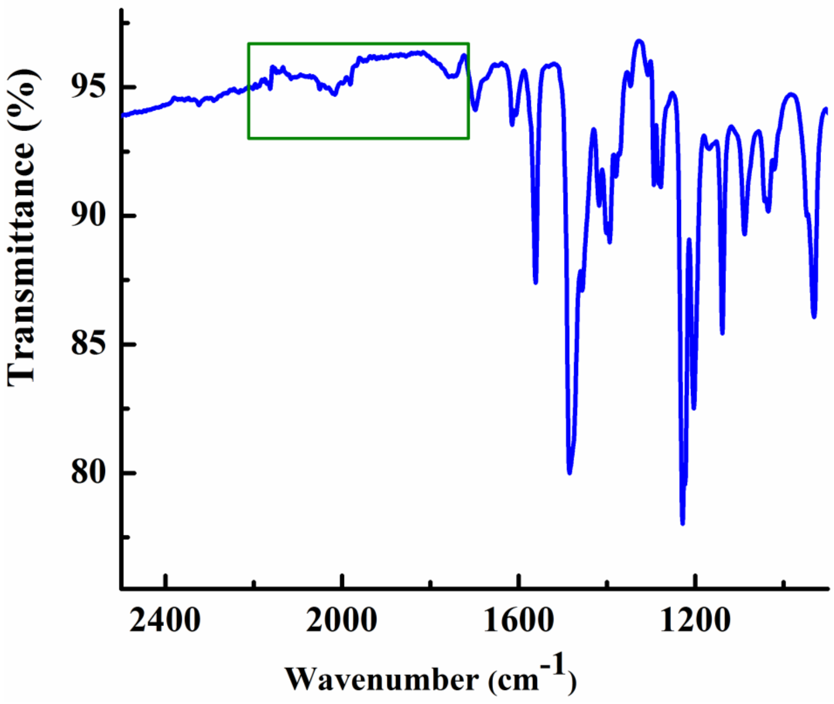

Figure S75. FTIR spectra of TPI-3Se –anion complex. (anion= NaF, NaCl, NaBr, NaI, NaH_2PO_4, NaCN, NaOAc), related to STAR Methods.

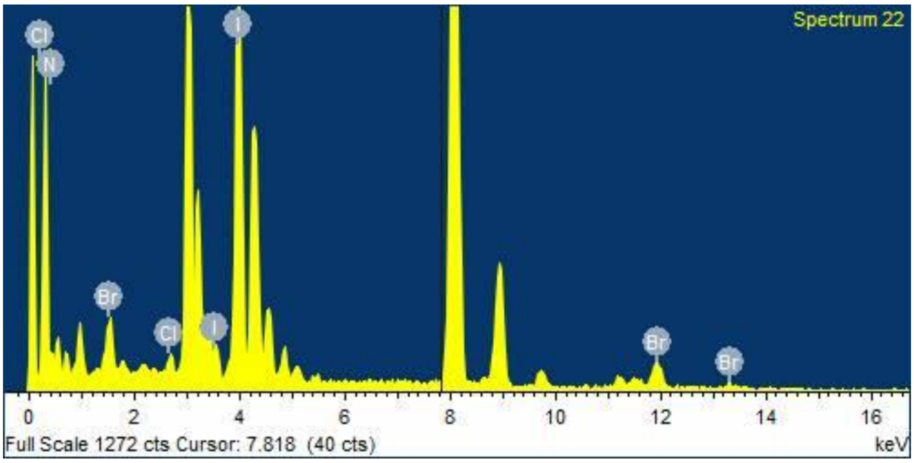

Figure S76. TEM EDX Spectra of precipitated TPI-3H complex after the treatment with equimolar concentration of halides (NaCl, NaBr and NaI), related to STAR Methods.

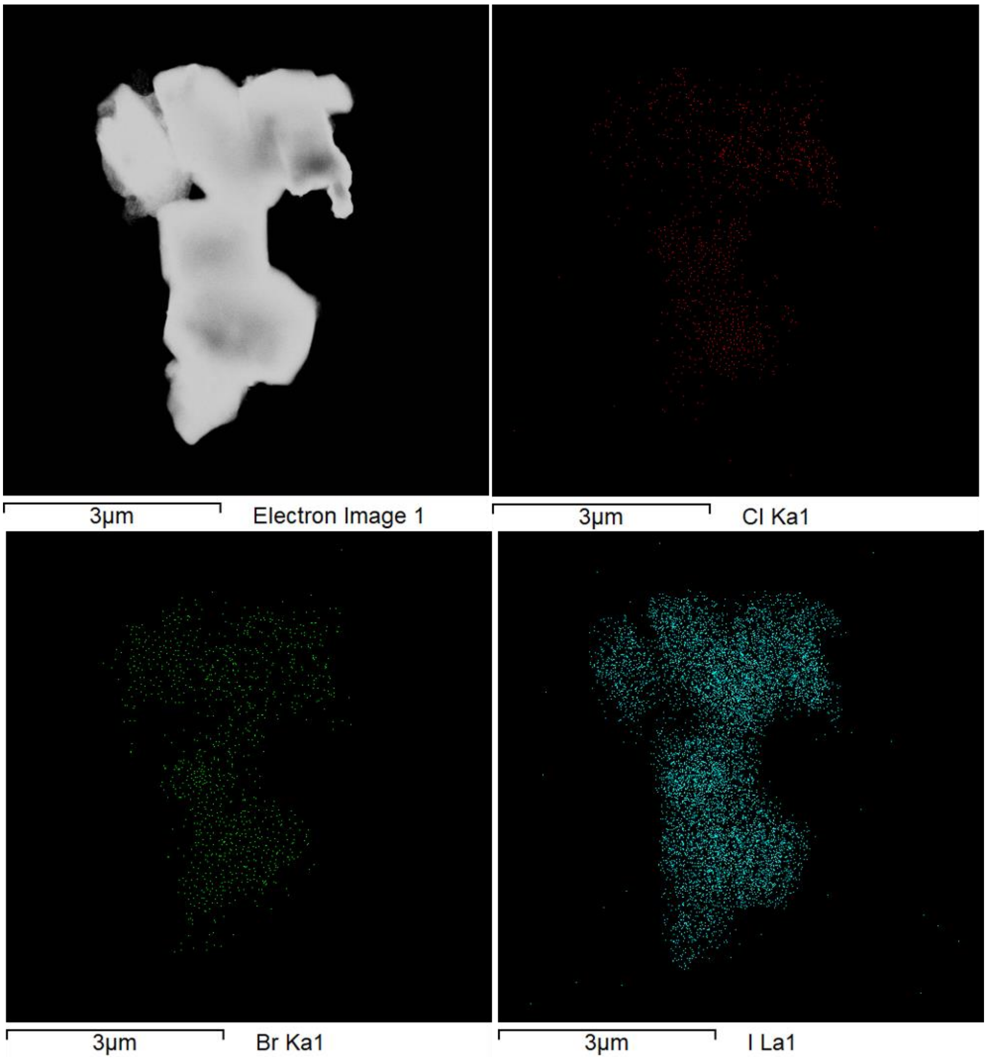

Figure S77. TEM EDS mapping of precipitated TPI-3H complex after the treatment with equimolar concentration of halides (NaCl, NaBr and NaI), related to STAR Methods.

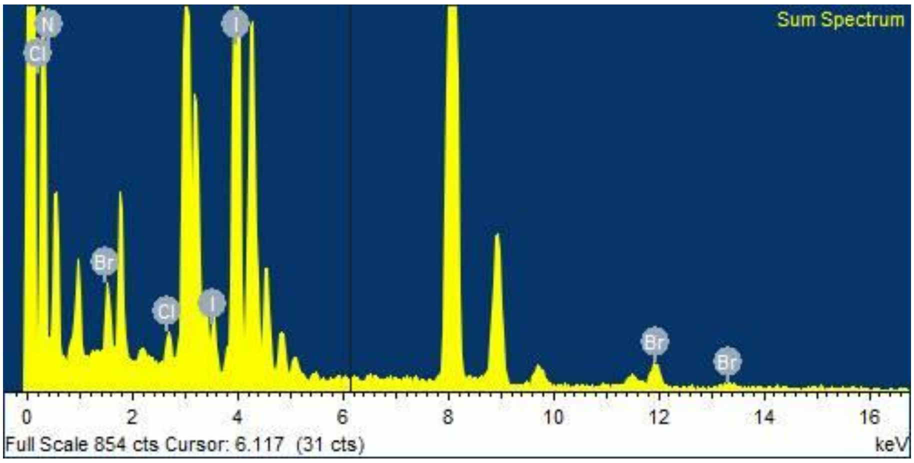

Figure S78. TEM EDX Spectra of precipitated TPI-3I complex after the treatment with equimolar concentration of halides (NaCl, NaBr and NaI), related to STAR Methods.

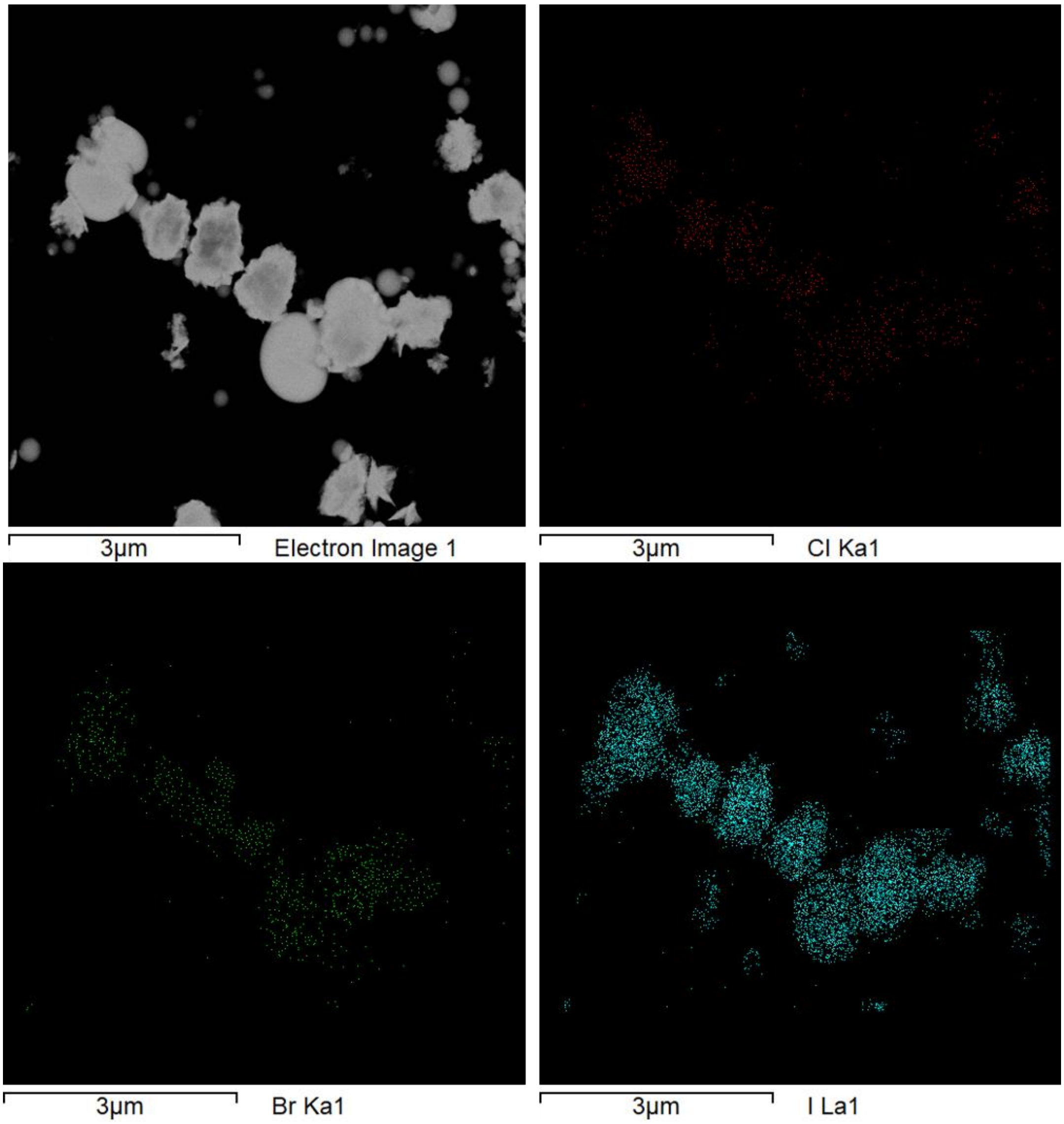

Figure S79. TEM EDS mapping of precipitated TPI-3I complex after the treatment with equimolar concentration of halides (NaCl, NaBr and NaI), related to STAR Methods.

Table S8. pH dependent Extraction table, related to STAR Methods.

Table S8a. Extraction efficiency at pH 3

Initial concentration of I^- (M)	After extraction remaining concentration of I^- (M)	Extraction efficiency for TPI-3Se (%)
0.32	0.097 (2.1)	69.68 (2.5)
0.41	0.122 (1.6)	70.24 (2.1)
0.52	0.150 (2.2)	71.15 (1.9)
0.81	0.243 (2.1)	70.00 (2.4)
1.2	0.358 (1.8)	70.16 (2.2)

Table S8b. Extraction efficiency at pH 10

Initial concentration of I^- (M)	After extraction remaining concentration of I^- (M)	Extraction efficiency for TPI-3Se (%)
0.32	0.102 (2.2)	68.20 (1.8)
0.41	0.126 (2.6)	69.26 (1.6)
0.52	0.156 (2.3)	70.00 (1.9)
0.81	0.250 (2.1)	69.13 (2.2)
1.2	0.370 (2.4)	69.16 (2.1)

- **TEM analysis and PXRD pattern of the iodide complexes obtained by varying iodide concentration as well as pH of the medium**

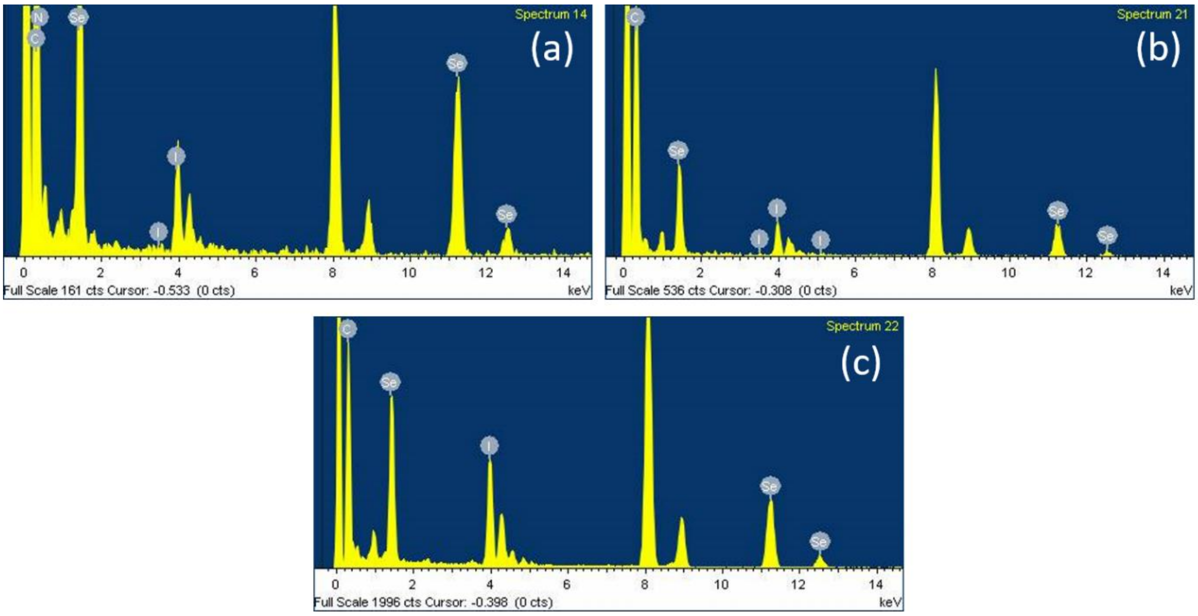

Figure S80. TEM EDX spectra of extracted iodide complexes at pH 3 for (a) 0.3M; (b) 0.5M; (c) 1.2M iodide solution, related to STAR Methods.

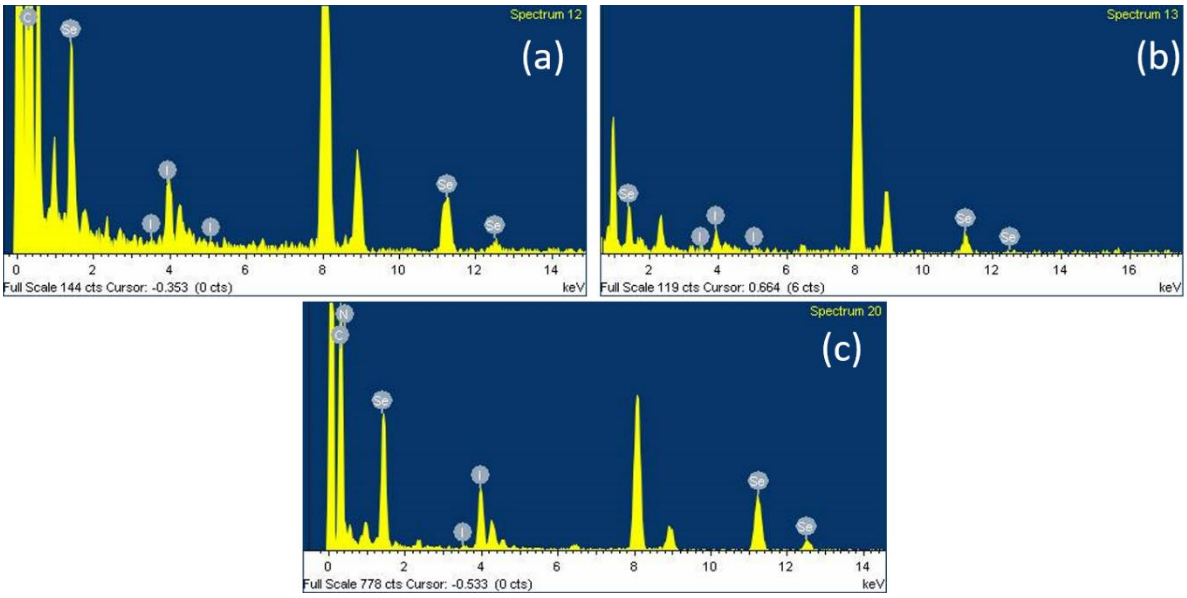

Figure S81. TEM EDX spectra of extracted iodide complexes at pH 7 for (a) 0.3M; (b) 0.5M; (c) 1.2M iodide solution, related to STAR Methods.

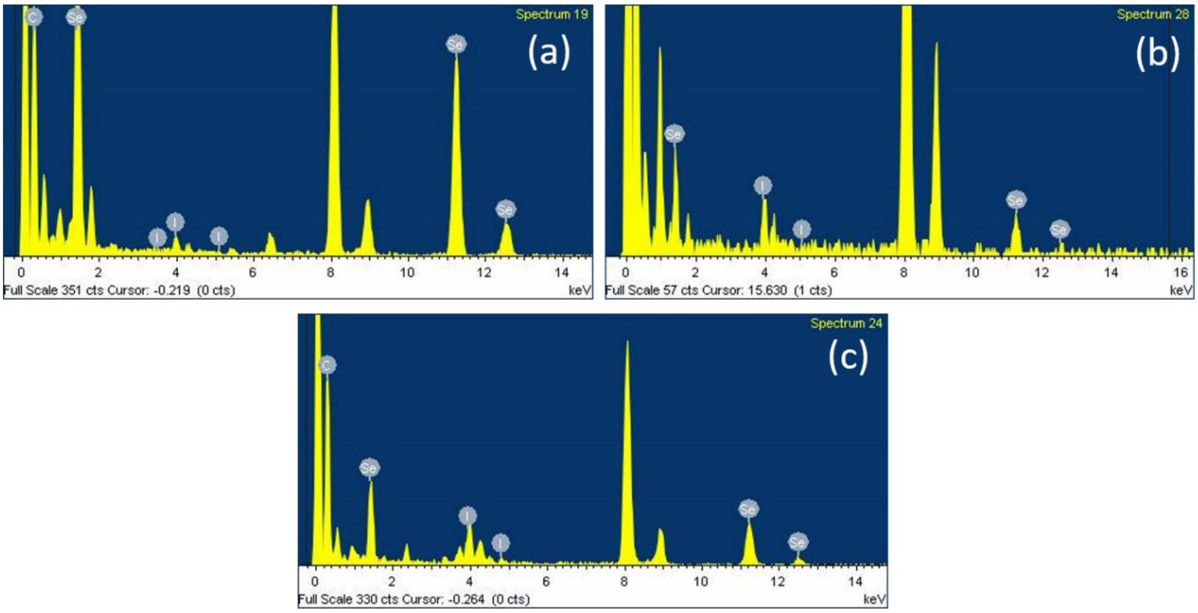

Figure S82. TEM EDX spectra of extracted iodide complexes at pH 10 for (a) 0.3M; (b) 0.5M; (c) 1.2M iodide solution, related to STAR Methods.

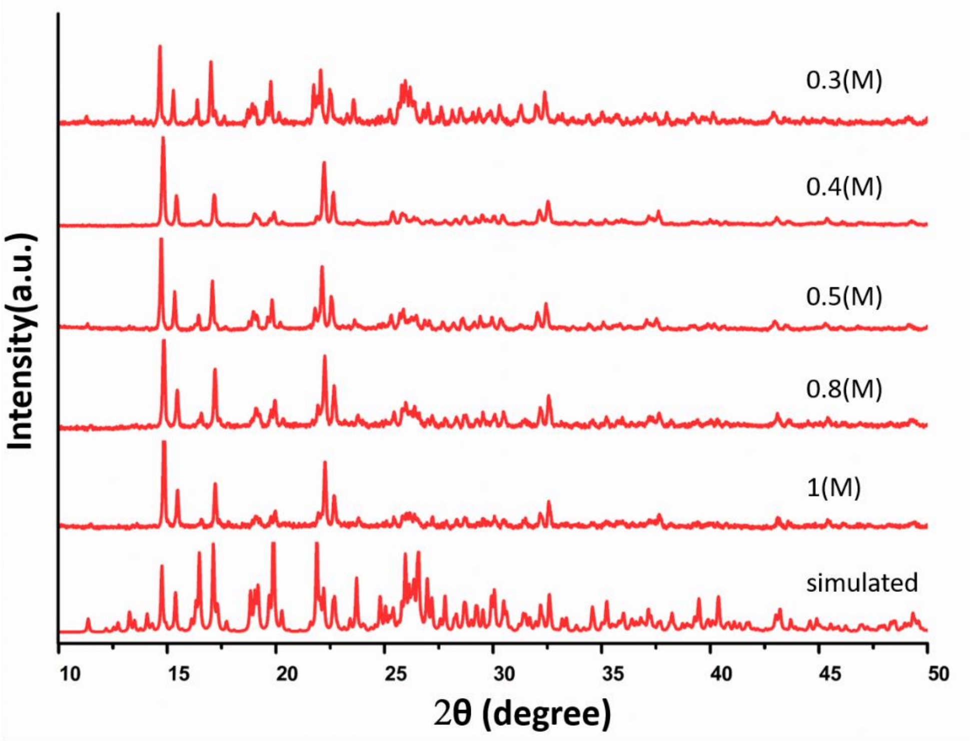

Figure S83. Merged PXRD spectra of the extracted iodide complexes at pH 3 and the simulated pattern obtained from crystal, related to STAR Methods.

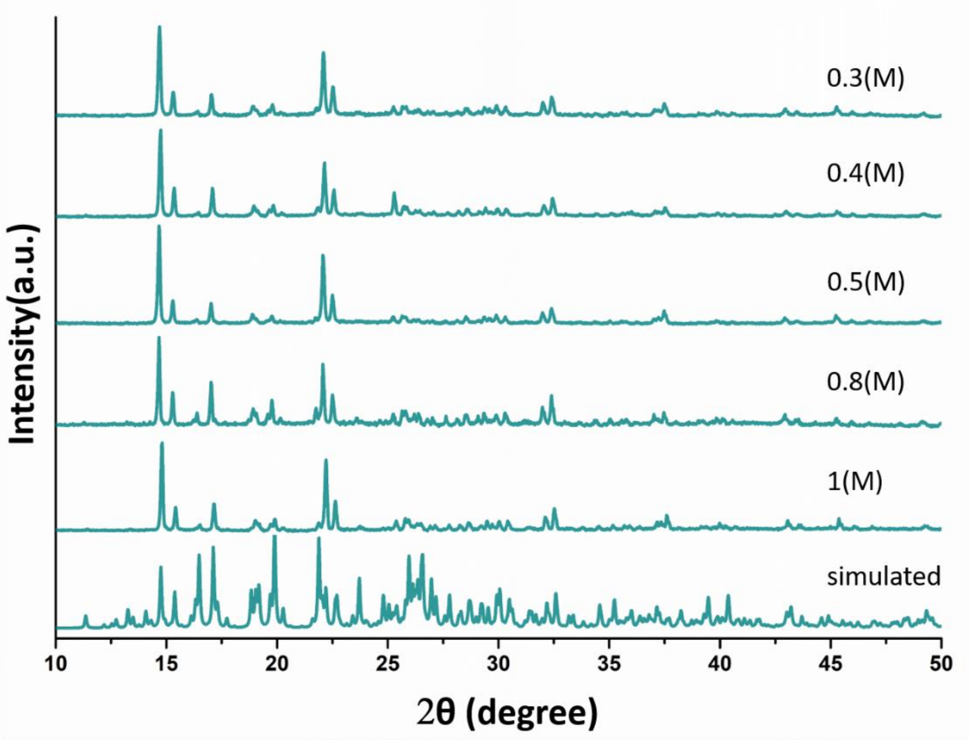

Figure S84. Merged PXRD spectra of the extracted iodide complexes at pH 7 and the simulated pattern obtained from crystal, related to STAR Methods.

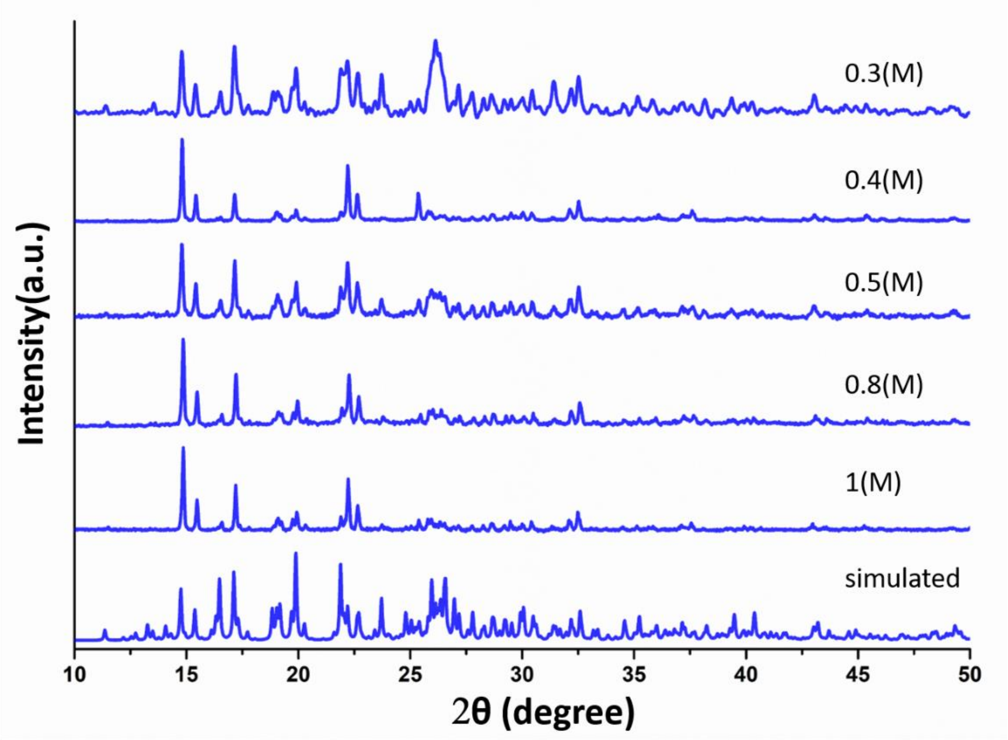

Figure S85. Merged PXRD spectra of the extracted iodide complexes at pH 10 and the simulated pattern obtained from crystal, related to STAR Methods.
